# A First Insight into the Biological Activity Profile of Individual Compounds of *Valeriana officinalis* L.

**DOI:** 10.3390/ijms27198756

**Published:** 2026-09-30

**Authors:** Anastasia Orlova, Alena Soboleva, Anastasiia O. Whaley, Andrei K. Whaley, Darya Meshalkina, Stepan Gambaryan, Sergey N. Smirnov, Margarita M. Shamatova, Yuliya V. Korolkova, Sergey A. Kozlov, Eugene A. Rogozhin, Toni Ditfe, Stanislav Sukhikh, Andrej Frolov

**Affiliations:** 1 Laboratory of Analytical Biochemistry and Biotechnology, K.A. Timiryazev Institute of Plant Physiology, Russian Academy of Sciences, 127276 Moscow, Russia; 2 Laboratory of Bioanalytical Chemistry, Institute of Natural Sciences, Immanuel Kant Baltic Federal University, Universitetskaya 2, 236040 Kaliningrad, Russia; oriselle@yandex.ru; 3 Department of Pharmacognosy, Saint Petersburg State Chemical and Pharmaceutical University, 197376 St. Petersburg, Russia; nasty.ponkratova@spcpu.ru (A.O.W.); 9968639@gmail.com (A.K.W.); 4 Laboratory of Cellular Mechanisms of Blood Homeostasis, Sechenov Institute of Evolutionary Physiology and Biochemistry, Russian Academy of Sciences, 194223 St. Petersburg, Russia; gambaryan.stepan@gmail.com; 5 Interdisciplinary Laboratory of Neurobiology, Sechenov Institute of Evolutional Physiology and Biochemistry, 194223 St. Petersburg, Russia; postoronniiw@yandex.ru; 6 Resource Center of Magnetic Resonance Research Methods, Saint Petersburg State University, 198504 St. Petersburg, Russia; sergey.smirnov@spbu.ru; 7 Shemyakin-Ovchinnikov Institute of Bioorganic Chemistry, Russian Academy of Sciences, 117997 Moscow, Russia; shamatova.m.m@gmail.com (M.M.S.); july@ibch.ru (Y.V.K.); serg@ibch.ru (S.A.K.); rea21@list.ru (E.A.R.); 8 Department of Bioorganic Chemistry, Leibniz-Institute of Plant Biochemistry, 06120 Halle, Germany; toni.ditfe@ipb-halle.de; 9 Institute of Living Systems, Immanuel Kant Baltic Federal University, A. Nevskogo Street 14, 236016 Kaliningrad, Russia; stas-asp@mail.ru

**Keywords:** *Valeriana officinalis* L., *Caprifoliaceae*, iridoids, valerenic acids, antioxidant, anti-coagulant, neuroprotective, TRPV1 ion channel potentiating

## Abstract

*Valeriana officinalis* L. is a widely spread representative of the Eurasian flora. Preparations based on valerian extracts are recognized as sedatives, antidepressants, and anxiolytics by both traditional and modern medicine. Despite the extremely high interest in the biological and pharmacological properties of valerian extracts, the composition of biologically active substances and specific activities of their components are still not completely studied. Therefore, here we address the metabolic profiles of the valerian plants with a special emphasis on their major root constituents with pronounced neuroprotective activity. The following phytochemical analysis of the *V. officinalis* roots led to isolation of nine compounds, including two previously undescribed iridoids—isovaltrate isovaleroyloxyisohydrin (**1**) and isovaltrate *α*-(isovaleroxy)isovaleroxyhydrin (**2**)—together with one previously undescribed valerenic acid derivative—2-oxovalerenic acid (**3**). Three previously described iridoids—iridoidvol A (**4**), isovaltrate isovaleroyloxyhydrin (**5**), and valtrate hydrine B1 (**6**)—along with valerenic acid (**7**) and its metabolites—hydroxyvalerenic (**8**) and acetoxyvalerenic acids (**9**)—were isolated. Functional assays accomplished with the isolated compounds revealed moderate antioxidant activity characteristic for all of them. Moreover, a distinct neuroprotective potential was observed for the compound **3** (and, to a lesser extent, compound **8**). This potential was manifested by a preliminary antineurodegenerative effect in the model of A*β*25–35 induced cytotoxicity. In respect of thrombin-induced platelet aggregation, compounds **1**, **4**, and **7** demonstrated pronounced inhibitory activity, whereas compounds **2**, **5**, and **6** showed distinct potentiating activity. Valerenic acid showed activity on TRPV1 ion channels, manifested by both channel activation and potentiation of the capsaicin response in CHO cells expressing TRPV1. Moderate antioxidant activity was characteristic of all compounds.

## 1. Introduction

The success in discovery and structure characterization of plant secondary metabolites—natural product constituents of extracts prepared from medicinal plants—represents a critical factor in the development of new drugs and therapies. In contrast to primary metabolites, i.e., the “household molecules”, the constitutive levels of secondary metabolites are typically low. However, their contents in plant tissues can rapidly increase in response to environmental and biotic stressors [1]. Thus, these metabolites typically represent effector molecules directly involved in suppression of adverse effects associated with these stressors [2,3]. Due to this pronounced and diverse functional activities of plant secondary metabolites, this group of compounds represent a valuable variety of bioactive substances with potential to treat a wide range of diseases [4,5,6,7].

On one hand, comprehensive phytochemical studies, allow finding new economically efficient sources of well-characterized natural products, on the other—discovering new potent medicinal compounds with high specific biological activities. Analysis of these compounds can give access to natural substances with unique pharmacological properties, typically manifested as antioxidant, anti-inflammatory, antibacterial, anticancer, and other specific biological activities. This fact makes them promising candidates for development of new drugs [8,9,10].

*Valeriana officinalis* L. (*V. officinalis*) is a perennial angiosperm plant from the *Caprifoliaceae* family, native to temperate and subtropical areas in all parts of Eurasia. In nature, *V. officinalis* occurs in lowland meadows, marshes, bogs, along river or lake banks, in clearings and forest edges. *V. officinalis* has a rich history of use in both officinal and traditional medicine. Thus, the soporific and sedative properties of this plant were described already in the works of ancient Greek physicians and in medieval Europe [11,12]. In modern medical practice, tinctures and extracts of valerian roots are actively used as a sleeping and sedative agent [13]. *V. officinalis* is included in the latest editions of the Russian, Belarusian, Kazakh, Japanese pharmacopoeias, as well as the European Pharmacopoeia and the United States Pharmacopoeia [14] as a sedative and anxiolytic agent.

**Figure 1:**
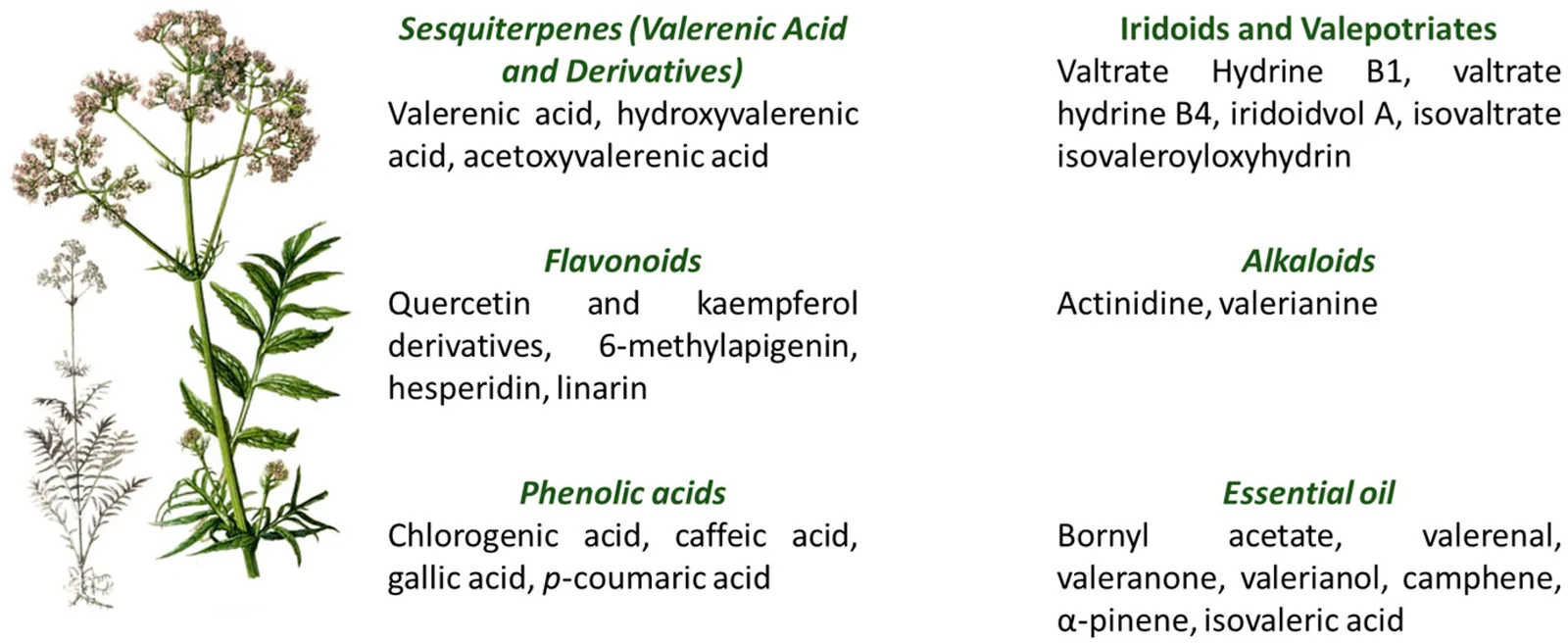
Main metabolite groups occurring in *Valeriana officinalis* L.

*V. officinalis* is a well-known source of multiple secondary metabolites, which play a key role in the pharmacological properties of this plant. The principal natural products of the iridoid nature extractable from the *V. officinalis* roots are presented by valerotriates—a group of non-glycosidic *Valeriana* iridoids—which dominate with valtrate, isovaltrate, homovaltrate, and dihydrovaltrate (Figure 1) [15].

**Figure 2:**
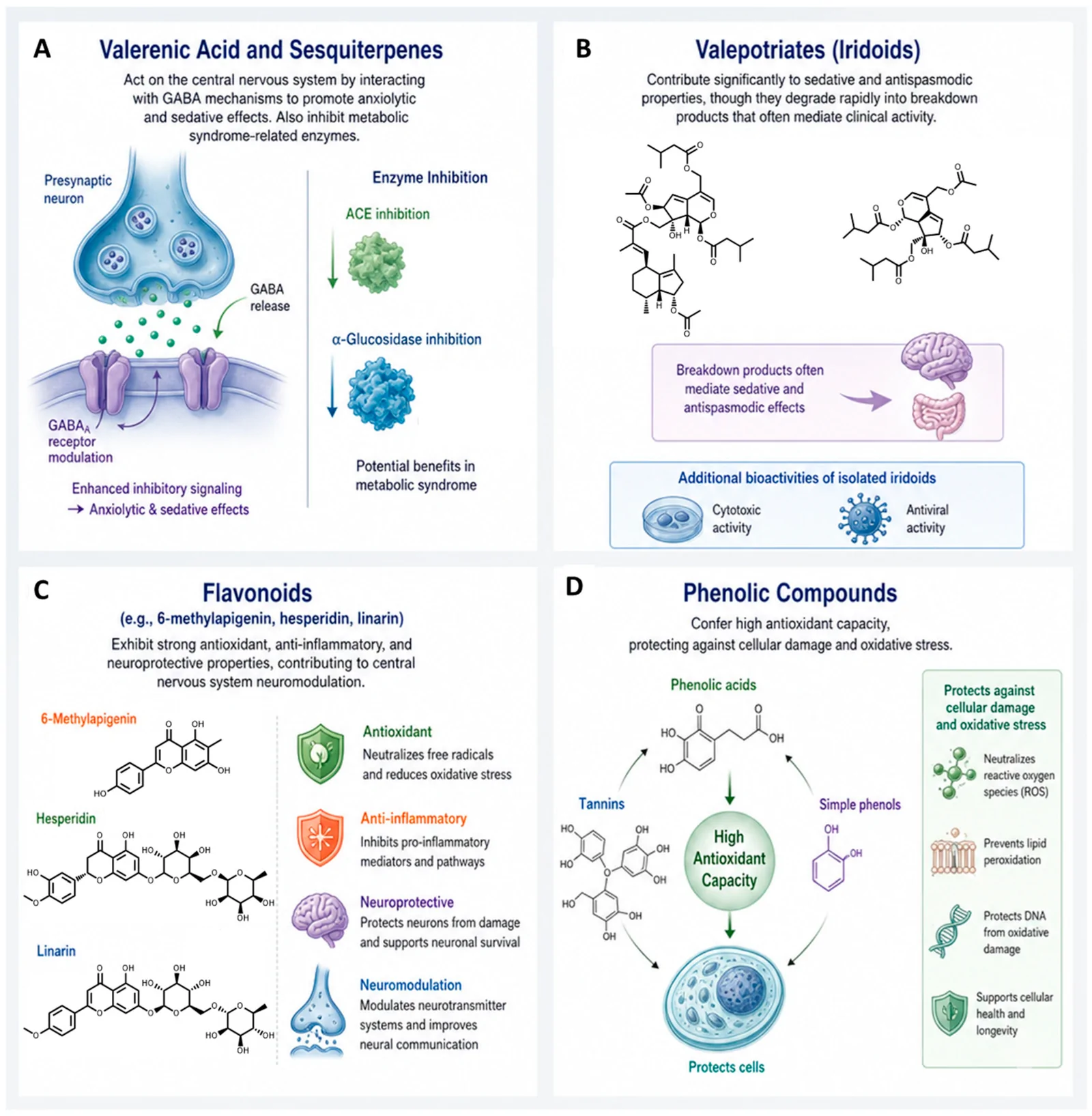
Characteristic bioactivity demonstrated by the principal groups of *Valeriana officinalis* L. metabolites: (**A**) Valerenic acid derivatives are subunit-specific modulators of gamma-aminobutyric acid (GABA) type A (GABA_A_) receptors; (**B**) Iridoids exhibit pronounced sedative and antiviral effects; (**C**) Flavonoids exhibit strong antioxidant activity and are effective in the prevention and treatment of pathologies associated with oxidative stress; (**D**) Phenolic compounds exhibit pronounced antioxidant activity. GABA—gamma-aminobutyric acid; ACE—angiotensin-converting enzyme.

Valeriana iridoids demonstrate a broad spectrum of biological activities. Thus, compounds in this group possess a pronounced sedative effect, reduce locomotor activity, alleviate feelings of fear, anxiety, and aggression, and potentiate the action of hypnotic drugs [16]. Furthermore, they were shown to suppress propagation of the influenza virus, showed pronounced cytotoxicity against cancer cell lines and appeared to inhibit NO production (Figure 2B) [17,18,19,20]. The valerenic acids include three known natural derivatives—valerenic acid, hydroxyvalerenic acid, and acetoxyvalerenic acid (Figure 1)—which were shown to be subunit-specific modulators of *γ*-aminobutyric acid (GABA) type A (GABA_A_) receptors, along with their synthetic derivatives with modified carboxy groups [21,22,23] (Figure 2A).

Despite the fact that the main groups of secondary metabolites of valerian roots are considered to be valepotriates and valerenic acid derivatives, representatives of other chemical groups are also present in total extracts. For instance, some phenolic compounds, including phenolic acids (chlorogenic, caffeic, gallic, coumaric acids) and flavonoids (quercetin and kaempferol derivatives) (Figure 1) [24]. It is well established that phenolic compounds exhibit pronounced antioxidant activity, making this group of compounds promising for the prevention and treatment of pathologies mediated by oxidative stress (Figure 2C,D). Furthermore, pyridine alkaloids (actinidine and valerianine) [12] and an essential oil with a rich composition [25] (Figure 1) have been identified in the aerial and underground parts of *Valeriana officinalis* L.

The broad distribution of *V. officinalis*, as well as the possibility of cultivation of this plant species in the industrial scale, far incompletely studied secondary metabolome of the roots and shoots of the plant, as well as the rich and diverse profile of biological activities of extracts and their isolated constituents, allows considering *V. officinalis* as a promising object for phytochemical research. The impressive scale of producing and application the medicinal preparations based on *V. officinalis* requires advanced and comprehensive studies of their efficiency and safety which rely on the methodology of evidence-based medicine. Therefore, in this work we address biologically active natural products from the *V. officinalis* roots. Based on the results of a phytochemical metabolic profiling study, we isolate nine major secondary metabolites and characterize their structures prior to comprehensive evaluation of their antioxidant, neuroprotective, and anticoagulant activities.

## 2. Results

### 2.1. Preliminary Phytochemical Analysis of the Total Ethanolic Extract Obtained from the Roots of Valeriana officinalis L.

At the first step of our phytochemical studies, we assessed the metabolite profile of the total ethanolic extract of *V. officinalis*. This experiment relied on reversed-phase high-performance liquid chromatography with ultraviolet detection (RP-HPLC-UV at 254 nm), which is appropriate for separation and detection of semi-polar secondary plant metabolites [26]. Thereby, we expected to select the principal major analytes, promising as the targets for further preparative isolation with further adjustment of the strategy for their isolation and purification. The resulted RP-HPLC-UV chromatogram of the total extract from the *V. officinalis* roots is presented in Supplementary Materials (Figure S1). The major constituents of this extract appeared to be relatively nonpolar metabolites (eluting at 65–85% eluent B (0.1% *v*/*v* TFA in acetonitrile), t_R_ more than 30 min), presumably of terpenoid and iridoid nature. More polar components of this extract (eluting at 25–50% eluent B, t_R_ more than 15 min) were also present, and they might be represented with polyphenolic compounds (including phenolic acids and flavonoids), as well as with alkaloids. The latter were, however, observed in the chromatogram only in trace amounts, which were not sufficient for further work on the isolation of these components.

**Figure 3:**
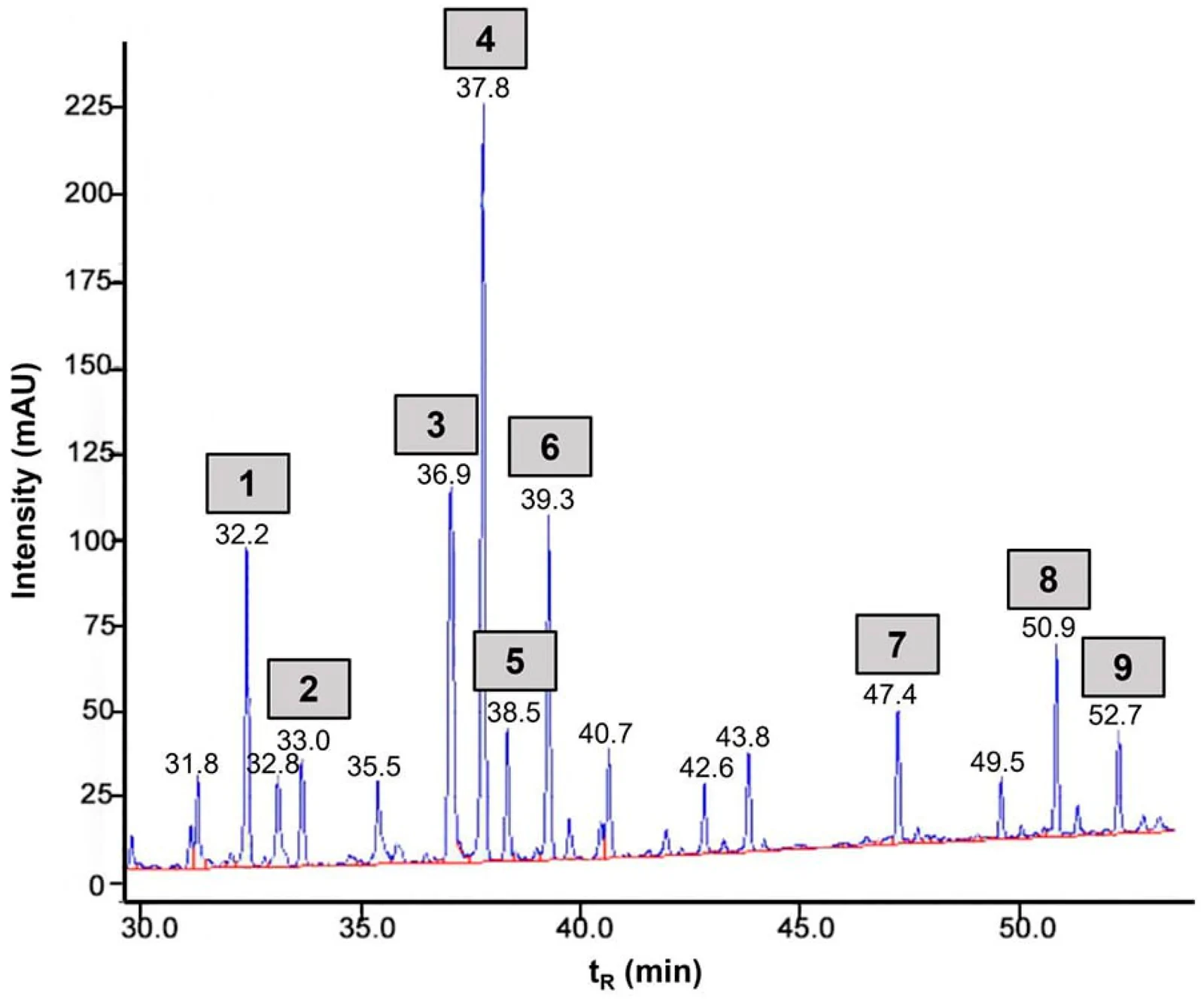
HPLC-UV (254 nm) chromatogram of the dichloromethane fraction of *V. officinalis* **1**–**9**—target compounds selected for further isolation by preparative RP-HPLC-UV.

Thus, the preliminary analysis gave access to the target group of nine compounds of interest (these major species are labeled **1**–**9** in Figure 3) and allowed establishing the strategy for their preparative isolation presented in the next section.

### 2.2. Isolation of Individual Compounds from the Roots of V. officinalis

At the next step, the major compounds annotated from the analytical RP-HPLC-UV chromatogram acquired at 254 nm (**1**–**9**) were isolated from the roots of *V. officinalis* for comprehensive structure characterization and evaluation of their specific pharmacological activities using well-established *in vitro* models. For this purpose, the total ethanolic extract was extracted with an equal volume of dichloromethane. From the obtained fraction dichloromethane was removed under reduced pressure and the dry residue was resuspended in ethanol. The obtained dichloromethane fraction was separated chromatographically on a Sephadex LH-20 column into eight subfractions using ethanol as eluent. Eluting fractions were combined based on TLC analysis in a *n*-buthanol-acetic acid-water (4:1:2) system and RP-HPLC-UV analysis. Subfraction 5 and 6 were further separated by preparative HPLC (the resulting RP-HPLC-UV chromatograms of the separated subfraction is presented in Supplemented Materials (Figure S2A,B), using a gradient solvent system: 0.01–5.0 min 5% B; 5.0–55.0 min 5–95% B; 55.0–55.5 min 95–5% B; 55.5–65.0 min 5% B (eluent A—0.1% *v*/*v* trifluoroacetic acid (TFA) in water, and eluent B—0.1% *v*/*v* TFA in acetonitrile). As a result, a total of four individual metabolites at a sufficient quantity and a purity above 90% were obtained from subfraction 5 (compound **3** with t_R_ 48.1 min, compound **7** with t_R_ 45.2 min [22], compound **8** with t_R_ 42.1 min [22] and compound **9** with t_R_ 29.1 min [22]) and five individual metabolites at a sufficient quantity and a purity above 90% were obtained from subfraction 6 (compound **1** with t_R_ 21.2 min, compound **2** with t_R_ 22.7 min, compound **4** with t_R_ 29.1 min [19], compound **5** with t_R_ 30.2 min [19] and compound **6** with t_R_ 31.8 [19,27]). Based on the RP-HPLC-UV data the purity of all isolated compounds was at least 90% (Table S1). The structures of the isolated individual compounds were assigned by 1D and 2D NMR spectroscopy. The structural identity of all isolated compounds was additionally confirmed by tandem mass spectrometry (MS/MS) in targeted product ion scans acquired with unit Q1 resolution. The MS2 fragmentation patterns acquired for the isolated compounds (Figures S21–S28) were in agreement with the NMR data. Based on the spectral characteristics, six compounds (compounds **4**–**9**) from the group of iridoid glycosides and valerenic acid derivatives previously known in the literature were identified using ^1^H NMR spectroscopic and HR-UHPLC-MS/MS data and the results were shown in Supplemented Material (Tables S2–S7, Figures S23–S28).

**Figure 4:**
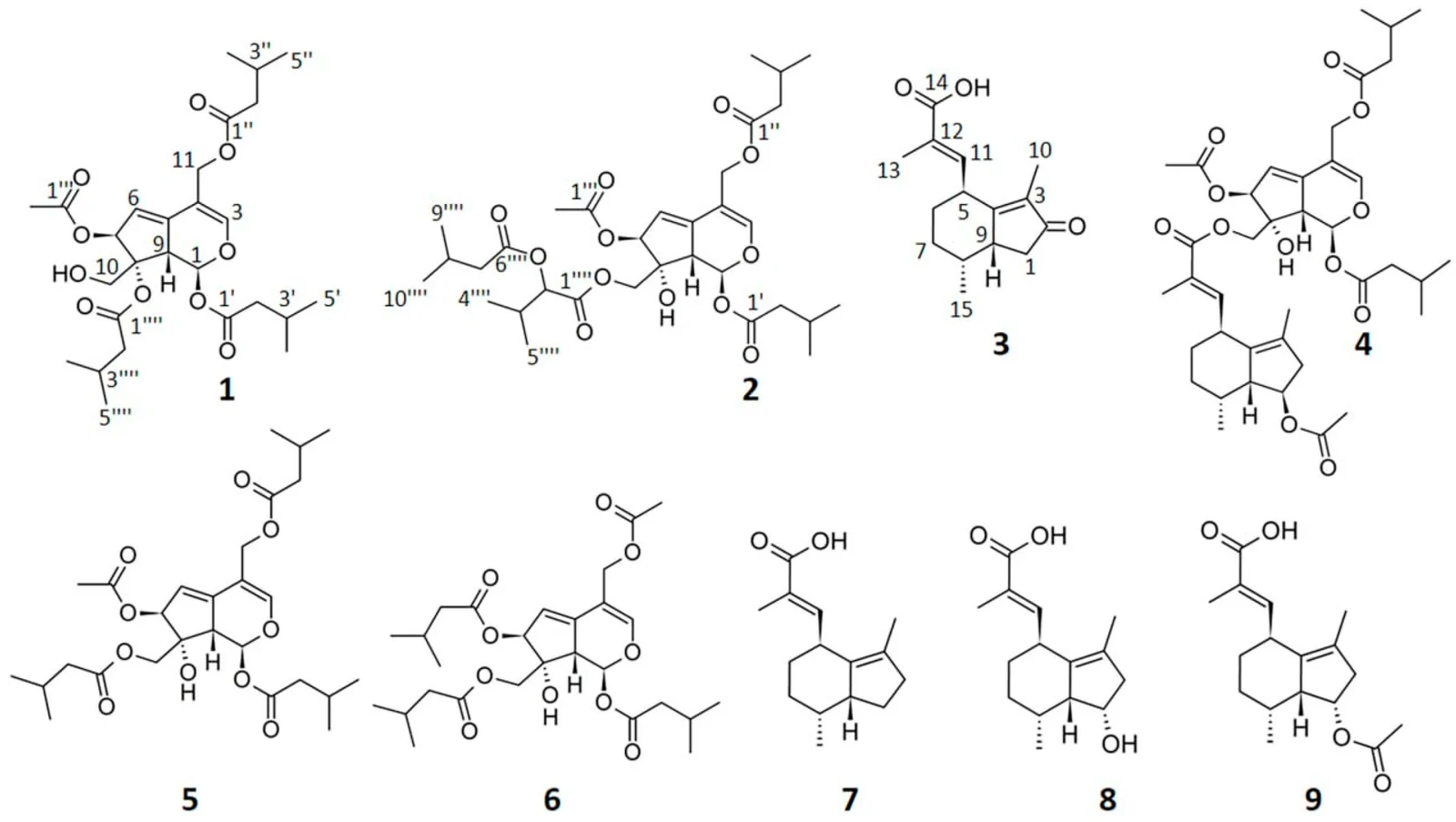
Structures of the major compounds **1**–**9** isolated from the *V. officinalis* extracts assigned based on the NMR and MS/MS data (for details see Supplementary Information—Tables S2–S7, Figures S2–S8): **1**—isovaltrate isovaleroyloxyisohydrin; **2**—isovaltrate α-(isovaleroxy)isovaleroxyhydrin; **3**—2-oxovalerenic acid; **4**—iridoidvol A; **5**—isovaltrate isovaleroyloxyhydrin; **6**—valtrate hydrine B1; **7**—valerenic acid; **8**—hydroxyvalerenic acid; **9**—acetoxyvalerenic acid.

In addition, three previously undescribed compounds were isolated (compounds **1**–**3**), information about which was not previously found in the literature, Dictionary of Natural Products database (https://dnp.chemnetbase.com/), PubChem database (https://pubchem.ncbi.nlm.nih.gov/) or CAS SciFinder Database (https://www.cas.org/). As they appeared to be previously undescribed, their structures were analyzed in more detail using ^1^H and ^13^C NMR spectroscopic (described below) and HR-UHPLC-MS/MS data (Figures S21 and S22). The structures of all isolated compounds, both previously undescribed and known, are shown in Figure 4.

**Figure 5:**
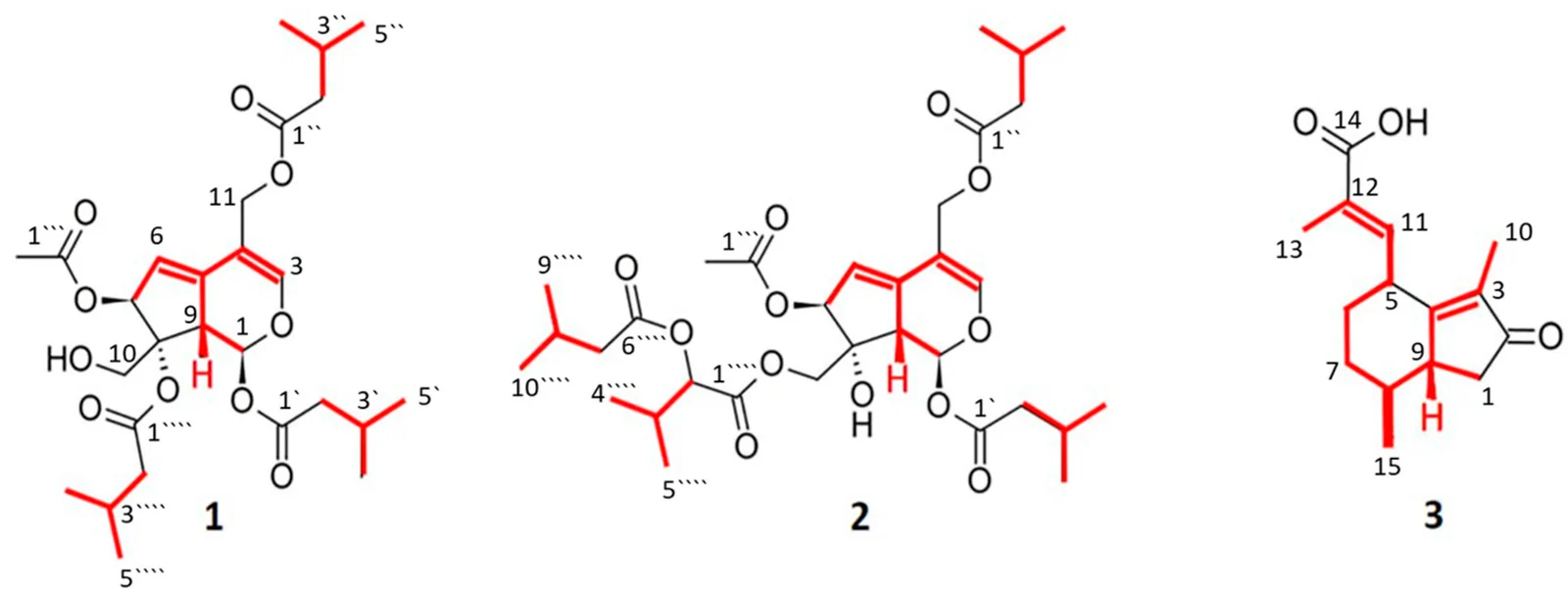
COSY correlations obtained for the compounds **1**, **2**, and **3**.

Compound **1** (t_R_ 21.2 min (here and below is presented the retention time on the preparative HPLC-UV chromatogram (Figure S2))) was obtained as a brown/black amorphic solid. The UV-absorption spectrum showed one maximum at 253 nm. The HR-ESI-MS analysis at the positive ion mode revealed *m*/*z* 525.2653 [M + H]^+^ (calc. *m*/*z* [M + H]^+^ for C_27_H_41_O_10_ −525.2694, 7.8 ppm) that suggested the elemental composition C_27_H_40_O_10_. The ^1^H NMR (Table 1) and COSY spectra allowed assignment of all the observed proton signals to one of two groups containing three spin systems each. The first group of signals included three spin systems from protons that were part of an iridoid core at δH 6.02 (1H, d, J = 10.1 Hz, H-1), 6.91 (1H, s, H-3), 5.65 (1H, dd, J = 3.3, 2.6 Hz, H-6), 5.41 (1H, d, J = 2.6 Hz, H-7), 2.93 (1H, dd, J = 10.0, 2.6 Hz, H-9), which contained two methylene groups displaying diastereotopicity and geminal coupling constants at δH 3.71 (1H, d, J = 11.1 Hz, H-10a), 3.97 (1H, d, J = 11.1 Hz, H-10b) and 4.61 (1H, d, J = 12.3 Hz, H-11a), 4.78 (1H, d, J = 12.3 Hz, H-11b). The second group of signals included protons from three isovaleric acid residues comprising one spin system each at δH 2.34 (2H, dd, J = 9.2, 7.4 Hz, H-2′), 2.04 (1H, m, H-3′), 0.95 (6H, d, J = 6.7 Hz, H-4′/H-5′); δH 2.17 (2H, dd, J = 7.4, 1.2 Hz, H-2″), 1.94 (1H, m, H-3″), 1.90 (6H, d, J = 6.5 Hz, H-4″/H-5″); δH 2.08 (1H, d, J =6.9 Hz, H-2⁗), 1.93 (1H, m, H-3⁗), 0.88 (3H, d, J = 6.9 Hz, H-4⁗), 0.87 (3H, d, J = 6.9 Hz, H-5⁗); and one acetic acid residue at δH 2.00 (3H, s, H-2‴), respectively (Figure 5).

**Figure 6:**
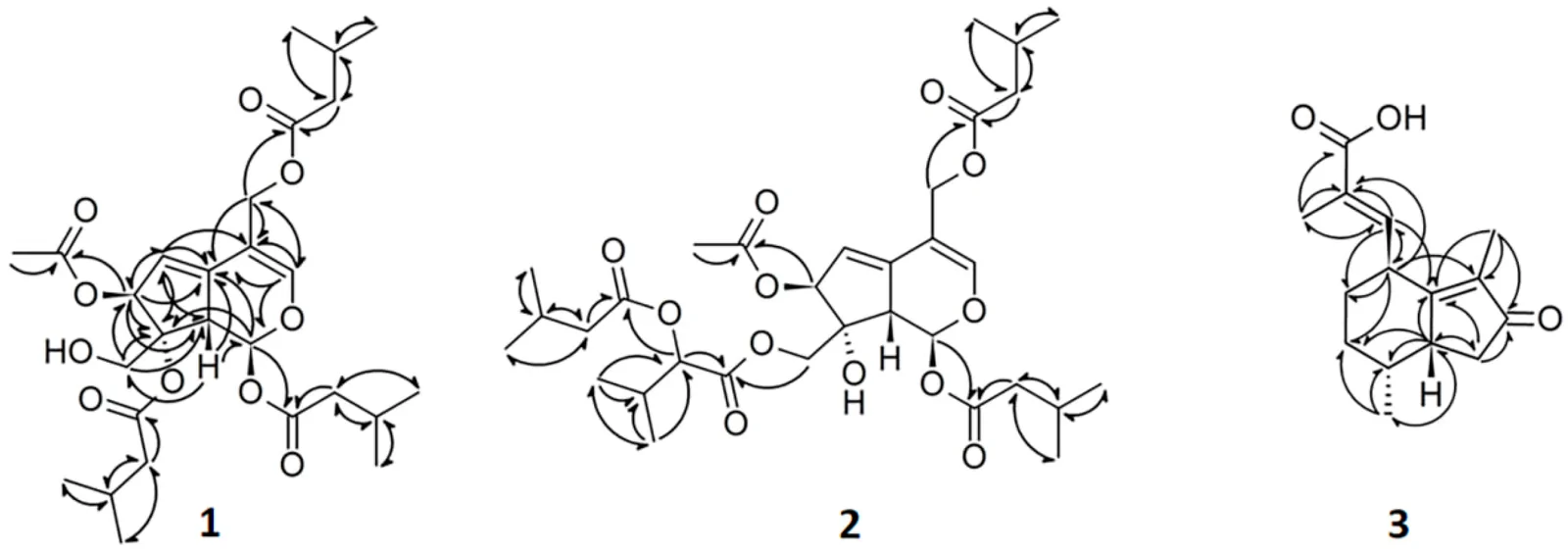
HMBC correlations for the compounds **1** and **3** and the key HMBC correlations for **2**.

The ^13^C NMR data (Table 1) revealed the presence of twenty-seven signals that were assigned to seven methyl groups, five methylene groups, eight tertiary carbons (including two olefinic) and seven quaternary carbons (including four ester, one olefinic and one oxygenated aliphatic carbon) with the help of a DEPT experiment. The above spectroscopic data suggested that compound **1** was a non-glycosylated iridoid four hydroxy groups of which were esterified with three isovaleric acid residues and one acetic acid residue. Considering that the iridoid core contained ten carbon atoms, COSY correlations from H-1 → H-9 → H-6 → H-7 helped in solving part of the structure, which was further supported by HMBC data. HMBC correlations from H-1 → C-5, C-8; H-3 → C-1, C-4, C-5, C-11; H-6 → C-4, C-5, C-7, C-8, C-9; H-7 → C-5, C-6, C-8, C-9; H-9 → C-1, C-6, C-5, C-10; H-10 → C-7, C-8, C-9 and H-11 → C-3, C-4, C-5 confirmed the structure of the iridoid core to be that of valtrate hydrin (Figure 6).

The positions of two isovaleric acid residues and one acetic acid residue were determined based on the HMBC correlations from H-1 → C-1′, H-11 → C-1″ and H-7 → C-1‴, respectively, while the position of the last isovaleric acid residue was determined to be at the iridoid hydroxyl at position 9 through the process of elimination due to no HMBC correlation being present from H-10 to C-1⁗. The relative stereochemistry of the iridoid core was determined through comparison with literature data (Figures S3–S8). Thus, the structure of compound **1** was determined as (1S,7S,8R,9S)-7-acetoxy-1,8,11-triisovaleroxyvaltrate hydrin and given the name isovaltrate isovaleroyloxyisohydrin.

Compound **2** (t_R_ 22.7 min) was obtained as a brown/black amorphic solid. The UV-absorption spectrum showed a shoulder at 221 nm and one maximum at 252 nm. Its positive HR-ESI-MS data *m*/*z* 625.3232 [M + H]^+^ (calc. *m*/*z* [M + H]^+^ for C_32_H_49_O_12_^+^ 625.3219, −2.1 ppm) suggested that the molecular formula was C_32_H_48_O_12_ (Figure S21). Comparison of the ^1^H NMR (Table 2) and COSY spectra of **2** with **1** allowed assignment of the compound 2 as an iridoid derivative as well. It had a structure similar to that of the compound **1**, with a substituent at the hydroxyl in position 10, rather than position 8 in compound **1**. This was indicated by a downfield shift of both H-10 protons to a lower field strength at δH 4.32 (1H, d, J = 11.3 Hz, H-10a) and 4.15 (1H, d, J = 11.3 Hz, H-10b) indicating that the third acid residue of compound 2 was at the iridoid hydroxyl in position 10. This was additionally supported by the displacement of the chemical shift of C-10 to a lower field strength along with HMBC correlations from both H-10 protons to C-1⁗ (Figure 6). The nature of the substituent at the position 10 was confirmed to be *α*-(isovaleroxy)isovaleric acid (Figure 5) based on the downfield shifts of both H-2⁗ and C-2⁗ signals together with the HMBC correlation from H-2⁗ → C-6⁗ (Figure 4 and Figures S9–S14). Thus, the structure of the compound **2** was assigned as 7-acetoxy-10-[*α*-(isovaleroxy)isovaleroxy]-1,11-diisovaleroxyvaltrate hydrin and given the name isovaltrate *α*-(isovaleroxy)isovaleroxyhydrin.

Compound **3** (t_R_ 48.1 min) was obtained as a brown/black amorphic solid. The UV-absorption spectrum showed one maximum at 243 nm. Its negative HR-ESIMS data *m*/*z* 247.1329 [M − H]^−^ (calc. *m*/*z* [M − H]^−^ for C_15_H_19_O_3_^−^ 247.1340, 4.5 ppm) suggested that the molecular formula was C_15_H_20_O_3_ (Figure S22). 1H NMR (Table 3) and COSY spectra showed that compound **3** was a valerenic acid derivative with a single spin system that included all protons in the molecule starting at δH 1.83 (3H, d, J = 1.4 Hz, H-13), 6.94 (1H, dd, J = 9.7, 1.5 Hz, H-11), 3.50 (1H, dd, J = 9.7, 4.1 Hz, H-5), 1.90 (1H, m, H-6a), 1.57 (1H, m, H-6b), 1.49 (1H, m, H-7a), 1.73 (1H, m, H-7b), 2.23 (1H, m, H-8), 0.66 (3H, d, J = 7.0 Hz, H-15), 3.07 (1H, m, H-9), 1.63 (3H, d, J = 1.8 Hz, H-10), 2.30 (1H, dd, J = 18.7, 6.8 Hz, H-1a) and 2.04 (1H, dd, J = 18.7, 1.2 Hz, H-1b) (Figure 5). The ^13^C NMR (Table 3) spectrum contained fifteen signals that were assigned to three methyl groups, three methylene groups, four tertiary carbons (including one olefinic) and five quaternary carbons (including one carboxylic acid, three olefinic and one keto group) with the help of a DEPT experiment. HMBC correlations from H-13 → C-11, C-12, C-14; H-11 → C-4, C-5, C-6, C-13, C-14; H-5 → C-3, C-6, C-7, C-11, C-12; H-15 → C-7, C-8, C-9; H-9 → C-1, C-3, C-7, C-6, C-11, C-15; H-1 → C-2, C-4, C-8, C-9 and from H-10 → C-2, C-3, C-4 showed that the keto group was present at the position 2 of the sesquiterpene core (Figure 6). Therefore, the structure of compound **3** was assigned as 2-oxovalerenic acid, the first characterized derivative of valerenic acid containing a keto group in its structure (Figures S15–S20).

### 2.3. Antioxidant Activity of the Individual Compounds Isolated from the Roots of V. officinalis

**Figure 7:**
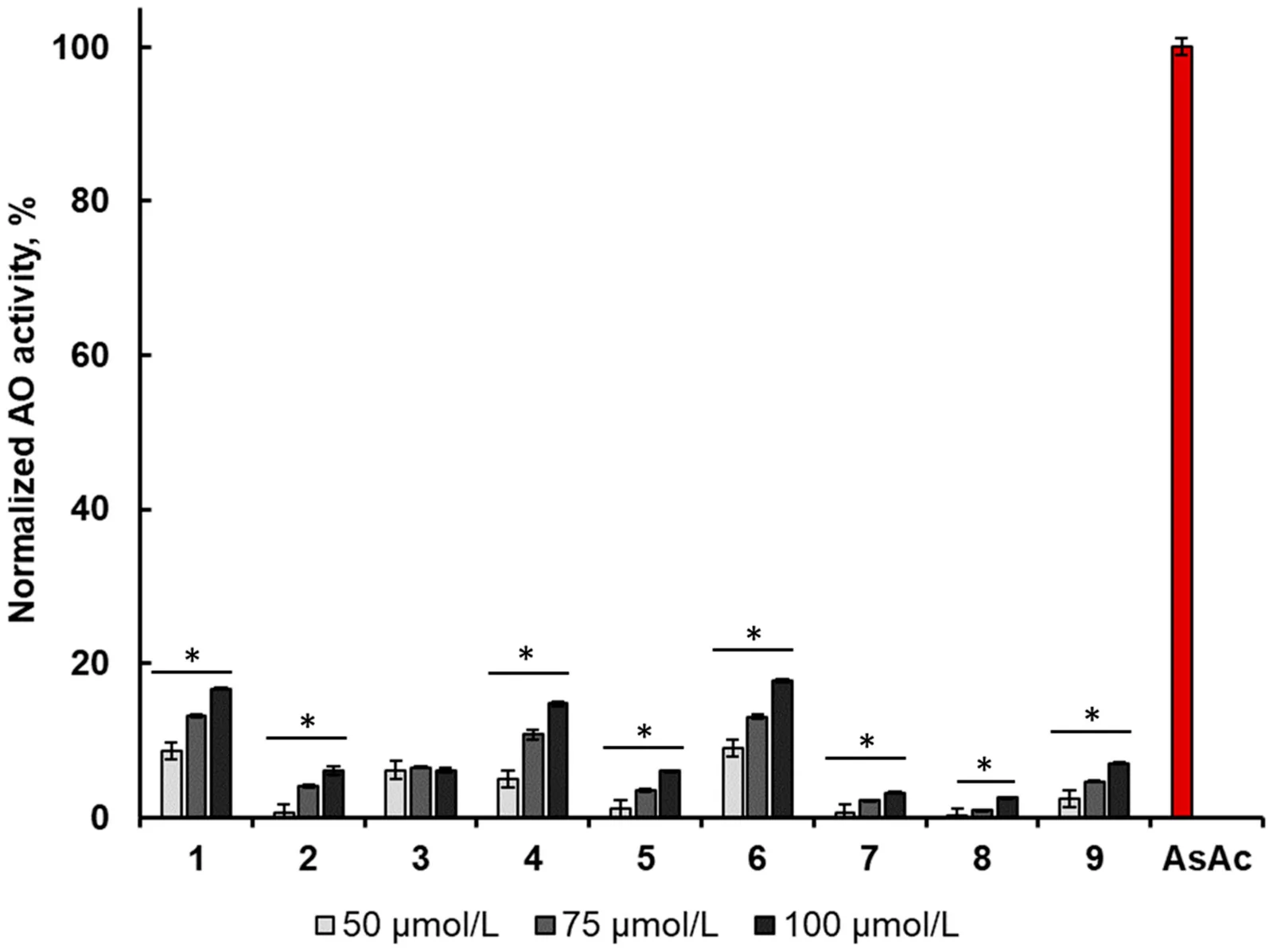
Normalized to ascorbic acid antioxidant activity of compounds isolated from *V. officinalis* roots in DPPH test. Labeling of the compounds corresponds to the numbering in Figure 2. Data are presented as mean ± SD (*n* = 3). AsAc—ascorbic acid. The symbol ‘*’ indicates statistical significance of the differences at the confidence level of *p* ≤ 0.05 for ANOVA and Turkey post-hoc test, *n* = 3.

The antioxidant activity of the metabolites was assessed by the 2-diphenyl-1-picrylhydrazyl (DPPH) test. The optimal concentration range for individual compounds for the assay was roughly optimized on compound **4** ranging from 10 *μ*mol/L to 100 *μ*mol/L. This concentration range was in the linear dynamic range (LDR); therefore, 50 *μ*mol/L, 75 *μ*mol/L, and 100 *μ*mol/L concentrations were chosen for other purified compounds. As can be seen from Figure 5, the antioxidant activity of the isolated compounds demonstrated clear dose-dependence (except compound **3**). However, no molecule exceeded a 17% level when it was normalized to the activity of the positive control (ascorbic acid) (Figure 7), and the observed response was assessed as low.

### 2.4. Neuroprotective Activities of the Individual Compounds Isolated from the Roots of V. officinalis

**Figure 8:**
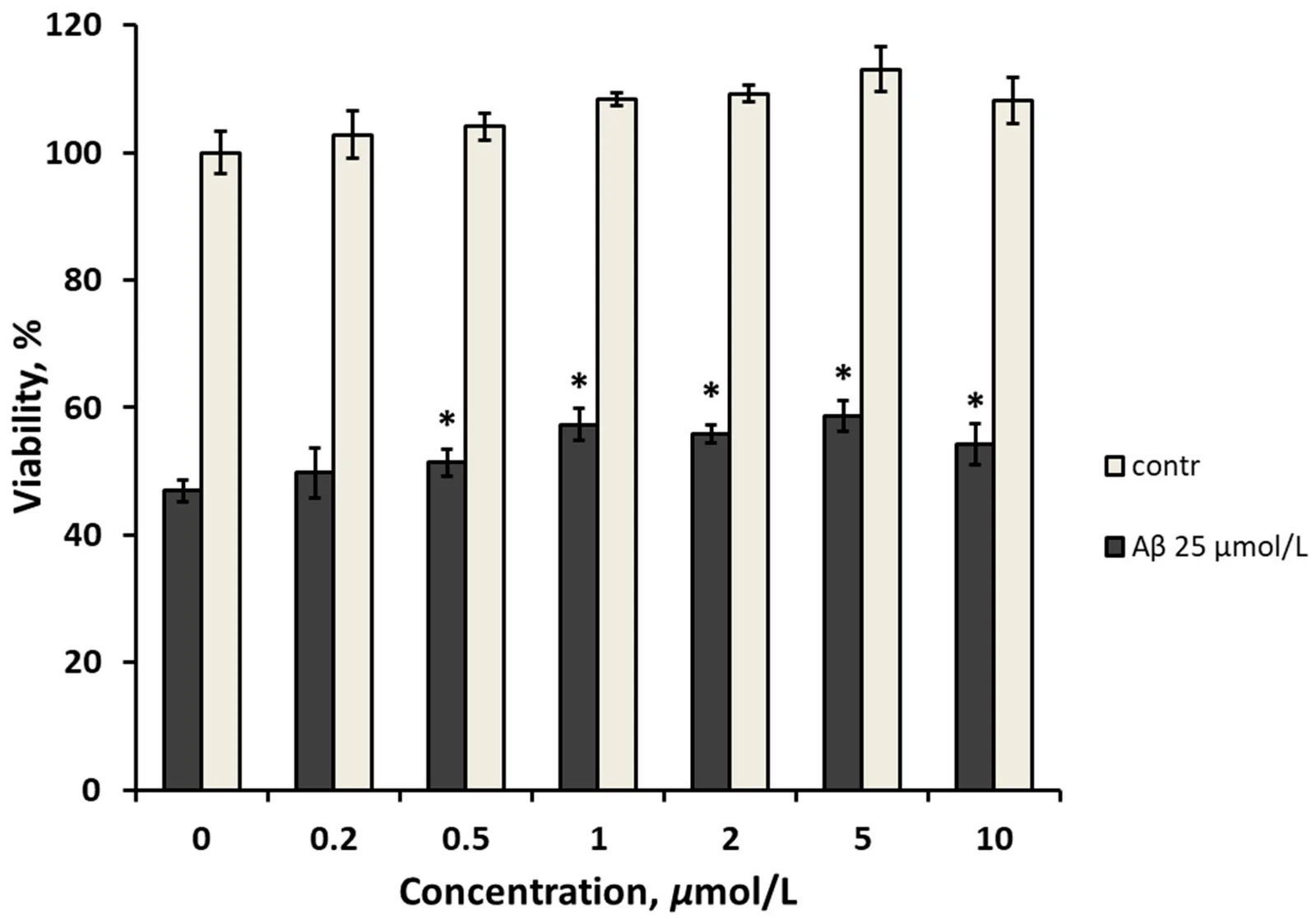
Neuroprotective effects of individual compounds isolated from the roots of *Valeriana officinalis* L. in the model of A*β*25–35 induced cytotoxicity. The data is shown for the compound **3**, demonstrating prominent protective effect. White bars depict the culture viability under control conditions, i.e., without supplementation of A*β*25–35, black bars depict culture viability after supplementation of 25 *μ*mol/L A*β*25–35. The symbol ‘*’ indicates statistical significance of the differences to the controls (cells treated with A*β*25–35 only) at the confidence level of *p* ≤ 0.05 for ANOVA and Dunnett post-hoc test, *n* = 4.

In total, six individual compounds isolated from the roots of *V. officinalis* were assessed for neuroprotective activity (compounds **2**–**4**, **6**, and **8**–**9**). Because of the low amounts of the obtained material (which were insufficient for comprehensive biological tests), compounds **1**, **5**, and **7** were not employed in these experiments. The neuroprotective properties of the *V. officinalis* isolates were addressed in the model of A*β*25–35 induced cytotoxicity in the SH-SY5Y differentiated cell culture. Thereby, the neuroprotective activity of the compound **3** appeared to be most pronounced. The neuroprotective effects of this compound were significant in a broad concentration range (0.5–10 *μ*mol/L) and reached nearly 10% of viability enhancement when 1–5 *μ*mol/L of the compound were applied. Importantly, the compound **3** did not show any toxicity under our experimental conditions. Moreover, it showed a slight stimulation effect improving culture viability (Figure 8).

Compound **2** demonstrated relatively high cytotoxicity, which was clearly manifested already at 2 *μ*mol/L. The protective effects of the compounds **2** and **8** did not reach statistically significant levels. Further, the compounds **4** and **6** also appeared to be cytotoxic. Their toxicity could be observed at concentrations comparable to those shown as efficient for the compound **2** (compound **4** at 1 *μ*mol/L and compound **6** at 2 *μ*mol/L), but no neuroprotective effect of these compounds could be demonstrated in our experimental model. For compound **9**, we have not found any neuroprotective or toxic effects for the concentrations tested. A detailed description of the activity of compounds **2**, **4**, **6**, **8**, and **9** is provided in the Supplementary Materials (Figure S29).

### 2.5. Effect of Individual Natural Products Isolated from the V. officinalis Roots on the Activation of Platelet αIIbβ3 Integrin

**Figure 9:**
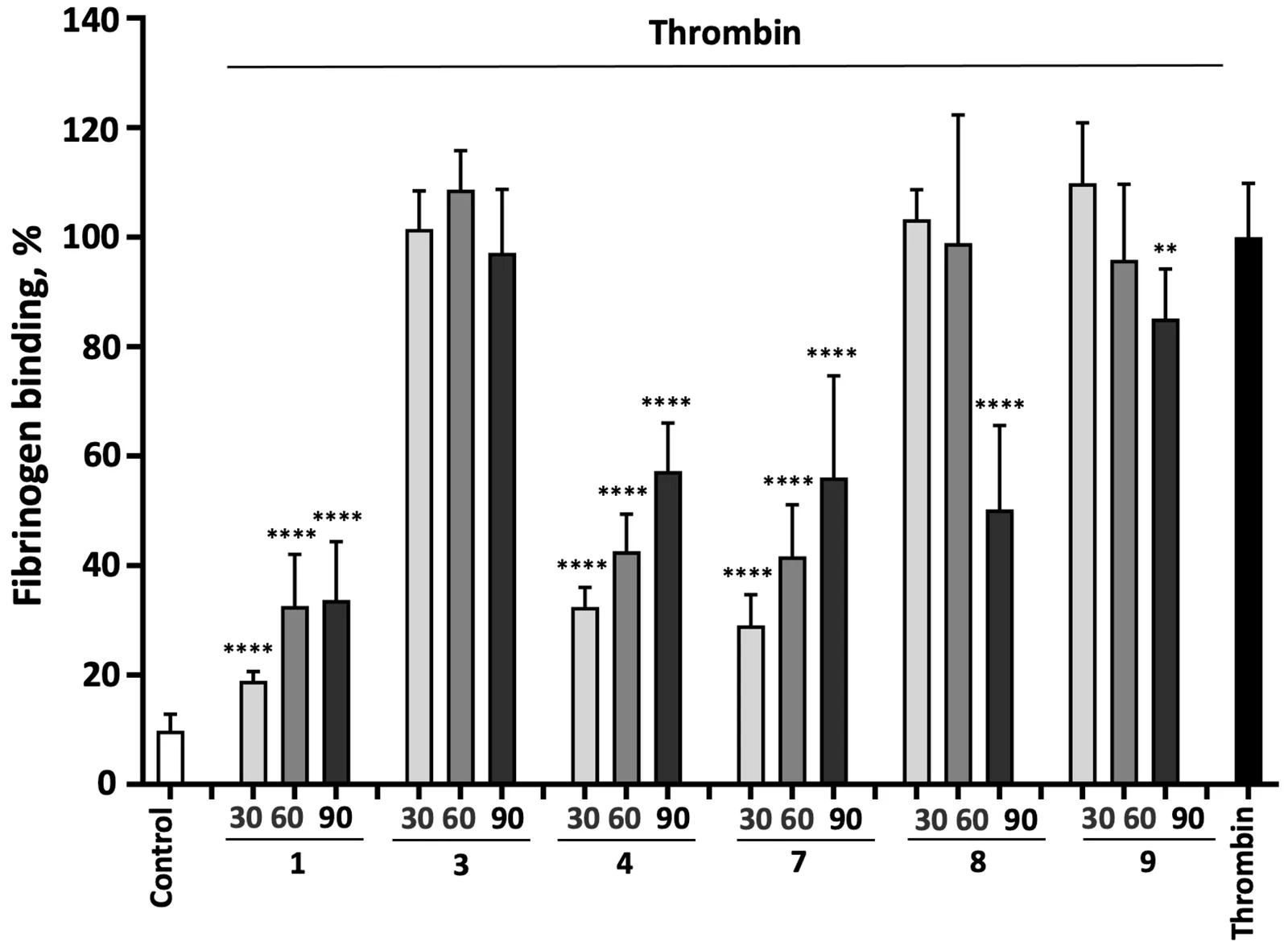
Compounds **1**, **4**, **7**, **8**, and **9** inhibited thrombin-induced platelet activation. Washed human platelets (0.5 × 10^8^/mL) were incubated with fibrinogen (15 *μ*g/mL) for 15 min, then the specified compounds (30, 60, and 90 *μ*mol/L) were added and activation was performed after 30 min by supplementation of thrombin aqueous solution (50 mU/mL) to all probes, excluding control. After 3 min, activation of platelets αIIbβ3 integrins was assessed with a Fibrinogen Alexa-647 binding by flow cytometry. The reaction was stopped by addition of PBS buffer (1:10). DMSO was added to the negative control and control with thrombin. Data are presented as mean ± SD. Fibrinogen binding in the control with thrombin was taken as 100%, *n* = 5, one-way ANOVA, Dunnett’s multiple comparison test ****, *p* < 0.0001, **, *p* < 0.01 compared to thrombin.

The effects of iridoids and valerenic acid derivatives isolated from the roots of *V. officinalis* on platelet activation were studied. To address this aspect, the effects of the target compounds on the platelet reactivity were assessed by their ability to activate integrin αIIbβ3. Our preliminary experiments showed that 30 min of incubation and test compound concentrations of 30 *μ*mol/L (low), 60 *μ*mol/L (medium), and 90 *μ*mol/L (high) have different effects on platelet activity. Washed platelets were incubated with compounds **1**–**9** for 30 min prior to stimulation with thrombin. Activation of αIIbβ3 integrin was followed by flow cytometry as dynamics of the binding of integrin to fibrinogen. Isovaltrate isovaleroyloxyisohydrin (**1**), iridoidvol A (**4**), and valerenic acid (**7**) strongly inhibited thrombin-induced platelet activation across the whole range of tested concentrations with maximal inhibitory effects observed at the concentration of 30 *μ*mol/L (18.9% ± 1.7, 32.4% ± 3.6, 29.1% ± 5.7, for compounds **1**, **4**, and **7**, respectively). Remarkably, hydroxyvalerenic acid (**8**) and acetoxyvalerenic acid (**9**) inhibited thrombin-induced platelet activation in a concentration-dependent manner. Thereby, the strongest inhibitory effect (50.2% ± 15.4 and 85.1% ± 9.1 for compounds **8** and **9**, respectively) could be observed at the concentrations of 90 *μ*mol/L (Figure 9). Among all the compounds tested, only 2-oxovalerenic acid (**3**) had no effect on thrombin-induced platelet activation.

**Figure 10:**
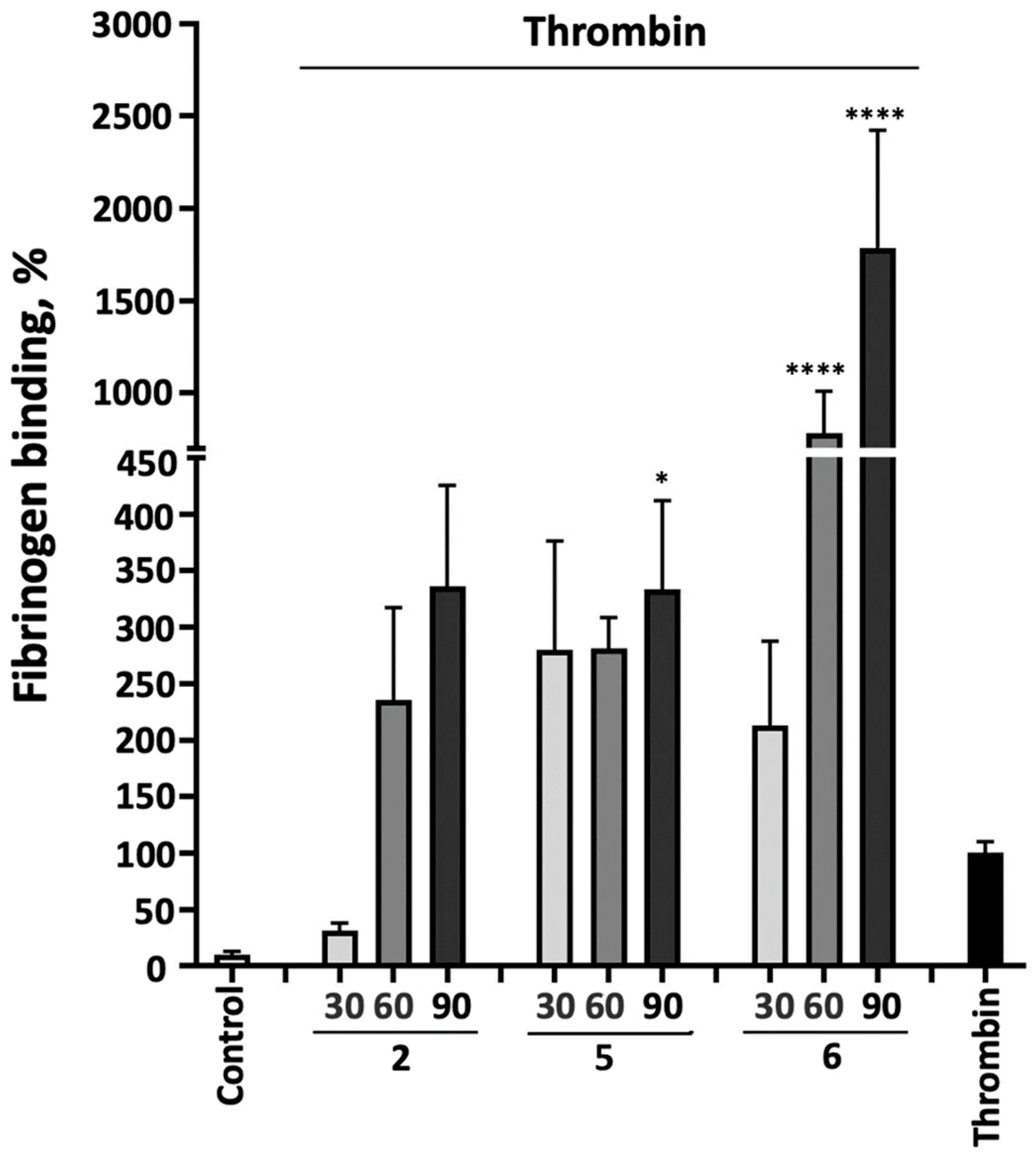
Compounds **2**, **5**, and **6** potentiated thrombin-induced platelet activation. Washed human platelets (0.5 × 10^8^/mL) were incubated with fibrinogen (15 *μ*g/mL) for 15 min, then the specified compounds (30, 60, and 90 *μ*mol/L) were added and activation was performed after 30 min by supplementation of thrombin aqueous solution (50 mU/mL) to all probes, excluding control. After 3 min, activation of platelet αIIbβ3 integrins was assessed with a Fibrinogen Alexa-647 binding by flow cytometry. The reaction was stopped by addition of PBS buffer (1:10). Corresponding concentration of DMSO was added to the negative control and control with thrombin. Data are represented as means ± SD. Fibrinogen binding in control with thrombin was taken as 100%, *n* = 5, one-way ANOVA, Dunnett’s multiple comparison test ****, *p* < 0.0001, *, *p* < 0.05 compared to thrombin.

Out of all tested compounds, only isovaltrate *α*-(isovaleroxy)isovaleroxyhydrin (**2**), isovaltrate isovaleroyloxyhydrin (**5**), and valtrate hydrine B1 (**6**) strongly potentiated thrombin-induced platelet activation (Figure 8). Compound **2**, taken even at a concentration of 30 *μ*mol/L, strongly potentiated thrombin-induced platelet activation (284.6 ± 79.2%), while increasing the concentration to 60 and 90 *μ*mol/L did not yield statistically significant enhancement of the effect (281.1% ± 27.4 and 333.1% ± 78.9 for 60 and 90 *μ*mol/L, respectively). On the other hand, compound **5** showed strong potentiation of the thrombin-induced platelet activation at the concentrations of 60 *μ*mol/L (235.2% ± 82.0) and 90 *μ*mol/L (335.9% ± 89.6). However, at a concentration of 30 *μ*mol/L, compound **5** acted as a potent inhibitor of platelet activation (31.2% ± 6.9). Finally, valtrate hydrine B1 (**6**) potentiated thrombin-induced platelet activation in a concentration-dependent manner. This effect was pronounced at all concentrations tested: 212.9% ± 74.4 at 30 *μ*mol/L, 885.7% ± 389.6 at 60 *μ*mol/L, and 1785.4% ± 638.3 at 90 *μ*mol/L (Figure 10).

### 2.6. Effect of the Individual Compounds Isolated from the Roots of V. officinalis on TRPV1 Ion Channels

**Figure 11:**
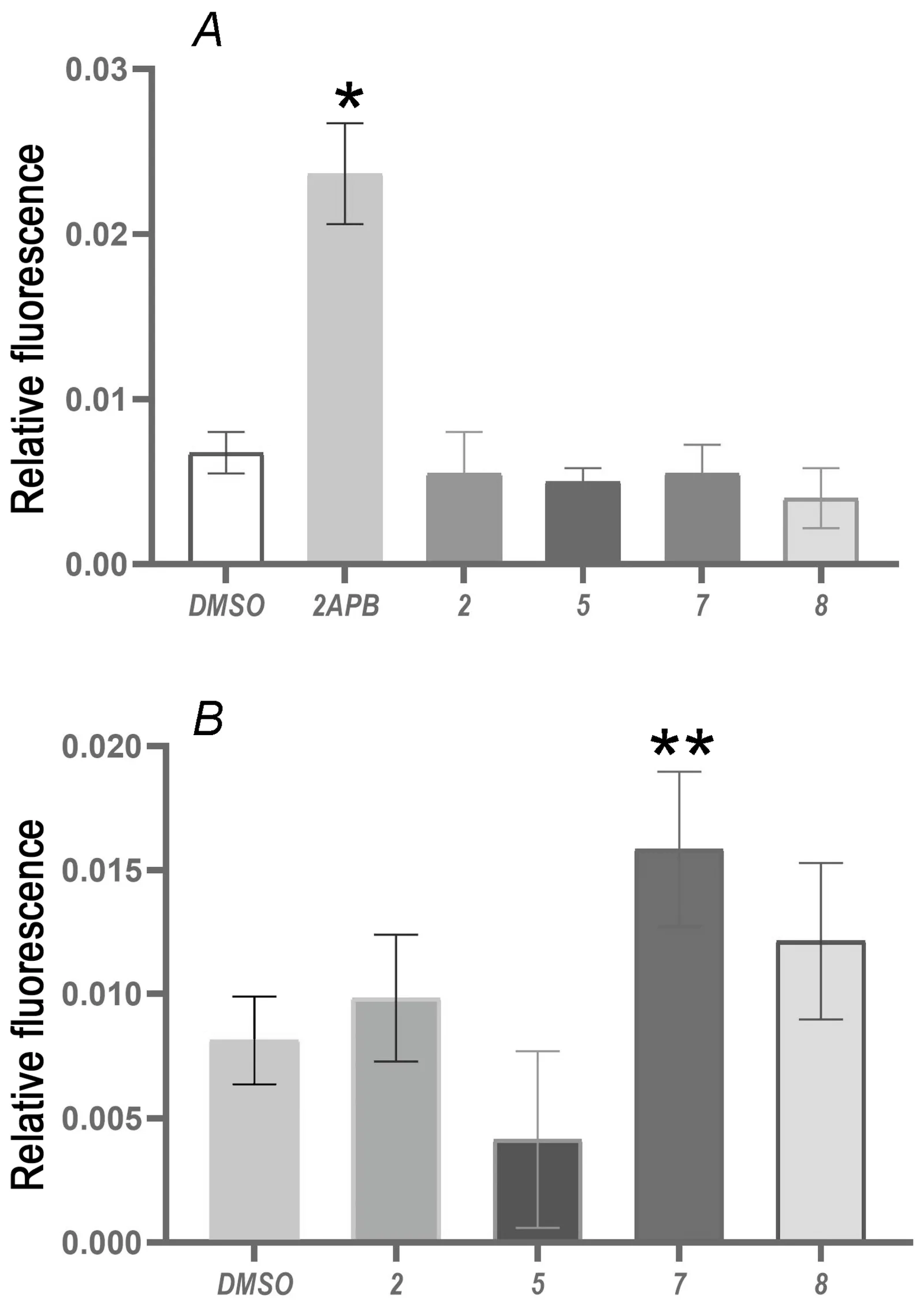
The effects of the compounds **2**, **5**, **7** and **8** on intracellular calcium levels. The experiments were accomplished with the CHO cell line expressing the rat TRPV1 ion channel. (**A**,**B**) Relative changes in the fluorescence response of the Fluo-4 dye recorded after application of the test compounds (5 *µ*mol/L) in the absence (**A**) or presence (**B**) of the agonist (300 *μ*mol/L 2APB). Data are represented as means ± SD, compared to control solution of DMSO diluted by PBS buffer, significant difference shown as * (*p* < 0.05), ** (*p* < 0.01), for ANOVA and Dunnett post-hoc test, *n* = 4.

The preliminary experiments accomplished with 0.1 mmol/L individual compounds applied to the CHO cells expressing non-selective Ca^2+^-permeable ion channels TRPV1 revealed a pronounced effect of compounds **2**, **5**, **7**, and **8** on the intracellular Ca^2+^ levels. Direct application of the isolated compounds **5** and **8** at the concentration of 5 *μ*mol/L caused a visible but nonsignificant decrease in the intracellular calcium concentration in comparison to the controls (DMSO solution). In contrast, compounds **2** and **7** applied at the concentration of 5 *μ*mol/L did not show any shifts in the calcium levels relative to the controls (Figure 11A). Co-application of the compounds together with the channel agonist 2APB did not significantly change the dye response for **2**, **5**, and **8**, but resulted in its significant increase when **7** was applied (Figure 11B). Thus, the potentiating effect on TRPV1 activation by 2APB appeared to be convincing only for **7**.

**Figure 12:**
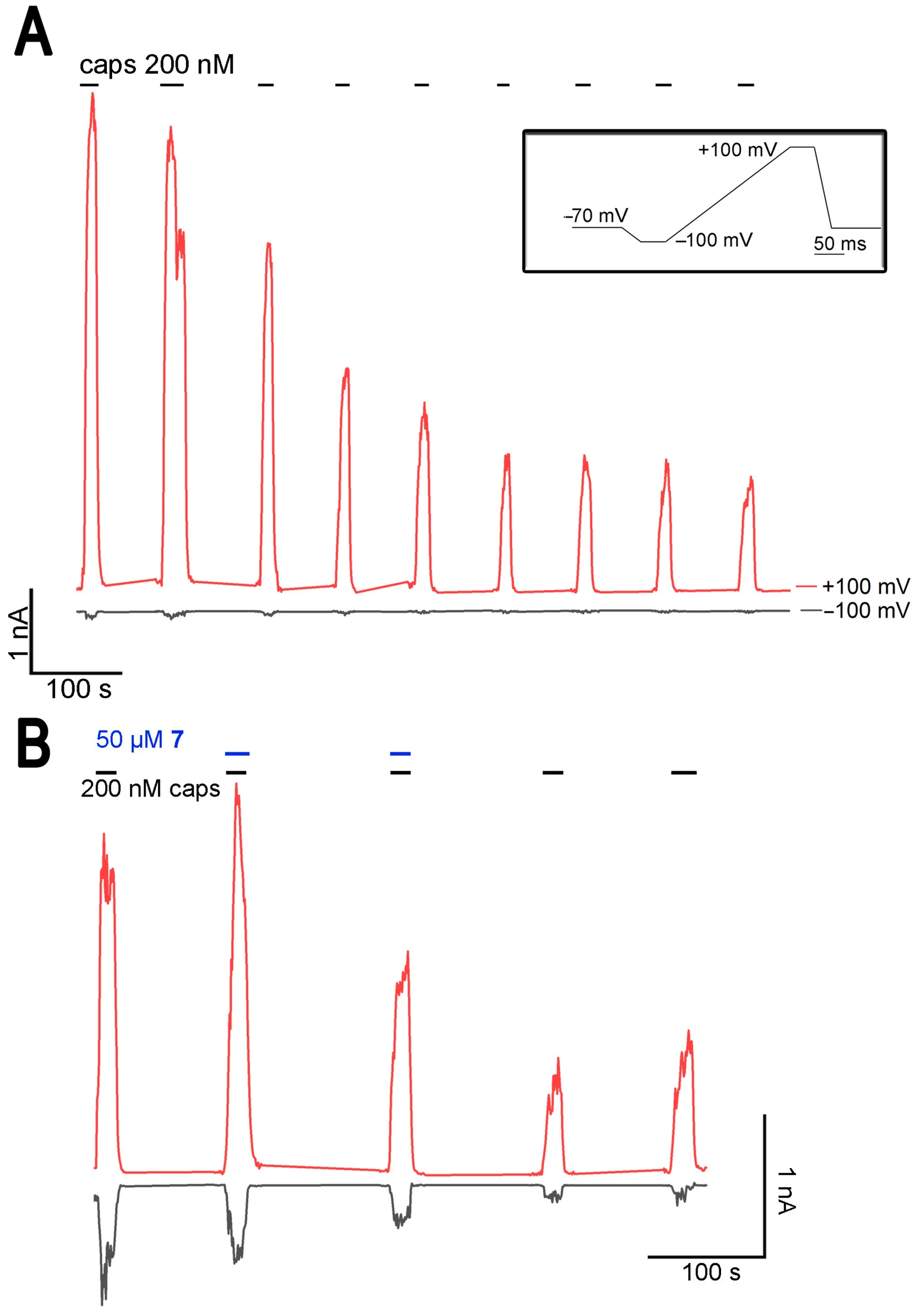
The effect of **7** on the membrane potential of CHO/rTRPV1 cells. (**A**) A representative trace of the cell’s response to 200 nmol/L capsaicin repetitive application, measured at ramp condition (shown in the insert). (**B**) A representative trace of an individual cell, acquired when 200 nmol/L capsaicin was applied in the presence and absence of 50 *μ*mol/L of the compound **7**. The data follow a Gaussian distribution. The statistical difference was not significant (*t*-test, *p* < 0.05).

To address the mode of action of the compound **7** on the TRPV1 ion channel, the same cell model was employed for the assessment of the currents across the cell membrane. For this, the electrophysiological approach in the whole-cell configuration at voltage clamp was used. In terms of this approach, a stable run-down effect at the voltage ramp protocol was measured after repeated application of 200 nmol/L capsaicin (a selective agonist of the TRPV1 channel, Figure 12A). The co-application of 50 *μ*mol/L of compound **7** and capsaicin led to an increase in the current during the first application, followed by a decrease in the current during the second application and in a delay of the compound washout from the cell (Figure 12B). Thus, the compound **7**, at least partially, acted as a modulator of the TRPV1 ion channel with a negative regulating effect on TRPV1 through a yet unknown mechanism.

## 3. Discussion

To date, classical phytochemistry is universally recognized as the tool in the search for plant secondary metabolites with pronounced pharmacological effects to create new innovative drugs. Therefore, despite the fact that both shoots and roots of valerian are already comprehensively studied in respect of their phytochemical composition and characteristic biological activities [28,29,30], the interest to the search of previously undescribed constituents of *V. officinalis* extracts and associated pharmacological effect is still growing. The aim of the study was to establish the activity profile of individual compounds, as well as to determine preliminary mechanisms of their action and possible side effects of use. Here, comprehensive RP-HPLC analysis of ethanolic extracts obtained from the *V. officinalis* roots and isolation of the major components from these samples were accomplished. Based on the relative intensities of the signals at the UV chromatograms acquired at 254 nm of the dichloromethane fraction of the extract, we isolated nine major compounds (intensity of the signals more than 50.0 mAU on the HPLC-UV chromatogram). Thereby, three of these natural products (valepotriates **1** and **2**, as well as the valerenic acid derivative **3**) appeared to be previously undescribed, i.e., their structures assigned on the basis of the acquired here spectral information (1D and 2D NMR spectroscopy) are reported here for the first time. Besides, six further major signals could be assigned to the secondary compounds which were characterized in *V. officinalis* extracts earlier.

The group of valepotriates comprises iridoid compounds, which are the main group of *V. officinalis* compounds. So far, about 20 compounds from this group (mostly differing by their esterification patterns) are described [13]. Among them, three compounds, namely iridoidvol A (**4**), isovaltrate isovaleroyloxyhydrin (**5**), and valtrate hydrine B1 (**6**), belonged to the major constituents of the roots extracts and were, therefore, isolated and their structure was unambiguously assigned by NMR spectroscopy and multi-staged high-resolution tandem mass spectrometry (HR-MS/MS) to the earlier identified structures [31,32]. Among the derivatives of valerenic acid, only valerenic acid itself, hydroxyvalerenic acid and acetoxyvalerenic acid have been isolated from natural sources [13,33]. Further, all three derivatives of valerenic acid described in literature so far (valerenic acid itself, hydroxyvalerenic acid and acetoxyvalerenic acid) [13,33] were found as the major compounds in the *V. officinalis* extracts here (compounds **7**–**9**). Now we extend the list of the known valerenic acid derivatives, as a previously undescribed compound—2-oxovalerenic acid (**3**) was successfully isolated from our valerian extracts and its structure could be unambiguously assigned.

The study of the activity profile of isolated compounds with subsequent establishment of their mechanisms of action and possible side effects of their use are key requirements of evidence-based medicine for assessing the efficacy and safety of drugs. In this regard, a comparison of the activity of the plant extract and the pure compound in this case does not seem necessary, since, firstly, such a study requires additional studies of the content of the isolated components and a revision of the recommendations for the clinical use of extracts based on the data obtained, which was not the purpose of this study, and secondly, it does not allow us to accurately determine the contribution of each component and understand their individual properties. As valerian extracts and many of their individual constituents are known for strong protective effects on the nerve system [34], and mechanistically these effects are at least to a great extent underpinned by their antioxidant properties [35], we addressed this issue in more detail here. For this, we characterized the antioxidant potential of the individually isolated compounds with a DPPH method, which is conventionally used to probe antiradical activities of natural products [36]. The tests revealed relatively low levels of antioxidant activity with a pronounced dose-dependent effect. The only exception was compound **3**, the activity of which, however, did not show any dose-dependency. Among the others, compounds **1**, **4**, and **6** from the group of valepotriates showed the highest activity at the concentration of 100 μmol/L (normalized antioxidant activity by ascorbic acid was as follows 15–17%), which was probably due to better accessibility of the mobile hydrogen atom in their structure (that was not the case for compounds **2** and **5**). Valerenic acid derivatives, in turn, demonstrated lower antioxidant activity, probably due to the formation of an intramolecular hydrogen bond that reduced the mobility of the hydrogen atom in the carboxyl group. Thus, in this group, compounds **3** and **9** had the highest activity (normalized antioxidant activity by ascorbic acid was as follows 6% and 7%, respectively) when applied at the concentration of 100 μmol/L. The activity of compounds **7** and **8** was minimal among all the studied compounds—normalized antioxidant activity by ascorbic acid was as follows 3% and 2.5%, respectively.

The extracts of valerian have a long history of successful application in medicine as a sedative and hypnotic remedy. Its mechanism of action was supposed to involve GABA signaling affecting the GABAA receptors, which decreases neuronal network excitability [37]. This mechanism leads to the idea that valerian extracts might be neuroprotective. This assumption was comprehensively confirmed over the two recent decades—it was shown that the neuroprotective effects of the valerian extracts are underlined by modulation of the calcium signaling and antioxidant properties [34,38,39].

Here we make a step forward and address the neuroprotective effects of individual constituents of the valerian extracts that might be the strongest contributors in the extract properties. We found that two derivatives of valerenic acid (2-oxovalerenic acid and hydroxyvalerenic acid) preliminary showed neuroprotective effects in the selected model and might act, therefore, as such contributors. Such properties were not shown for these compounds before, although the neuroprotective activity of valerenic acid as the main component of valerian extract was investigated in the mouse model of *D*-galactose induced aging [40]. The mechanism of its neuroprotective effect was proposed to act through the BDNF-mediated regulation of the GABA_A_ receptor expression [41]. Besides, valerenic acid was found to reduce neuroinflammation and thus ameliorate neurodegeneration in the rodent model of Parkinson disease [42], which most likely relies on suppression of the NF-kB activation [43]. This compound was shown to improve behavioral outcomes and plasma A*β*1–42 levels in the rodent model of Alzheimer disease [44].

In our study, 2-oxovalerenic acid was found to exert the neuroprotective effect over a broad range of concentrations and did not show any signs of toxicity even at the highest concentration tested. To the best of our knowledge, the neuroprotective activity of these compounds was not shown so far. Thus, the underlying molecular mechanisms can be addressed in the future works. Despite the generally accepted concept of the involvement of oxidative stress in the development of neuronal disorders, in our study there was no correlation between antioxidant and neuroprotective potential of isolated compounds. Therefore, we can make a preliminary conclusion about the specific neuroprotective effect of compound **3** probably related to the effect on internal receptor and neurotransmitter systems. However, to reveal the exact mechanism of neuroprotective effect of individual components of valerian extract, it is necessary to carry out additional studies.

As mentioned above, valerian extracts may show effects on the signaling of individual neurotransmitters through receptor interactions. Therefore, it may be of interest to investigate the effects of isolated metabolites on individual receptor systems. Valerenic acid has so far been characterized as a ligand to a single type of ion channels. This channel is a GABA receptor containing β2/3 subunits, but not β1 subunit [37,45]. The activities on this target were well supported by the effect of valerian extracts on alleviating restlessness and anxiety at in vivo experiments on wild-type mice in the elevated plus maze and the light/dark choice test [17]. The activity exhibited by valerenic acid to TRPV1 ion channel potentiating and activation is comparable in concentration to a potentiating effect observed on GABA_A_ α1β2γ2 (EC50 18.9 *μ*mol/L at 838%) and on GABA_A_ α1β3 (EC50 22.8 *μ*mol/L at 858%) [45]. Thus, the effect described in this study should not be viewed as a side effect of valerenic acid; rather, this discovery points to a novel, important molecular target for the compound. The study data indicate that valerenic acid likely possesses affinity for TRPV1 and may act as a modulator of the TRPV1 channel. However, further research is required to elucidate the precise mechanism of interaction.

The use of valerenic acid as an antinociceptive drug was proposed by Vidal-Cantú et al., 2016 [46] who considered this molecule as a partial agonist of the 5-HT5A metabotropic receptor [46]. Valerenic acid administered intrathecally and intraplantarly was successful as an analgesic in the formalin test at nanomolar concentrations, and its effect was not completely blocked by the non-selective 5-HT5A/5B antagonist methiothepin, or the selective 5-HT5A antagonist SB-699551 [46]. The discovery of valerenic acid’s effect on the TRPV1 receptor strongly suggests that the observed analgesic effect was partly due to the modulation of this ion channel. Moderate activation of this channel appears to lead to subsequent receptor desensitization, resulting in a loss of conductivity in TRPV1-expressing afferent neurons. The use of capsaicin as an analgesic drug is based on this principle, and the analgesic potential of capsaicin desensitization has been confirmed in clinical studies [47,48].

Many natural products as extracts and their isolated constituents are well-known modulators of platelet function [49]. Recently we showed that curcumin [50], C-glycoside Isoembinin isolated from Iris lacteal [51], *Bracteatinine* and isogroenlandicine, isoquinoline alkaloids isolated from *Corydalis bracteate* [52], flavonoid aglycones [53] are strong inhibitors of platelets by activation of diverse intracellular mechanisms. However, we did not find any solid data concerning effects of valeriana extracts, or isolated major compounds from *V. officinalis* on platelet function. Similarly, we found very little information concerning effects of iridoids on platelets. Some iridoids, including amarogentin, geniposide and genipin gentiobioside isolated from *Fructus gardeniae* significantly inhibited collagen-induced platelet activation partly through the inhibition of PLC γ2-PKC cascade and MAPK pathway [53]. Because iridoids belong to the major constituents of the *V. officinalis* extracts it was important to address the effects of the individual isolated compounds, on platelet activity. Unexpectedly, we found dual effects of isolated compounds on platelet activity. Compounds **1**, **4**, and **7** strongly inhibited thrombin-induced platelet activation. Moreover, inhibitory effects were more pronounced at low (30 *µ*mol/L) concentrations. Compounds **3** and **9** had no significant effect and compound **8** inhibited platelets only at high (90 *µ*mol/L) concentration. At the same time, compounds **2**, **5**, and **6** strongly potentiated thrombin-induced platelet activation. The presented data indicate that total valeriana extracts, because of its dual (activatory/inhibitory, that compensate each other) effect could have no influence on platelet reactivity but individual compounds (**1**, **4**, and **7**) might be promising prospective agents for adjuvant therapy of pathologies associated with increased platelet aggregation, such as cardiovascular diseases, hypertension, diabetes mellitus, cancer, etc. [54,55,56].

On the other hand, compounds **2**, **5**, and **6** demonstrated clear potentiating effect on the thrombin-induced platelet activation. Natural products with such properties can be considered as promising prototypes in development of new drugs and food supplements for the therapy of conditions accompanied by a decrease in platelet aggregation. The use of herbal teas and medicinal preparations based on isolates from such plants in medical practice appears to be promising. Indeed, extracts of the shoots of Urtica dioica and Achillea millefolium, rich in vitamin K [57,58] and the roots of *Sanguisorba officinalis*, *Bergenia crassifolia* or *Viburnum opulus*, rich in tannins and polysaccharides [59,60,61] were confirmed to be efficient in potentiating of the platelet activation. Thus, search of plant-derived natural products featured with such activities needs to be continued. Obviously, establishment of new formulations based on individual isolated metabolites with proven activity will expand the range of drugs for treatment of bleedings of various genesis. On the other hand, our data might indicate, that the use of valerian extracts with high contents of compounds **2**, **5**, and **6** in the therapy of other pathologies may cause undesirable adverse reactions such as increased blood clotting and thrombosis. However, in general, taking into account the multidirectional effects of individual metabolites observed in this model, their mutual antagonistic influence can be assumed. This might result in the fact that the valerian extract would not have a pronounced effect on the process of thrombosis in the body. However, obviously, this issue needs to be addressed in further comprehensive in vivo study.

## 4. Materials and Methods

### 4.1. Plant Material

This study relied on the roots of *V. officinalis* cultivated on the basis of the Russian manufacturer Krasnogorskleksreda (Russia, 143444, Moscow region, Krasnogorsk, Opalikha microdistrict, Mira str., 25). The roots were harvested in early spring 2023. Dr. Stanislav Sukhih, Immanuel Kant Baltic Federal University authenticated the original plant. A voucher specimen (BFU_2023_VO) was deposited at the Institute of Living Systems, Immanuel Kant Baltic Federal University. The collected material was dried in a desiccator at 50 °C. The dried material was grinded with a cutting mill (DM-6, HT Machinery, New Taipei City, Taiwan) and the grinded dry mass was passed through a sieve with 1 sm pore diameter and stored until the extraction in sealed paper bags at room temperature.

### 4.2. Materials

Unless stated otherwise, materials were obtained from the following manufacturers: Duchefa Biochemie (Haarlem, The Netherlands): dimethylsulfoxide (DMSO, >99.9 atom% D); Ecos-1 (Moscow, Russia): methylene chloride (analytical grade); Honeywell Riedel de Haën™ (Seelze, Germany): acetonitrile (>99.9%, LC-MS grade), methanol (LC-MS grade); J.T. Baker (Radnor, PA, USA): acetonitrile (>99.9%, HPLC gradient grade); Roche (Mannheim, Germany): thrombin; Molecular probes (Gottingen, Germany): Fibrinogen Alexa-647. All other chemicals were purchased from Merck KGaA (Darmstadt, Germany). Water was purified in house with a water conditioning and purification system GenPure Pro (resistance 18 mΩ/cm, Thermo Fisher Scientific, Langenselbold, Germany).

### 4.3. Extraction, Fractionation, Isolation and Structure Elucidation

Dried roots of *V. officinalis* L. (700 g) were subjected to tenfold extraction with 96% ethanol at a ratio of 1:10 (7 L of ethanol for each extraction procedure) at room temperature (RT). The obtained extracts were combined, and the solvent was partly evacuated to 500 mL under reduced pressure in a rotary evaporator (DLAB SCIENTIFIC Co., Ltd., Beijing, China). The crude extract was exhaustively re-extracted by liquid-liquid partition via dichloromethane (500 mL) using a glass separation funnel. The dichloromethane fraction was evaporated to dryness and resuspended in 50 mL of 96% (*v*/*v*) aq. ethanol. The individual compounds were isolated from the dichloromethane fraction using a procedure described previously [62] with some changes. In detail, the dichloromethane fraction was separated by chromatography on Sephadex LH-20 column and isocratic elution with 100% EtOH. Two resulting fractions were further separated by preparative reversed-phase high performance liquid chromatography (RP-HPLC) with UV-detection at 254 nm. The concentrated samples (8 mL) were injected in a Knauer Smartline system (Knauer Wissenschaftliche Geräte GmbH, Berlin, Germany) and separated at 40.0 mL/min on a Kromasil C18 preparative HPLC column (250 × 30 mm, 5 *μ*m, Nouryon, Amsterdam, The Netherlands) thermostated at 40 °C. The separation relied on the linear gradient elution mode with 0.1% (*v*/*v*) aq. trifluoroacetic acid (TFA) and acetonitrile as eluents A and B, respectively. After a five-min wash-out the unbound fraction at 5% eluent B, the samples were separated in a linear gradient to 95% eluent B in 50 min. Afterwards, the column was washed with 95% eluent B for 5 min and re-equilibrated at 5% eluent B for 5 min. After isolation, individual compounds were freeze-dried and stored at −20 °C.

### 4.4. Nuclear Magnetic Resonance Spectrometry (NMR) Experiments

The 1H-, 13C NMR and 2D-NMR spectra of the isolated natural compounds were recorded in DMSO-d6 (Merck KGaA, Darmstadt, Germany) on a Bruker Avance III 400 NMR Spectrometer (Bruker, Bremen, Germany) equipped with a 3 mm broadband probe (1H, 31P, 13C, 15N) and with cooled 1H and 13C pre-amplifiers (1H at 400 MHz and 13C at 100 MHz). Acquisition parameters included a 45 hard pulse angle, a sweep width of 14 ppm, 3.27 s acquisition time, 1 s relaxation pulse delay. Up to 2000 scans were collected per sample, corresponding to ~1 h of collection time.

### 4.5. Tandem Mass Spectrometry (MS^n^) Experiments

The structural identity of the individually isolated metabolites was confirmed by a series of targeted tandem mass spectrometry experiments. For chromatographic separation Waters^®^ ACQUITY UPLC^®^ I-Class System, equipped with a Binary Solvent Manager, FTN Sample Manager and PDA eλ Detector (Waters GmbH, Eschborn, Germany) was used. The eluents A and B were ultrapure water and acetonitrile with 0.1% (*v*/*v*) formic acid. The gradient profile for eluent B was as follows: 0.0–1 min 5%; 1–11 min increase to 95%; 11–13 min hold at 95%; 13–13.5 min decrease to 5%. For re-equilibration of the UHPLC column, the gradient was set to 3% eluent B for 3.1 min. The column oven was set at 55 °C and the autosampler was cooled at 8 °C. The flow rate was kept constant at 0.4 mL/min. A volume of 2 *μ*L of the sample was injected onto a BEH RP-C18 column (50 mm × 2.1 mm i.d.; 1.7 *μ*m particle size, 100 Å), guarded with a C18 guard column (2.1 mm i.d. × 5.0 mm; BEH C18; Waters, both Waters GmbH, Eschborn, Germany). The wavelength range of the PDA measurements was set to 190–600 nm.

Mass spectrometric detection in positive and negative ion mode relied on a QqTOF-MS instrument equipped with an ESI-DuoSpray-Ion-Source equipped with a stainless-steel electrode (100 *μ*m internal diameter), operated in positive and negative ion mode and controlled by Analyst TF 1.7.1 Software (TripleTOF 6600, Sciex, Darmstadt, Germany). Mass calibration was accomplished externally every eight injections. The ion source was operated under the following ionization conditions: nebulizing gas (gas 1), nitrogen (90 psi); drying gas (gas 2), nitrogen (70 psi); ion spray voltage, 4500 V; ion-source temperature, 450 °C; curtain gas, nitrogen (35 psi). The mass spectra were acquired with accumulation time set to 50 ms over the mass range of *m*/*z* 50 to 1250. In the positive ion mode, the tandem mass spectra (MS/MS) were acquired in the high sensitivity mode with the accumulation time set to 50 ms, collision energy (CE) in the range 20–82.5 V and declustering potential (DP) of 35 V. In the negative ion mode CE range was −20–−82.5 V and DP was set to −35 V. Data evaluation relied on the PeakView Software Version 2.2 (Sciex, Marlborough, MA, USA).

### 4.6. Assessment of Anti-Neurodegenerative Effects

Neuroprotective activity of *Valeriana officinalis* L. individual compounds was tested on the model of prevention of A*β*25–35 (synthesis of A*β*25–35 is presented in Supplementary Material) toxicity for differentiated SH-SY5Y cell culture. The SH-SY5Y (ECACC, Porton Down, GB) cell culture was maintained in DMEM/F-12 culture media (Capricorn Scientific, Ebsdorfergrund, Germany) supplemented with 10% FBS (Thermo Scientific, Waltham, MA, USA) and non-essential amino acid solution (NEAA, Capricorn Scientific, Ebsdorfergrund, Germany) diluted as 1:100 according to manufacturer’s instruction. The cells were grown at 37 °C and 5% CO_2_ concentration in Binder C150 incubator (Binder, Tuttlingen, Germany). For seeding the cells were detached from plastic by two PBS washes and incubation in Trypsin-EDTA solution (Biolot, St. Petersburg, Russia) for 1 min, then collected by centrifugation at 800 g for 5 min, and counted using Neubauer chamber. For differentiation of the SH-SY5Y cells were maintained for 8 days in the DMEM/F-12 media supplemented with 3% FBS and 10 *μ*mol/L retinoic acid (Sigma Aldrich, Burlington, MA, USA).

For the neuroprotective effect assessment, the differentiated cells were seeded at 0.4 million per mL density onto 96-well tissue culture treated plates in the differentiation media. On the next day the valeriana extract individual components diluted in DMEM/F-12 media were applied to the seeded cells together with 25 *μ*mol/L of A*β*25–35 (leaving the corresponding controls of cells without A*β*25–35, cells without extract and untreated cells). After 24 h incubation 0.5 mg/mL MTT (Sigma Aldrich, Burlington, MA, USA) solution was added to the cells and incubated for 5 h for formazan crystal formation. After the incubation the crystals were dissolved in 3% (*w*/*v*) SDS solution overnight. The intensity of purple coloration of formazan was measured with ClarioStar plate reader (BMG LabTech, Ortenberg, Germany) at the two wavelengths—570 nm and 630 nm. Cell viabilities were counted by subtraction of absorbance at 630 nm from the absorbance at 570 nm and the subsequent subtraction of the signal from the blank wells.

### 4.7. Human Platelet Activation Experiments

#### 4.7.1. Human Platelet Experiment

Human platelets were prepared as previously reported with minor modifications [63]. Blood was obtained from healthy volunteers according to our institutional guidelines and the Declaration of Helsinki. All experimental protocols were submitted and approved by the Ethical Committee of Sechenov Institute of Evolutionary Physiology and Biochemistry of the Russia Academy of Sciences (protocol no. 1–04 form 7 April 2022). Blood from healthy volunteers after their written informed consent was collected into ACD solution (12 mmol/L citric acid, 15 mmol/L sodium citrate, 10 mmol/L D-glucose plus EGTA 2 mmol/L final concentration). The whole blood was centrifuged at 1300 rpm for 8 min at room temperature (RT) to get platelet-rich plasma (PRP). PRP was diluted 1:1 with CGS buffer (120 mmol/L sodium chloride, 12.9 mmol/L trisodium citrate, 10 mmol/L D-glucose, pH 6.5) and centrifuged at 2500 rpm for 5 min, then the platelet pellet was resuspended in GCS buffer, centrifuged at 2500 rpm for 5 min and the platelet pellet was resuspended in HEPES buffer (150 mmol/L sodium chloride, 5 mmol/L potassium chloride, 1 mmol/L magnesium chloride, 1 mmol/L calcium chloride, 10 mmol/L D-glucose, 10 mmol/L HEPES, pH 7.4). After a 15 min rest in a 37 °C water bath, washed platelets (WP) were used for experiments. All isolated compounds were dissolved in DMSO, therefore in all experiments corresponding concentrations of DMSO were added to the negative control and positive control with thrombin.

#### 4.7.2. Flow Cytometry Analysis

Flow cytometry analysis was performed on a CytoFlex (Beckman-Coulter Inc., Brea, CA, USA). WP were taken at a concentration of 0.5 × 10^8^ platelet/mL. Each sample gained 10,000 events. The data were analyzed by CytExpert Acquisition and Analysis Software Version 2.4 (Beckman Coulter Inc., Brea, CA, USA).

#### 4.7.3. Measurement of Activated αIIbβ3 Integrin

For analysis of activated αIIbβ3 integrin WP were incubated with 15 *µ*g/mL of Fibrinogen-Alexa-Flour-647 together with indicated concentrations of teased compounds or appropriate concentration of DMSO, at 37 °C for 30 min. Then platelets were stimulated by thrombin (50 mU/mL) for additional 3 min, after which the reaction was stopped through dilution by phosphate buffer (PBS) (1:10) for subsequent flow cytometry analysis.

### 4.8. Antioxidant Assay

The antioxidant effect of the isolated individual compounds was addressed by 2,2-diphenyl-1-picrylhydrazyl (DPPH) free radical scavenging assays. It was done according to Masci et al. (2015) [36] with minor modifications as follows.

Isolated individual compounds were solubilized in DMSO at the concentration of 50 mg/mL (stock solution). The aliquots of each sample (concentration range for the optimization experiment from 10 *μ*mol/L to 100 *μ*mol/L; final concentration for the experiment 50 *μ*mol/L, 75 *μ*mol/L, and 100 *μ*mol/L) were supplemented to 1 mL portions of 40 μmol/L methanolic solution of stable nitrogen centered free radical DPPH^•^. The absorbance was monitored photometrically at 517 nm after 1 h incubation at RT. The capacity of the samples for scavenging of the DPPH^•^ radical was estimated from the difference in the absorbance acquired in the presence and in the absence of individual compound. The corresponding values were expressed as the percentage of DPPH^•^ consumption as a function of the sample concentration. Thereby, the oxidant activities of the different concentration of individual compounds were determined as relative values normalized to the antioxidant activity of 40 *μ*mol/L ascorbic acid (taken as 100%) [64].

### 4.9. Measure of Modulating Effect on TRPV1 Ion Channels

Chinese hamster ovary (CHO) cell line stably expressing rat TRPV1 was obtained as described previously [65,66]. CHO/rTRPV1 cells were cultivated in DMEM/F12 (1:1) (ThermoFisher Scientific Inc., Waltham, MA, USA) with 10% fetal bovine serum (HyClone, Logan, UT, USA), 100 U/mL penicillin, 100 µg/mL streptomycin at 37 °C and 5% CO_2_. For intracellular calcium assay CHO/rTRPV1 were seeded into black-walled, clear-bottomed 384-well plates at a density of 20.000 cells per well (complete media without antibiotics but containing 1 *µ*g/mL tetracycline to induce TRPV1 channel expression) and were cultured overnight at 37 °C, 5% CO_2_. For analysis cells were loaded with the cytoplasmic calcium indicator Fluo-4AM using Fluo-4 Direct™ Calcium Assay Kit, and Hoechst 33342 fluorescent dye for DNA staining (both from ThermoFisher Scientific Inc., Waltham, MA, USA) mixed in media of cultivation at 37 °C for 60 min. Then cells additionally were incubated in the dark at 25 °C for 30 min for Fluo-4AM conversion and the fluorescence was measured on cell imager system Spark^®^ Cyto (Tecan, Männedorf, Switzerland). Data were collected in the blue channel for Hoechst 33342 dye for cell counting and in the green channel for Fluo-4 intracellular calcium signal. Spark Control and Acquisition software Version 3.1 was used to calculate the average cell fluorescence. Alternatively, the cells images were collected using a Leica sCMOS microscope camera mounted on Leica DM IL LED Fluo microscope and analyzed in ImageJ 1.54g. TRPV1 activation was induced by adding 200 *μ*mol/L solution of the agonist 2-[(Diphenylboranyl)oxy]ethane-1-amine (2APB).

### 4.10. Patch-Clamp Electrophysiology

Whole-cell patch-clamp currents were recorded using EPC-800 amplifier (HEKA, Reutlingen, Germany), InstruTECH LIH 8 + 8 data acquisition system (HEKA), and PatchMaster v2x90.5 software (HEKA). Extracellular solution contained (in mmol/L) 140 NaCl, 5 KCl, 2 CaCl_2_, 1 MgCl_2_, 10 *D*-Glucose, 10 4-(2-hydroxyethyl)-1-piperazineethanesulfonic acid (HEPES), pH 7.4 adjusted with 1 mol/L NaOH. Patch pipettes were pulled from borosilicate glass with filament with a P-1000 flaming/brown micropipette puller (Sutter instrument, Novato, CA, USA). The internal solution contained (in mmol/L) 135 CsCl, 5 NaCl, 1 MgCl_2_, 10 EGTA, 10 HEPES, 10 *D*-Glucose. The pipette resistance ranged from 3 to 5 MOm when filled with the internal solution. The holding potential was −70 mV. Whole cell recording was performed at 1.25 Hz frequency under voltage ramps from −100 to +100 mV (200 ms). A single ramp recording was registered at 0.5 kHz and low-pass filtered at 3 kHz. Activation of the current through TRPV1 was carried out with 200 nmol/L solution of the specific agonist capsaicin using the SF-77B Perfusion Fast Step flow system (Warner Instruments, Holliston, MA, USA).

### 4.11. Statistical Analysis

Antioxidant activity was analyzed using the DPPH radical-scavenging assay in three independent technical replicates; the statistical significance of differences between groups was assessed using one-way ANOVA (*p* ≤ 0.05) followed by Tukey’s post-hoc test. Neuroprotective activity against A*β*25–35-induced toxicity in differentiated SH-SY5Y cells, as well as the modulatory effect on TRPV1 ion channels, were analyzed in four independent biological replicates; the statistical significance of differences between groups was assessed using one-way ANOVA (*p* ≤ 0.05) followed by Dunnett’s post-hoc test. The ability of the compounds to affect thrombin-induced platelet activation was analyzed using samples from five independent donors; the statistical significance of differences between groups was assessed using one-way ANOVA (*p* ≤ 0.05) followed by Dunnett’s post-hoc test. Data were presented as means ± SD. GraphPad Prism 8 (GraphPad Software, San Diego, CA, USA) was used for data analysis.

## 5. Conclusions

Five iridoids, including two previously undescribed natural products—isovaltrate isovaleroyloxyisohydrin (**1**) and isovaltrate *α*-(isovaleroxy)isovaleroxyhydrin (**2**), together with four valerenic acid derivatives, including one previously undescribed metabolite—2-oxovalerenic acid (**3**) were isolated from roots of *Valeriana officinalis* L. All isolated compounds, with the exception of 2-oxovalerenic acid (**3**), strongly affected thrombin-induced platelet activation. Thereby, in respect of thrombin-induced platelet aggregation, these metabolites showed a clearly diverse activity pattern, i.e., isovaltrate isovaleroyloxyisohydrin (compound **1**), iridoidvol A (compound **4**), and valerenic acid (compound **7**) demonstrated an inhibitory effect on thrombin-induced platelet activation, whereas isovaltrate *α*-(isovaleroxy)isovaleroxyhydrin (compound **2**), isovaltrate isovaleroyloxyhydrin (compound **5**), and valtrate hydrine B1 (compound **6**) showed a potentiating effect in the same experimental model. Moreover, it can be preliminary stated that compound 3 exhibited a neuroprotective effect in a model assessing the prevention of A*β*25–35 toxicity in differentiated SH-SY5Y cell cultures. Valerenic acid showed activity on TRPV1 ion channels, manifested by a potentiation of the channel’s response to the capsaicin/2APB in cells followed by a decrease in channel activity. The study of antioxidant properties showed dose-dependent but low indices of antiradical activity of the isolated compounds, which probably indicates a specific mechanism of neuroprotective action of isolated compounds that requires additional study.

The obtained data on the isolation of previously uncharacterized natural products, as well as the establishment of the biological activity profile of individual compounds, allow for the assessment of the contribution of individual metabolites to the complex effect of popular drugs based on extracts of *V. officinalis*. On the other hand, the study of the activity profile of isolated compounds might give better insight in the pharmacological potential of *V. officinalis* from side of evidence-based medicine. This approach is currently the state of the art in the practical application of biologically active natural products and allows answering the questions about the efficiency and safety of the plant extracts.

In this study, the efficiency of several valerian metabolites was confirmed in terms of the neuroprotective properties of *V. officinalis*. The newly discovered properties allow considering this plant species as a source of new potential agents for the development of new drugs and biologically active additives of antinociceptive activity, as well as drugs modulating blood coagulation processes.

## Figures and Tables

**Table 1:** ^1^H and ^13^C NMR spectroscopic data for compound **1**.

Position	δH, (J in Hz)	δC
1	6.02 (1H, d, J = 10.1)	92.7
3	6.91 (1H, s)	148.4
4	-	109.1
5	-	139.6
6	5.65 (1H, dd, J = 3.3, 2.6)	117.6
7	5.41 (1H, d, J = 2.6)	82.8
8	-	80.2
9	2.93 (1H, dd, J = 10.0, 2.6)	49.4
10a	3.71 (1H, d, J = 11.1)	48.9
10b	3.97 (1H, d, J = 11.1)	
11a	4.61 (1H, d, J = 12.3)	60.5
11b	4.78 (1H, d, J = 12.3)	
1′	-	170.7
2′	2.34 (2H, dd, J = 9.2, 7.4)	43.0
3′	2.04 (1H, m)	25.5
4′	0.95 (3H, d, J = 6.7)	22.48
5′	0.95 (3H, d, J = 6.7)	22.50
1″	-	172.6
2″	2.17 (2H, dd, J = 7.4, 1.2)	42.7
3″	1.94 (1H, m)	25.6
4″, 5″	1.90 (6H, d, J = 6.5)	22.6
1‴	-	169.8
2‴	2.00 (3H, s)	21.2
1⁗	-	174.2
2⁗	2.08 (1H, d, J = 6.9)	43.3
3⁗	1.93 (1H, m)	25.7
4⁗	0.88 (3H, d, J = 6.9)	22.45
5⁗	0.87 (3H, d, J = 6.9)	22.37

**Table 2:** ^1^H and ^13^C NMR spectroscopic data for compound **2**.

Position	δH, (J in Hz)	δC
1	6.00 (1H, d, J = 10.1)	92.8
3	6.92 (1H, s)	148.5
4	-	109.1
5	-	139.1
6	5.65 (1H, dd, J = 3.4, 2.5)	117.7
7	5.46 (1H, d, J = 2.6)	83.2
8	-	79.1
9	2.96 (1H, dd, J = 10.2, 2.3)	48.2
10a	4.32 (1H, d, J = 11.3)	66.6
10b	4.15 (1H, d, J = 11.3)	
11a	4.62 (1H, d, J = 12.3)	60.6
11b	4.77 (1H, d, J = 12.3)	
1′	-	170.8
2′	2.30 (2H, dd, J = 7.3, 3.8)	42.7
3′	2.05 (1H, m)	25.6
4′, 5′	0.90 (6H, m)	22.5
1″	-	172.6
2″	2.17 (1H, d, J = 6.8)	43.0
3″	1.95 (1H, m)	25.7
4″, 5″	0.90 (6H, m)	22.5
1‴	-	169.9
2‴	1.95 (3H, s)	21.0
1⁗	-	169.3
2⁗	4.73 (1H, d, J = 4.7)	73.2
3⁗	2.20	29.7
4⁗	0.90 (3H, m)	17.4
5⁗	0.94 (3H, m)	19.0
6⁗	-	172.3
7⁗	2.17 (1H, d, J = 6.8)	42.9
8⁗	1.95 (1H, m)	25.8
9⁗	0.94 (3H, m)	22.5
10⁗	0.92	22.5

**Table 3:** ^1^H and ^13^C NMR spectroscopic data for compound **3**.

Position	δH, (J in Hz)	δC
1a	2.30 (1H, dd, J = 18.7, 6.8)	37.7
1b	2.04 (1H, dd, J = 18.7, 1.2)	
2	-	208.2
3	-	134.6
4	-	172.1
5	3.90 (1H, dd, J = 9.7, 4.1)	36.0
6a	1.90 (1H, m)	25.6
6b	1.57 (1H, m)	
7a	1.49 (1H, m)	28.0
7b	1.73 (1H, m)	
8	2.23 (1H, m)	32.5
9	3.07 (1H, brs)	40.4
10	1.63 (3H, d, J = 1.8)	8.1
11	6.94 (1H, dd, J = 9.7, 1.5)	139.8
12	-	129.2
13	1.83 (3H, d, J = 1.4)	13.0
14	-	169.2
15	0.66 (3H, d, J = 7.0)	11.5

## Data Availability

The data presented in this study are available on request from the corresponding authors.

## Supplementary Materials

The following supporting information can be downloaded at: https://www.mdpi.com/article/10.3390/ijms27198756/s1.

## Abbreviations

2APB	2-aminoethoxydiphenyl borate
Aβ	Amyloid beta
AU	Arbitrary units
BDNF	Brain-derived neurotrophic factor
CHO	Chinese hamster ovary
COSY	Correlation spectroscopy
CE	Collision energy
DEPT	Distortionless enhancement by polarization transfer
DMSO	Dimethyl sulfoxide
DP	Declustering potential
DPPH	2-diphenyl-1-picrylhydrazyl
EDTA	Ethylenediaminetetraacetic acid
EGTA	Ethylene glycol-bis(β-aminoethyl ether)-N,N,N′,N′-tetraacetic acid
FBS	Fetal bovine serum
HMBC	Heteronuclear multiple bond correlation
HPLC	High-performance liquid chromatography
HR-ESI-MS	High resolution mass spectrometry
GABA	γ-aminobutyric acid
5-HT5A	5-hydroxytryptamine (serotonin) receptor 5A
CGS buffer	A sterilized solution optimized for platelet washing
LC-MS	Liquid chromatography-mass spectrometry
MS/MS	Tandem mass spectrometry
MSn	Multistaged tandem mass spectrometry
MTT	3-(4,5-dimethylthiazol-2-yl)-2,5-diphenyltetrazolium bromide
NF-kB	Nuclear factor ‘kappa-light-chain-enhancer’ of activated B-cells
NMR	Nuclear magnetic resonance
PBS	Phosphate buffered saline
PDA	Photodiode array
PKC	Protein kinase C
PLC	Phospholipase C
PRP	Platelet-rich plasma
QqTOF-MS	Quadrupole-time of flight mass spectrometry
RP-HPLC-UV	Reversed-phase high-performance liquid chromatography with ultraviolet detection
RP-UHPLC-HR-MS	Reversed-phase ultrahigh-performance liquid chromatography—high resolution mass spectrometry
RT	Room temperature
SDS	Sodium dodecyl sulfate
SH-SY5Y	A cloned subline of a neuroblastoma cell line SK-N-SH from a metastatic bone tumor
TFA	Trifluoroacetic acid
TLC	Thin layer chromatography
TRPV1 channels	The transient receptor potential cation channel subfamily V member 1 (TRPV1)
Type A (GABA_A_) receptors	Gamma-aminobutyric acid type A receptors
UV	Ultraviolet
WP	Washed platelets
