# Supplementary material for: A First Insight into the Biological Activity Profile of Individual Compounds of Valeriana officinalis L
Source: Int J Mol Sci. 2026 Sep 30;27(19):8756. doi: 10.3390/ijms27198756 (PMC13664934; doi:10.3390/ijms27198756)
Supplement: Supplementary file 1 [file ijms-27-08756-s001.zip › ijms-4503414-supplementary.pdf]

A first insight at the mechanism of neuroprotective activity and side effects of individual compounds of Valeriana officinalis L.

Supplementary information

Directory

Table S1 Purity and sufficient yields of compounds isolated from the dichloromethane fraction of the total extract from the roots of *Valeriana officinalis* L. 6

Table S2 ^{1}H and ^{13}C NMR spectroscopic data for iridoidvol A (4) 7

The spectra were acquired at 400 and 100 MHz respectively, DMSO-*d6*, temperature 25°C........7

Table S3 ^{1}H and ^{13}C NMR spectroscopic data for isovaltrate isovaleroyloxyhydrin (5)..............8

The spectra were acquired at 400 and 100 MHz respectively, DMSO-*d6*, temperature 25°C........8

Table S4 ^{1}H and ^{13}C NMR spectroscopic data for valtrate hydrine B_1 (6) (400 and 100 MHz respectively, DMSO-*d6*, temperature 25°C) 9

The spectra were acquired at 400 and 100 MHz respectively, DMSO-*d6*, temperature 25°C........9

Table S5 ^{1}H and ^{13}C NMR spectroscopic data for valerenic acid (7) 10

The spectra were acquired at 400 and 100 MHz respectively, DMSO-*d6*, temperature 25°C......10

Table S6 ^{1}H and ^{13}C NMR spectroscopic data for hydroxyvalerenic acid (8) 11

The spectra were acquired at 400 and 100 MHz respectively, DMSO-*d6*, temperature 25°C......11

Table S7 ^{1}H and ^{13}C NMR spectroscopic data for acetoxyvalerenic acid (9)..............................12

The spectra were acquired at 400 and 100 MHz respectively, DMSO-*d6*, temperature 25°C......12

Figures 13

Figure S1 RP-HPLC-UV chromatogram of the total ethanolic extract of *Valeriana officinalis* roots. The chromatogram was obtained using a Shimadzu Prominence LC20 high-performance liquid chromatograph with diode array detector. The figure shows the chromatogram at a wavelength of 254 nm. 13

....14

Figure S2 Preparative HPLC-UV chromatograms of the dichloromethane fractions of *Valeriana officinalis* roots.

Figure S3. ^{1}H NMR spectrum of compound 1. 15

Figure S4. COSY spectrum of compound 1. 16

Figure S5. ^{13}C NMR spectrum of compound 1. 17

Figure S6. DEPT spectrum of compound 1...18

Figure S7. HSQC spectrum of compound 1. 19

Figure S8. HBMC spectrum of compound 1. 20

Figure S9. ^{1}H NMR spectrum of compound 2. 21

Figure S10. COSY spectrum of compound 2. 22

Figure S11. ^{13}C spectrum of compound 2. 23

Figure S12. DEPT spectrum of compound 2...24

Figure S13. HSQC spectrum of compound 2. 25

Figure S14. HMBC spectrum of compound 2. 26

Figure S15. ^{1}H NMR spectrum of compound 3. 27

Figure S16. COSY spectrum of compound 3. 28

Figure S17. ^{13}C NMR spectrum of compound 3. 29

Figure S18. DEPT spectrum of compound 3...30

Figure S19. HSQC spectrum of compound 3. 31

Figure S20. HMBC spectrum of compound 3. 32

Figure S21. Tandem mass spectrum of *m/z* 625.3 at t_R 7.4 min corresponding to isovaltrate α-(isovaleroxy)isovaleroxyhydrin (2) and proposed fragmentation patterns. The spectrum was

acquired with a hybrid QqTOF mass spectrometer operated in the positive product ion mode with unit Q1 resolution (collision energy 30 eV). 33

Figure S22. Tandem mass spectrum of *m/z* 247.1 at t_R 4.9 min corresponding to 2-oxovalerenic acid (3) and proposed fragmentation patterns. The spectrum was acquired with a hybrid QqTOF mass spectrometer operated in the negative product ion mode with unit Q1 resolution (collision energy 20 eV). 34

Figure S23. Tandem mass spectrum of *m/z* 715.5 at t_R 9.9 min corresponding to iridoidvol A (4) and proposed fragmentation patterns. The spectrum was acquired with a hybrid QqTOF mass spectrometer operated in the positive product ion mode with unit Q1 resolution (collision energy 30 eV). 35

Figure S24. Tandem mass spectrum of *m/z* 525.3 at t_R 8.7 min corresponding to isovaltrate isovaleroyloxyhydrin (5) and proposed fragmentation patterns. The spectrum was acquired with a hybrid QqTOF mass spectrometer operated in the positive product ion mode with unit Q1 resolution (collision energy 20 eV). 36

Figure S25. Tandem mass spectrum of *m/z* 525.3 at t_R 8.6 min corresponding to valtrate hydrine B1 (6) and proposed fragmentation patterns. The spectrum was acquired with a hybrid QqTOF mass spectrometer operated in the positive product ion mode with unit Q1 resolution (collision energy 20 eV). 37

Figure S26. Tandem mass spectrum of *m/z* 233.2 at t_R 7.8 min corresponding to valerenic acid (7) and proposed fragmentation patterns. The spectrum was acquired with a hybrid QqTOF mass spectrometer operated in the negative product ion mode with unit Q1 resolution (collision energy 30 eV). 38

Figure S27. Tandem mass spectrum of *m/z* 249.2 at t_R 5.6 min corresponding to hydroxyvalerenic acid (8) and proposed fragmentation patterns. The spectrum was acquired with a hybrid QqTOF

mass spectrometer operated in the negative product ion mode with unit Q1 resolution (collision energy 30 eV). 39

Figure S28. Tandem mass spectrum of *m/z* 291.2 at t_R 6.6 min corresponding to acetoxyvalerenic acid (9) and proposed fragmentation patterns. The spectrum was acquired with a hybrid QqTOF mass spectrometer operated in the negative product ion mode with unit Q1 resolution (collision energy 20 eV). 40

Figure S29. Neuroprotective effects of individual compounds isolated from the roots of Valeriana officinalis L. in the model of Aβ25-35 induced cytotoxicity 41

Synthesis Aβ25-35..42

Table S1 Purity and sufficient yields of compounds isolated from the dichloromethane fraction of the total extract from the roots of *Valeriana officinalis* L**.**

**Compound**	**Purity, %**	**Sufficient yields, %**
Isovaltrate isovaleroyloxyisohydrin (**1**)	96.54	0.082
Isovaltrate α-(isovaleroxy)isovaleroxyhydrin (**2**)	91.23	0.036
2-Oxovalerenic acid (**3**)	93.14	0.060
Iridoidvol A (**4**)	92.76	0.158
Isovaltrate isovaleroyloxyhydrin (**5**)	92.35	0.123
Valtrate hydrine B_1 (**6**)	90.44	0.011
Valerenic acid (**7**)	95.29	0.069
Hydroxyvalerenic acid (**8**)	94.89	0.254
Acetoxyvalerenic acid (**9**)	96.74	0.097

Table S2 ^{1}H and ^{13}C NMR spectroscopic data for iridoidvol A (**4**)

**Position**	***δ*H, (J in Hz)**	***δ*C**
1	6.03 (1H, d, *J* = 10.1)	92.9
3	6.92 (1H, s)	148.5
4	-	109.1
5	-	139.4
6	5.65 (1H, dd, *J* = 3.5, 2.5)	117.6
7	5.56 (1H, d, *J* = 2.8)	83.3
8	-	79.4
9	2.95 (1H, dd, *J* = 10.2, 2.0 Hz)	48.4
10a	4.38 (1H, d, *J* = 11.4)	66.4
10b	4.17 (1H, d, *J* = 11.4)	
11a	4.63 (1H, d, *J* = 12.2)	60.6
11b	4.77 (1H, d, *J* = 12.2)	
1'	-	170.8
2'	2.32 (2H, d, *J* = 6.9)	42.7
3'	2.05 (1H, m)	25.5
4', 5'	0.94 (6H, d, *J* = 6.5)	22.5
1''	-	172.6
2''	2.17 (1H, d, *J* = 6.5)	43.0
3''	1.95 (1H, m)	25.5
4'', 5''	0.89 (6H, m)	22.5
1'''	-	169.7
2'''	1.88 (3H, s)	21.0
1''''	-	132.0
2''''	3.59 (1H, dd, *J* = 10.3, 4.7)	34.0
3a''''	1.72 (1H, m)	25.5
3b''''	1.34 (1H, m)	
4a''''	1.37 (1H, m)	28.1
4b''''	1.88 (1H, m)	
5''''	2.20 (1H, m)	31.3
6''''	2.83 (1H, brs)	54.4
7''''	4.95 (1H, m)	75.5
8a''''	2.59 (1H, dd, *J* = 17.8, 7.1)	44.6
8b''''	2.17 (1H, m)	
9''''	-	128.4
10''''	6.91 (1H, m)	142.8
11''''	-	126.3
12''''	-	167.6
13''''	1.82 (3H, brs)	12.7
14''''	0.76 (3H, d, *J* = 6.7)	12.9
15''''	1.65 (3H, brs)	13.6
16''''	-	170.8
17''''	1.96 (3H, s)	21.4

The spectra were acquired at 400 and 100 MHz respectively, DMSO-*d6*, temperature 25°C

Table S3 ^{1}H and ^{13}C NMR spectroscopic data for isovaltrate isovaleroyloxyhydrin (**5**)

Position	δH, (J in Hz)	δC
1	6.00 (1H, d, *J* = 10.1)	92.7
3	6.90 (1H, s)	148.4
4	-	109.2
5	-	139.3
6	5.65 (1H, dd, *J* = 3.3, 2.6)	117.6
7	5.46 (1H, d, *J* = 2.6)	83.2
8	-	79.1
9	2.91 (1H, dd, *J* = 10.0, 2.6)	48.1
10a	4.21 (1H, d, *J* = 11.4)	65.5
10b	4.17 (1H, d, *J* = 11.4)	
11a	4.61 (1H, d, *J* = 12.3)	60.5
11b	4.78 (1H, d, *J* = 12.3)	
1'	-	170.7
2'	2.31 (2H, dd, *J* = 7.1, 2.4)	42.8
3'	2.05 (1H, m)	25.5
4'	0.94 (3H, d, *J* = 6.8)	22.47
5'	0.94 (3H, d, *J* = 6.8)	22.5
1"	-	172.6
2"	2.17 (1H, d, *J* = 6.8)	43.0
3"	1.95 (1H, m)	25.5
4", 5"	0.90 (6H, d, *J* = 6.8)	22.5
1"'	-	169.8
2"'	1.92 (3H, s)	21.1
1"'	-	172.3
2"'	2.17 (1H, d, *J* = 6.8)	43.0
3"'	1.95 (1H, m)	25.8
4"'	0.88 (3H, d, *J* = 6.8)	2.36
5"'	0.88 (3H, d, *J* = 6.8)	2.44

The spectra were acquired at 400 and 100 MHz respectively, DMSO-*d6*, temperature 25°C

Table S4 ^{1}H and ^{13}C NMR spectroscopic data for valtrate hydrine B_1 (**6**) (400 and 100 MHz respectively, DMSO-*d6*, temperature 25°C)

Position	δH, (J in Hz)	δC
1	6.01 (1H, d, *J* = 10.1)	92.8
3	6.91 (1H, s)	148.5
4	-	109.1
5	-	139.2
6	5.70 (1H, dd, *J* = 3.3, 2.5)	117.9
7	5.44 (1H, d, *J* = 2.8)	83.1
8	-	79.2
9	2.93 (1H, dd, *J* = 10.0, 2.3)	48.1
10a	4.16 (1H, d, *J* = 11.2)	65.6
10b	4.21 (1H, d, *J* = 11.2)	
11a	4.61 (1H, d, *J* = 12.1)	60.5
11b	4.71 (1H, d, *J* = 12.1)	
1'	-	170.7
2'	2.31 (2H, d, *J* = 7.2, 1.7)	42.8
3'	2.05 (1H, m)	25.5
4'	0.94 (3H, d, *J* = 6.4)	22.4
5'	0.94 (3H, d, *J* = 6.4)	22.5
1"	-	170.7
2"	1.99 (3H, s)	21.1
1'''	-	171.7
2'''	2.16 (2H, d, *J* = 7.5, 4.5)	43.0
3'''	1.95 (1H, m)	25.5
4''', 5'''	0.89 (6H, d, *J* = 6.5)	22.6
1''''	-	172.3
2''''	2.10 (2H, d, *J* = 7.5, 4.5)	43.1
3''''	1.90 (1H, m)	25.5
4'''',5''''	0.87 (6H, d, *J* = 6.7)	22.5

The spectra were acquired at 400 and 100 MHz respectively, DMSO-*d6*, temperature 25°C

Table S5 ^{1}H and ^{13}C NMR spectroscopic data for valerenic acid (**7**)

**Position**	***δ*H, (J in Hz)**	***δ*C**
1	-	133.8
2	3.50 (1H, dd, *J* = 9.9, 3.7)	34.1
3a	1.72 (1H, m)	25.5
3b	1.34 (1H, m)	
4a	1.38 (1H, m)	28.7
4b	1.75 (1H, m)	
5	1.95 (1H, m)	33.0
6	2.87 (1H, brs)	47.4
7a	1.78 (1H, m)	24.5
7b	1.52 (1H, m)	
8	2.16 (3H, t, *J* = 7.9)	37.4
9	-	130.5
10	6.90 (1H, dd, *J* = 9.8, 1.3)	142.3
11	-	126.6
12	-	169.6
13	1.79 (3H, d, *J* = 1.3)	12.6
14	0.75 (3H, d, *J* = 7.0)	12.2
15	1.61 (3H, brs)	13.6

The spectra were acquired at 400 and 100 MHz respectively, DMSO-*d6*, temperature 25°C

Table S6 ^{1}H and ^{13}C NMR spectroscopic data for hydroxyvalerenic acid (**8**)

Position	*δ*H, (J in Hz)	*δ*C
1	-	132.1
2	3.50 (1H, dd, *J* = 10.0, 3.7)	33.8
3a	1.70 (1H, m)	25.6
3b	1.32 (1H, m)	
4a	1.36 (1H, m)	28.6
4b	1.73 (1H, m)	
5	2.13 (1H, m)	31.1
6	2.59 (1H, brs)	57.2
7	3.99 (1H, dt, *J* = 7.1, 2.8)	71.6
8a	2.42 (1H, dd, *J* = 16.9, 7.1)	47.6
8b	2.03 (1H, brd, *J* = 16.9)	
9	-	128.5
10	6.87 (1H, dd, *J* = 9.8, 1.2)	142.3
11	-	126.6
12	-	169.6
13	1.81 (3H, d, *J* = 1.3)	12.7
14	0.70 (3H, d, *J* = 6.9)	13.2
15	1.60 (3H, brs)	13.7

The spectra were acquired at 400 and 100 MHz respectively, DMSO-*d6*, temperature 25°C

Table S7 ^{1}H and ^{13}C NMR spectroscopic data for acetoxyvalerenic acid (**9**)

Position	δH, (J in Hz)	δC
1	-	132.0
2	3.50 (1H, dd, *J* = 9.9, 4.1)	33.9
3a	1.70 (1H, m)	25.5
3b	1.35 (1H, m)	
4a	1.37 (1H, m)	28.4
4b	1.76 (1H, m)	
5	2.20 (1H, m)	31.2
6	2.77 (1H, brs)	54.8
7	4.95 (1H, dt, *J* = 7.8, 2.1)	75.3
8a	2.59 (1H, dd, *J* = 17.5, 7.8)	44.6
8b	2.17 (1H, brd, *J* = 17.5)	
9	-	128.4
10	6.87 (1H, dd, *J* = 9.8, 1.3)	141.6
11	-	127.1
12	-	169.4
13	1.81 (3H, d, *J* = 1.3)	12.6
14	0.75 (3H, d, *J* = 7.1)	12.8
15	1.64 (3H, brs)	13.4
CO	-	170.8
CH_3	1.97 (3H, s)	21.4

The spectra were acquired at 400 and 100 MHz respectively, DMSO-*d6*, temperature 25°C

Figures

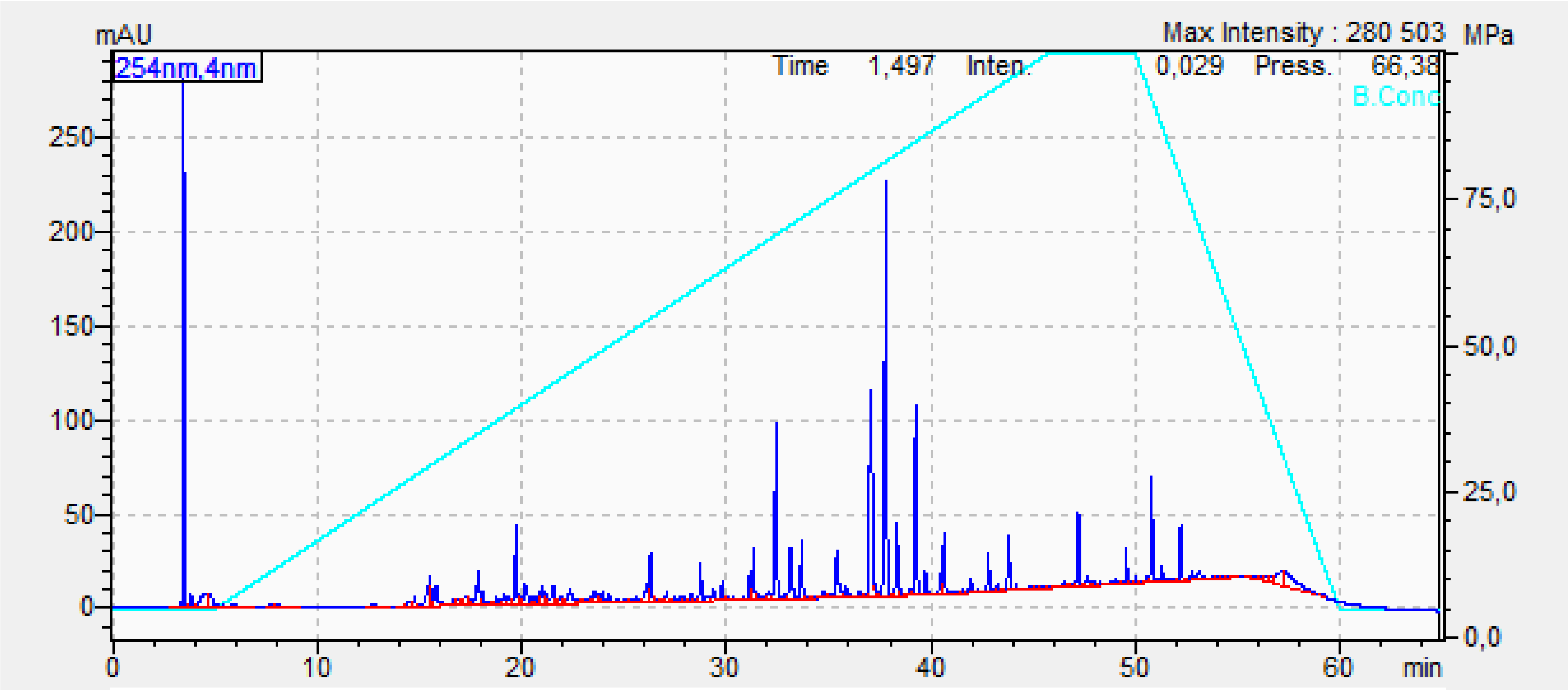

Figure S1 RP-HPLC-UV chromatogram of the total ethanolic extract of *Valeriana officinalis* roots. The chromatogram was obtained using a Shimadzu Prominence LC20 high-performance liquid chromatograph with diode array detector. The figure shows the chromatogram at a wavelength of 254 nm.

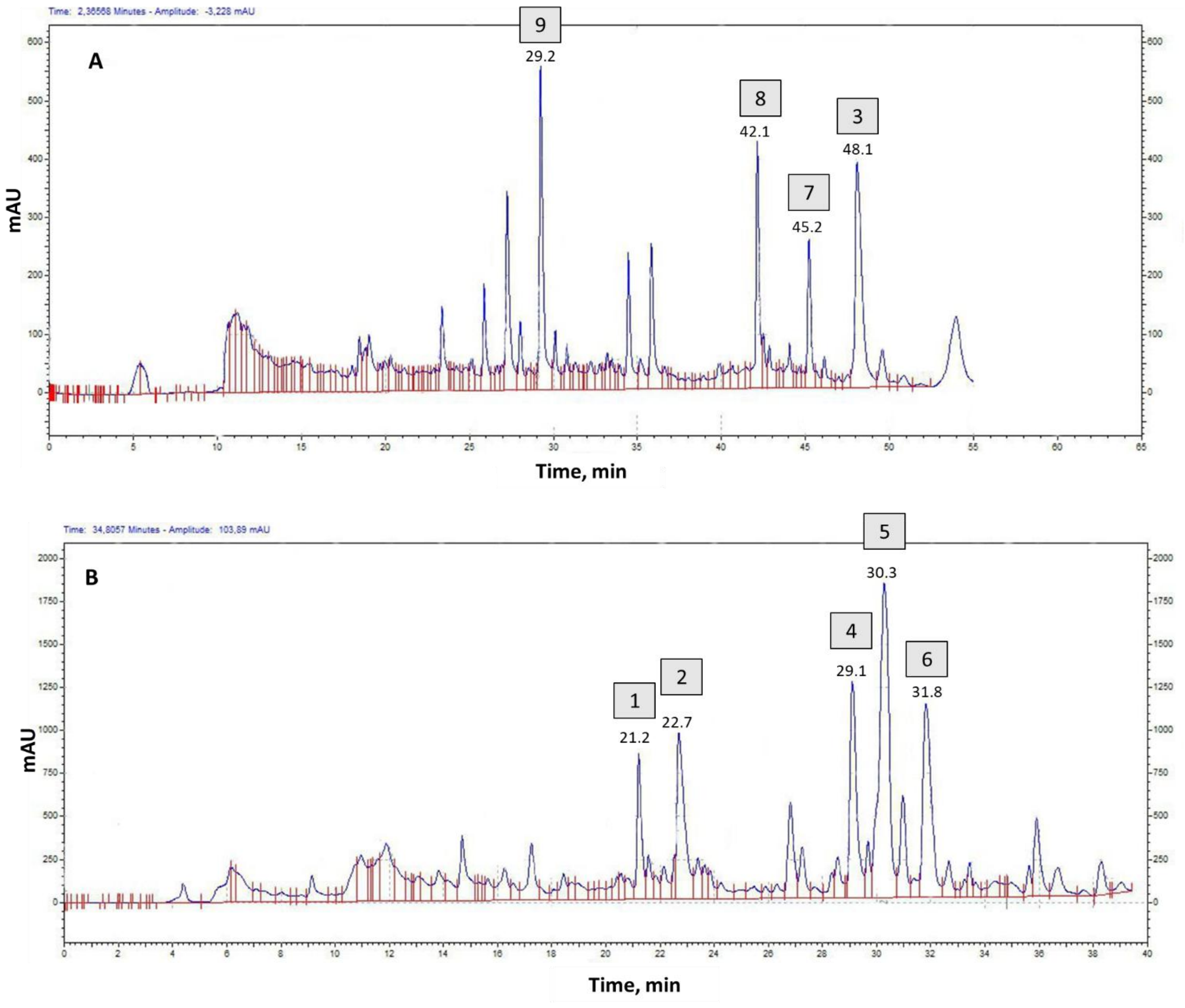

Figure S2 Preparative HPLC-UV chromatograms of the dichloromethane fractions of *Valeriana officinalis* roots. The chromatogram was obtained using a Knauer Smartline system (Knauer Wissenschaftliche Geräte GmbH, Berlin, Germany): A. Preparative HPLC-UV chromatogram of fraction 5, obtained at a wavelength of 254 nm; B. Preparative HPLC-UV chromatogram of fraction 6, obtained at a wavelength of 254 nm. **1-9** – compounds isolated by preparative RP-HPLC-UV at a sufficient quantity and a purity above 90%.

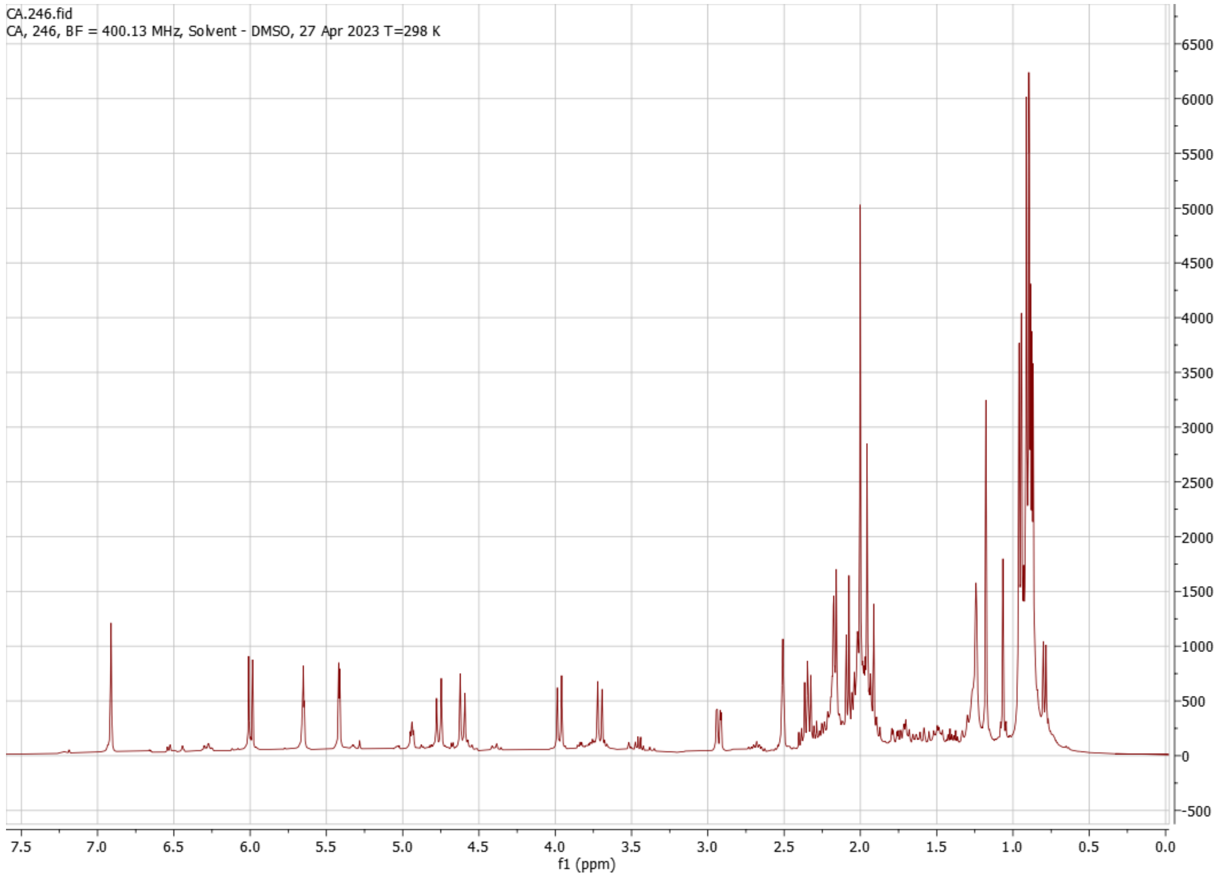

Figure S3. ^{1}H NMR spectrum of compound **1**.

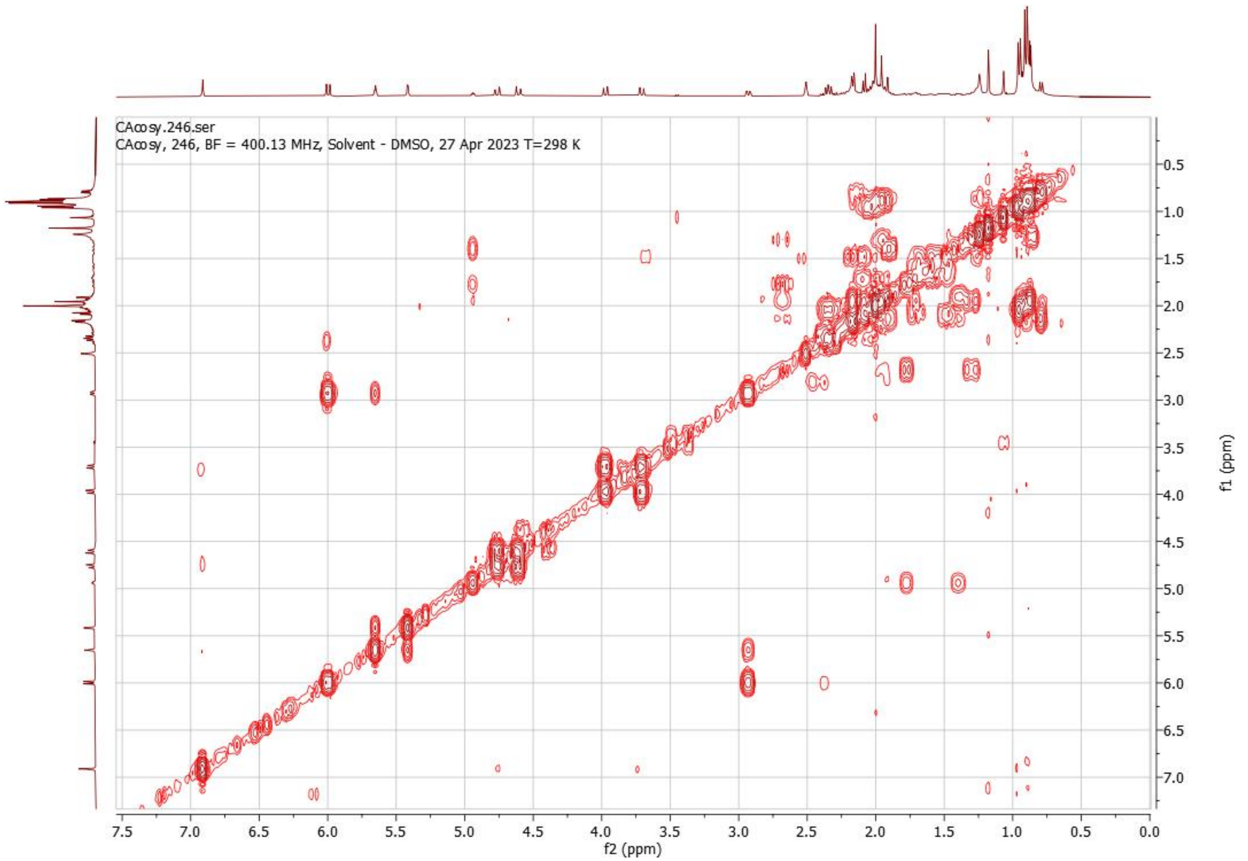

Figure S4. COSY spectrum of compound **1**.

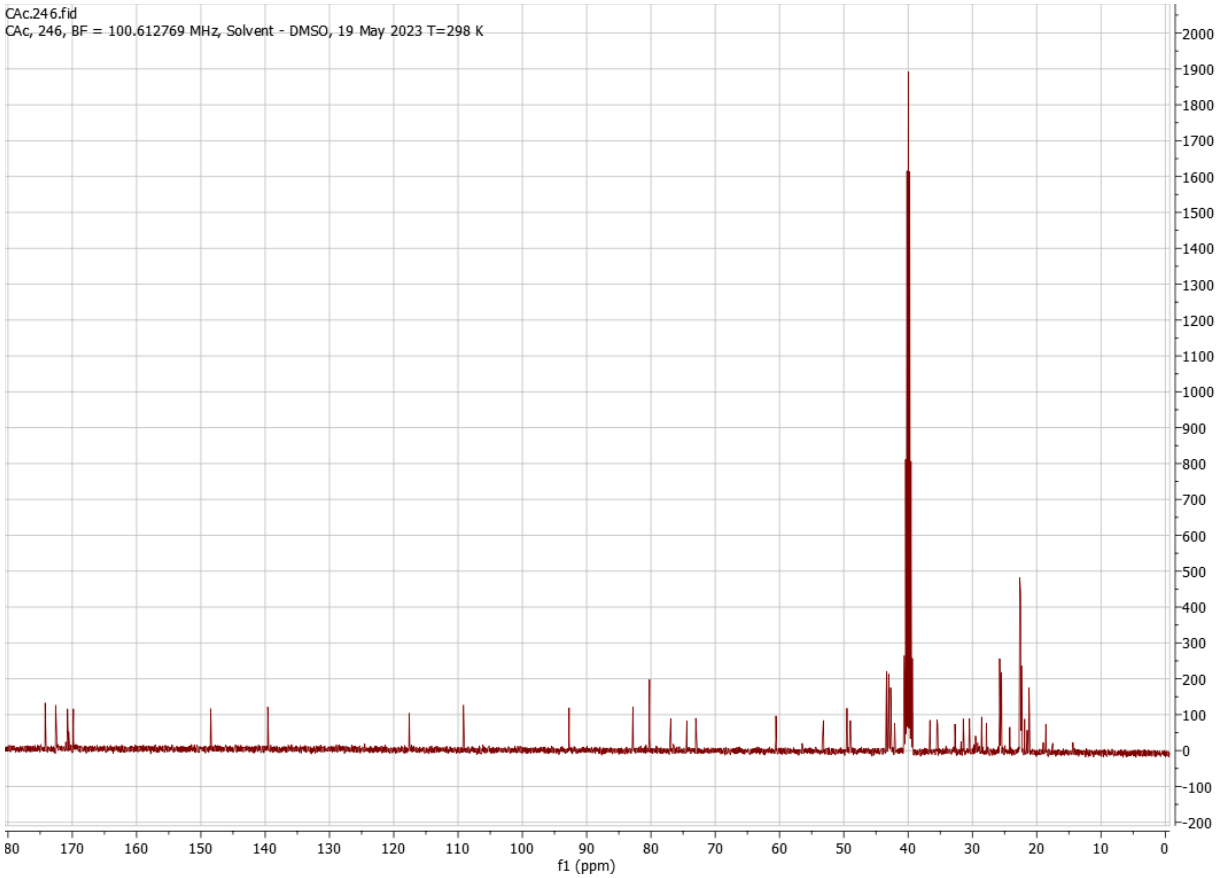

Figure S5. ^{13}C NMR spectrum of compound **1**.

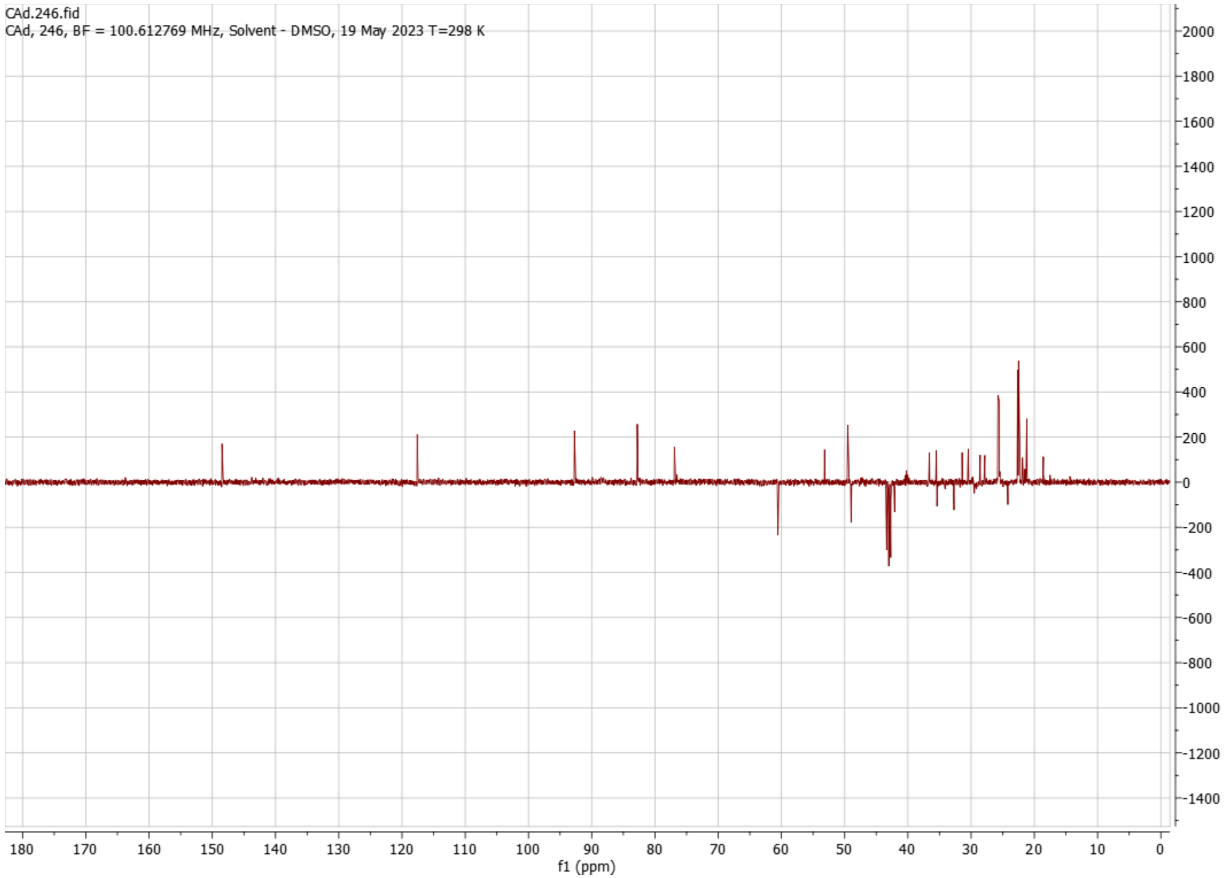

Figure S6. DEPT spectrum of compound **1**.

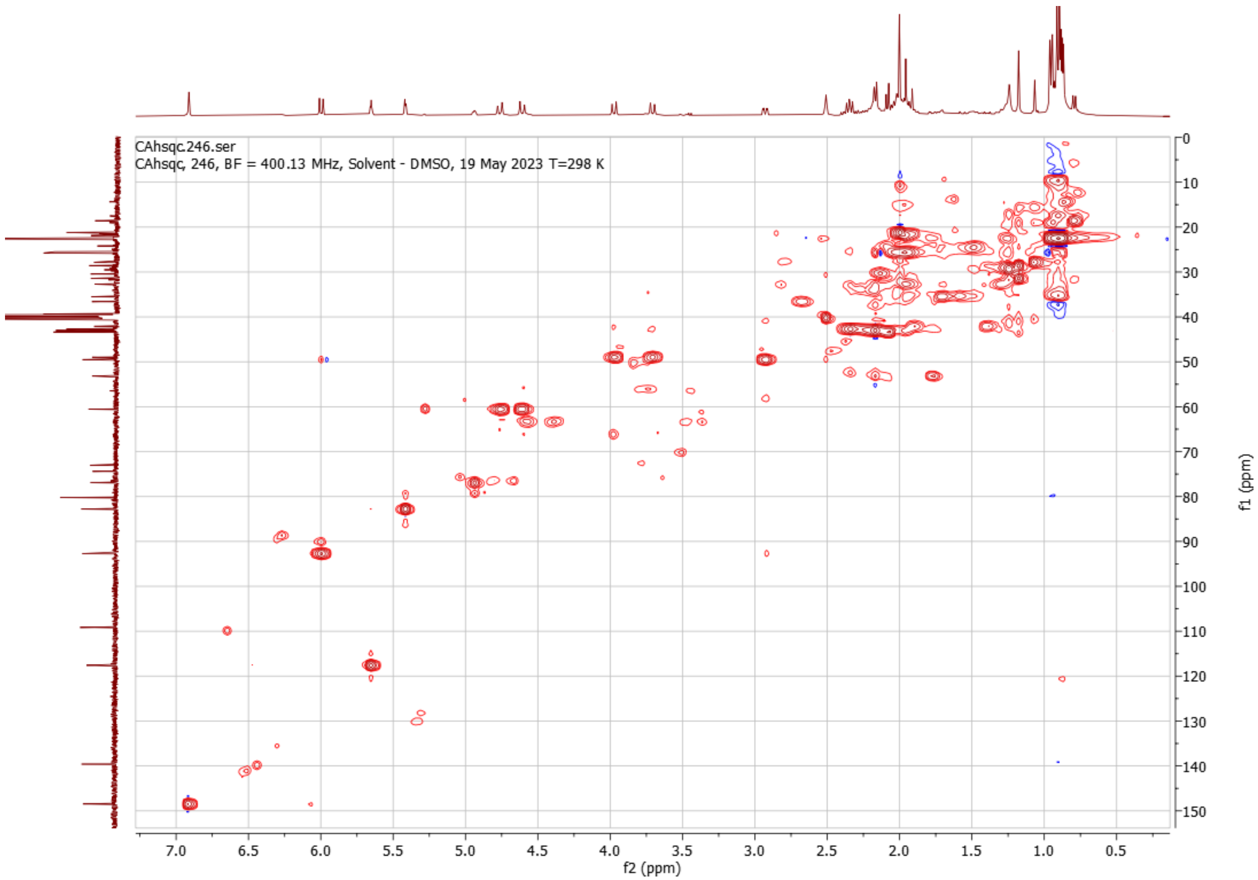

Figure S7. HSQC spectrum of compound **1**.

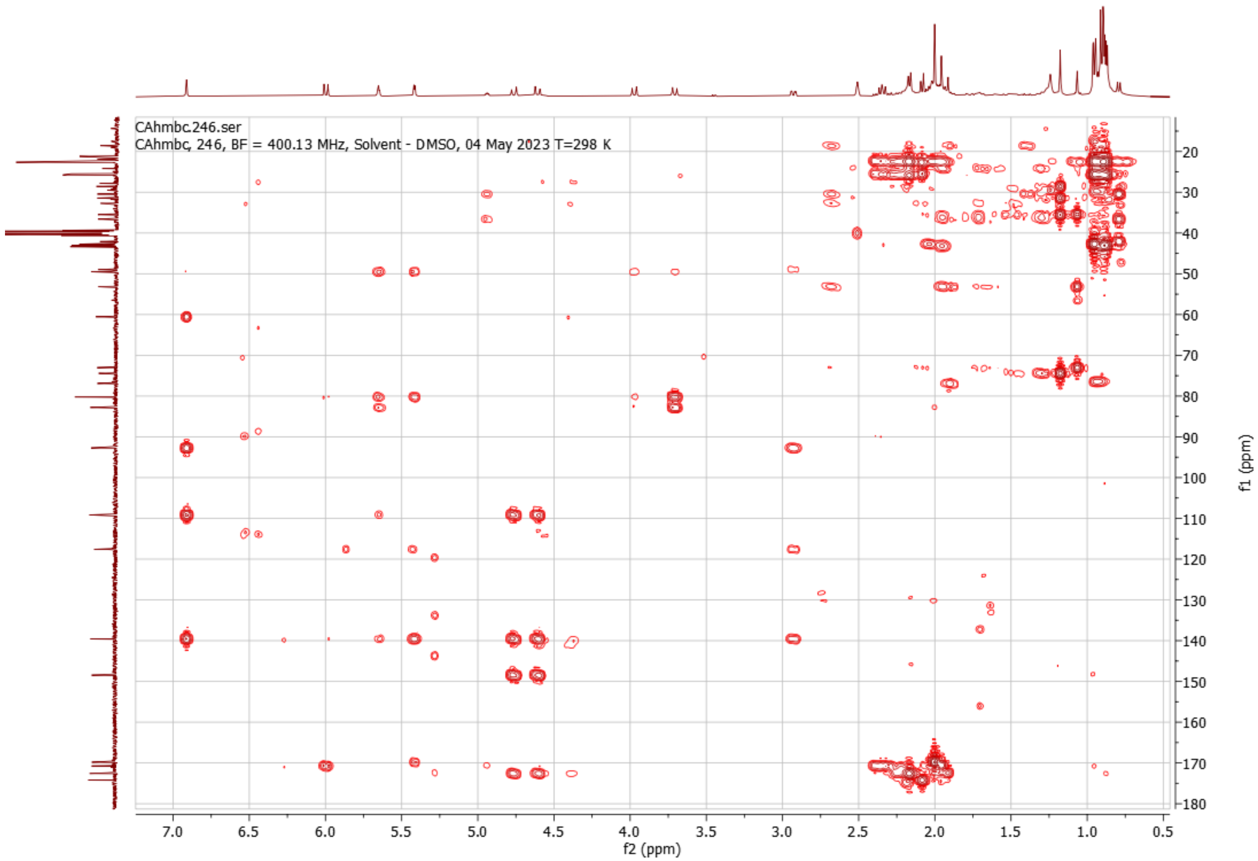

Figure S8. HBMC spectrum of compound **1**.

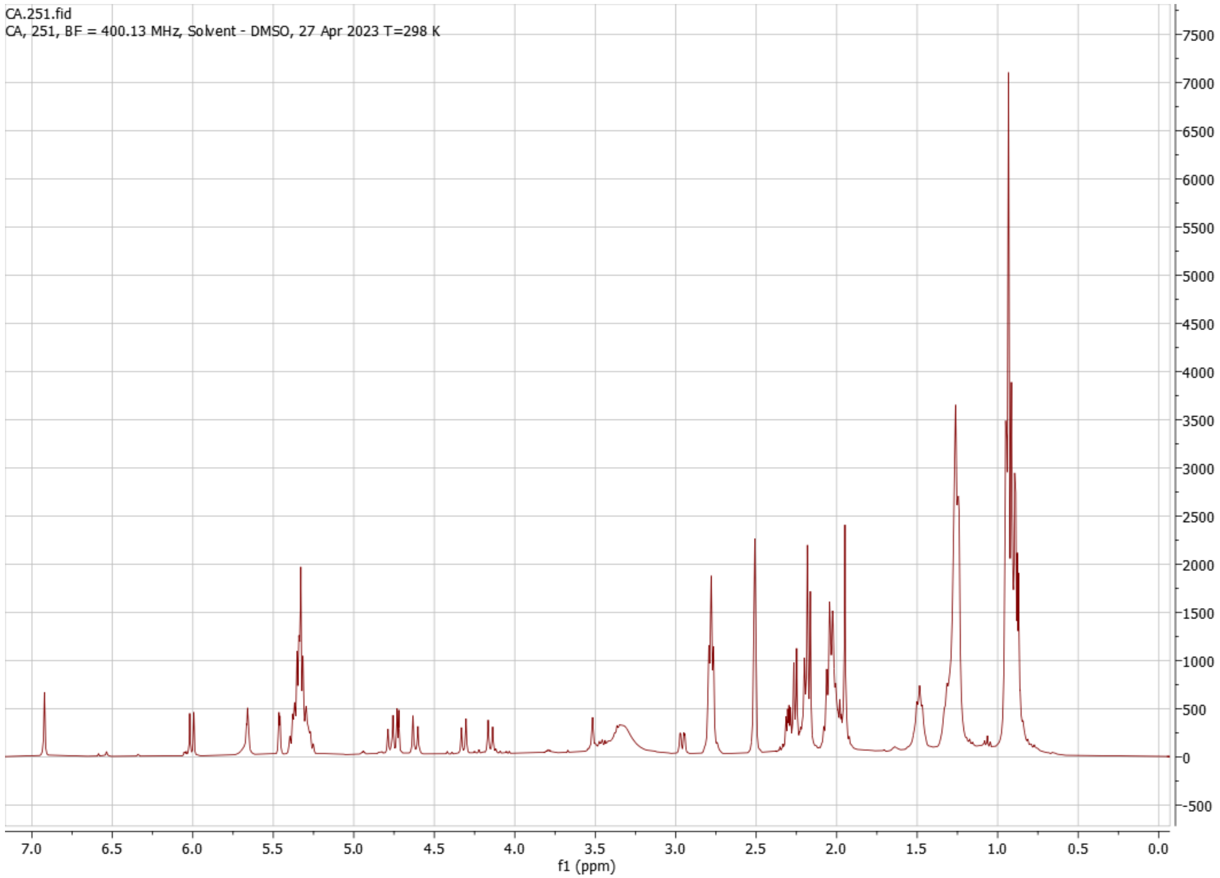

Figure S9. ^{1}H NMR spectrum of compound **2**.

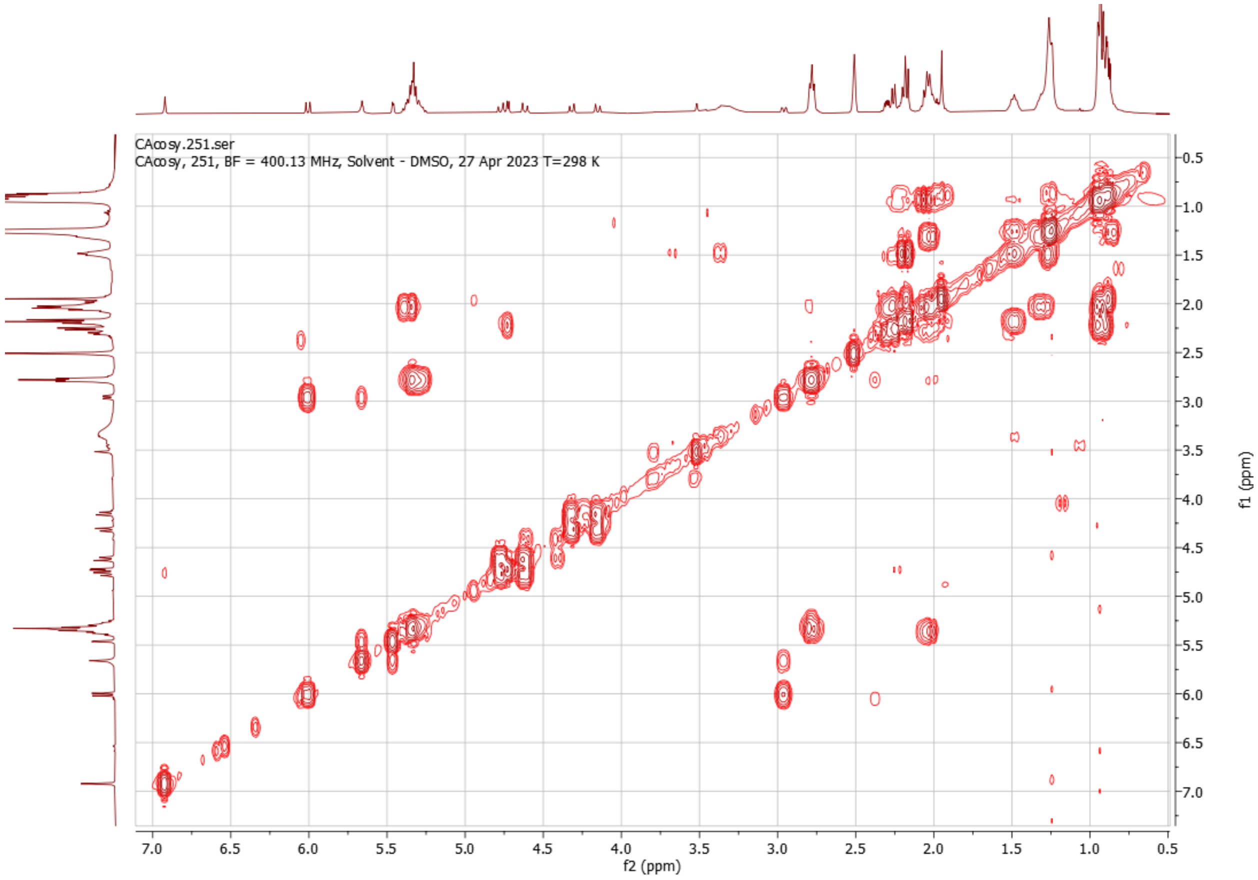

Figure S10. COSY spectrum of compound **2**.

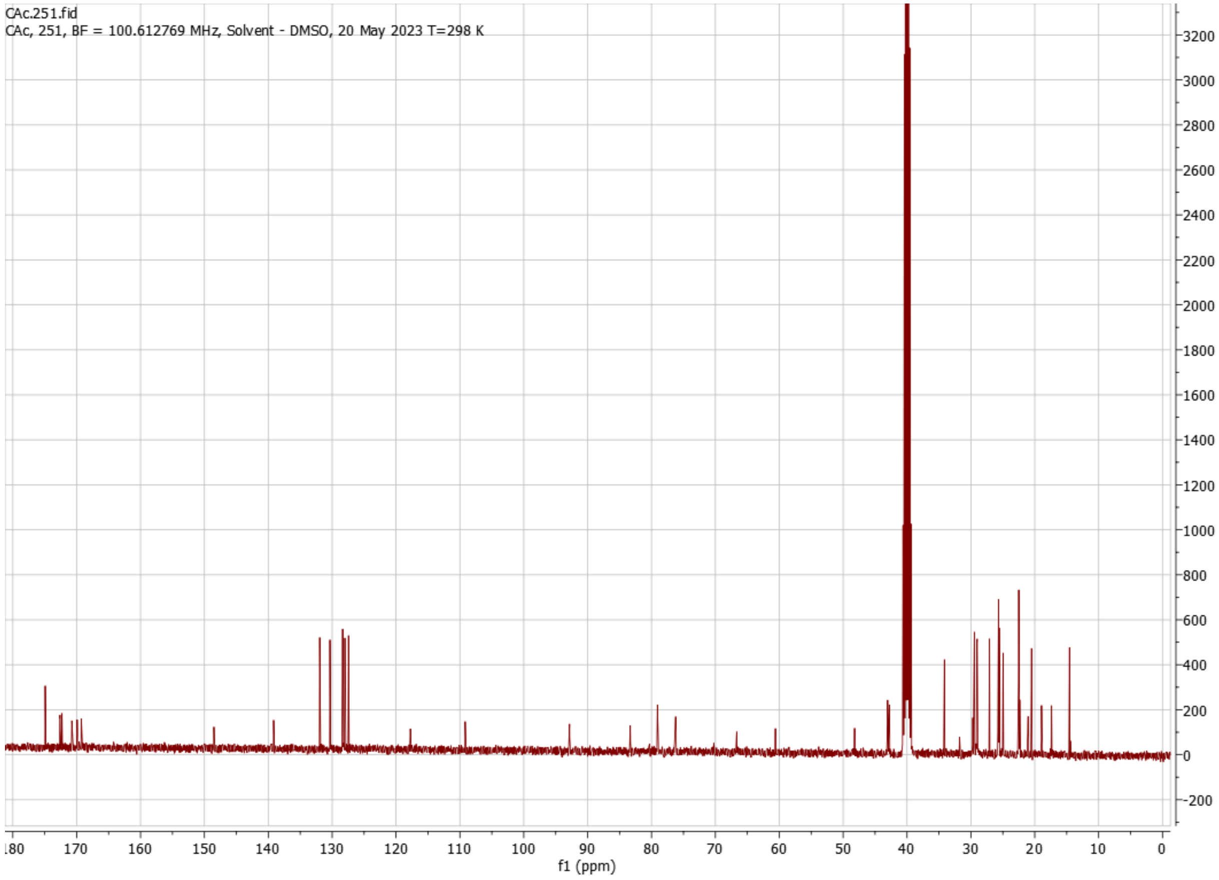

Figure S11. ^{13}C spectrum of compound **2**.

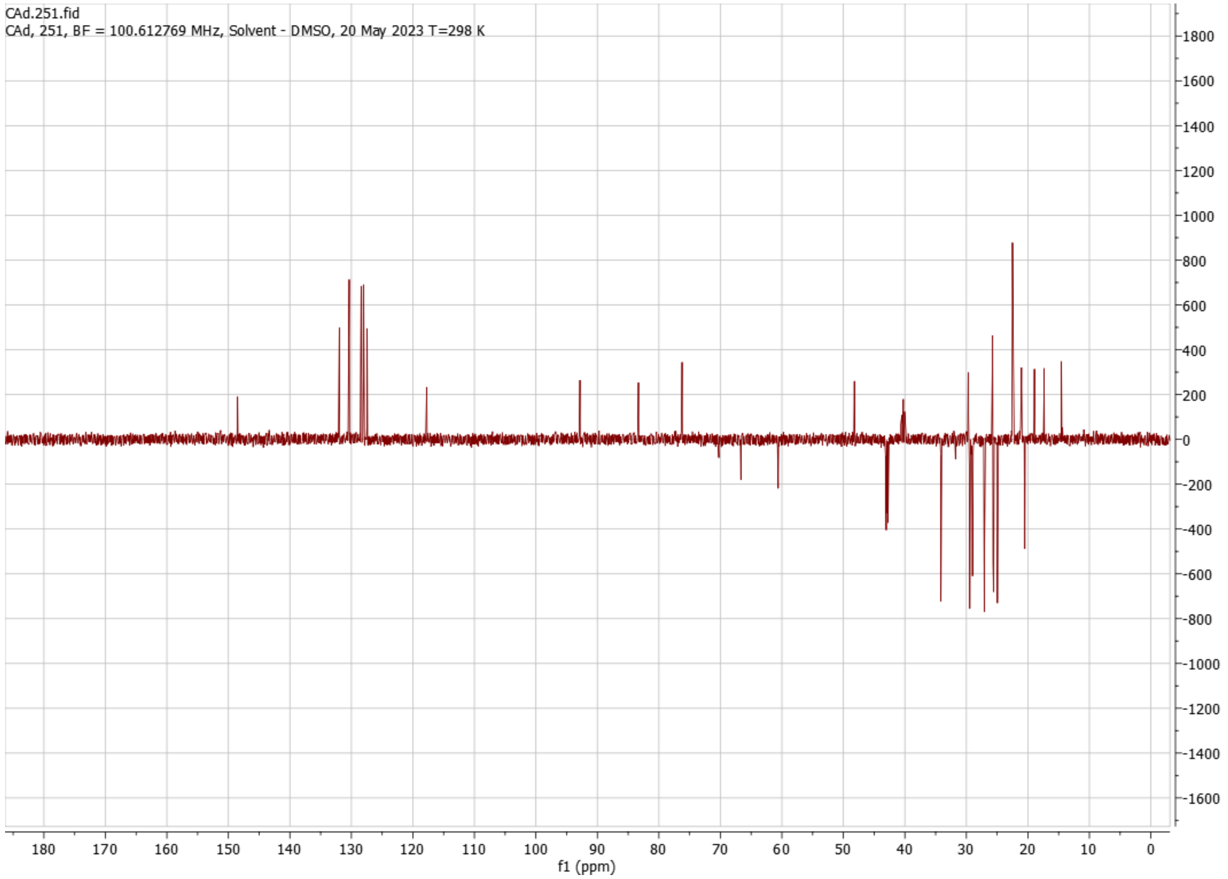

Figure S12. DEPT spectrum of compound **2**.

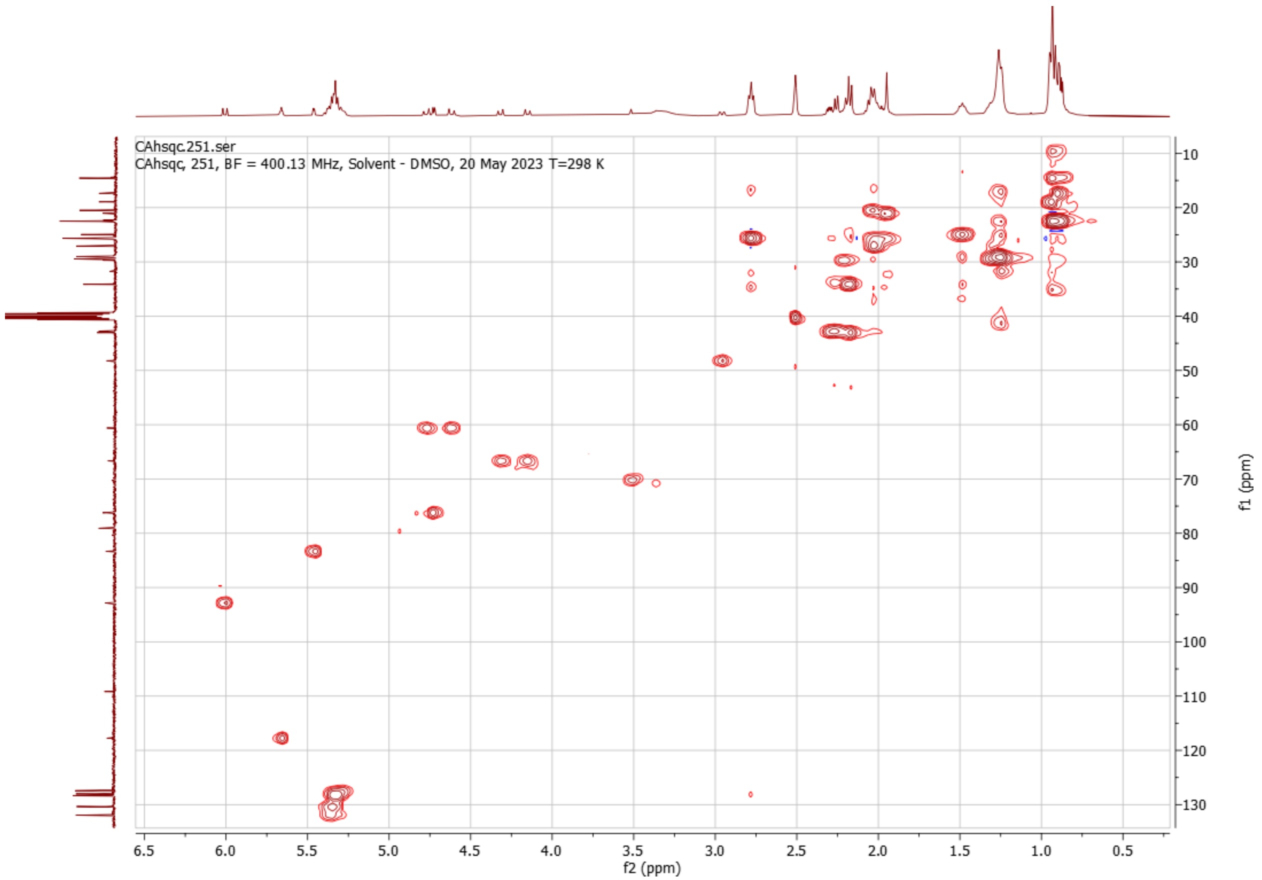

Figure S13. HSQC spectrum of compound **2**.

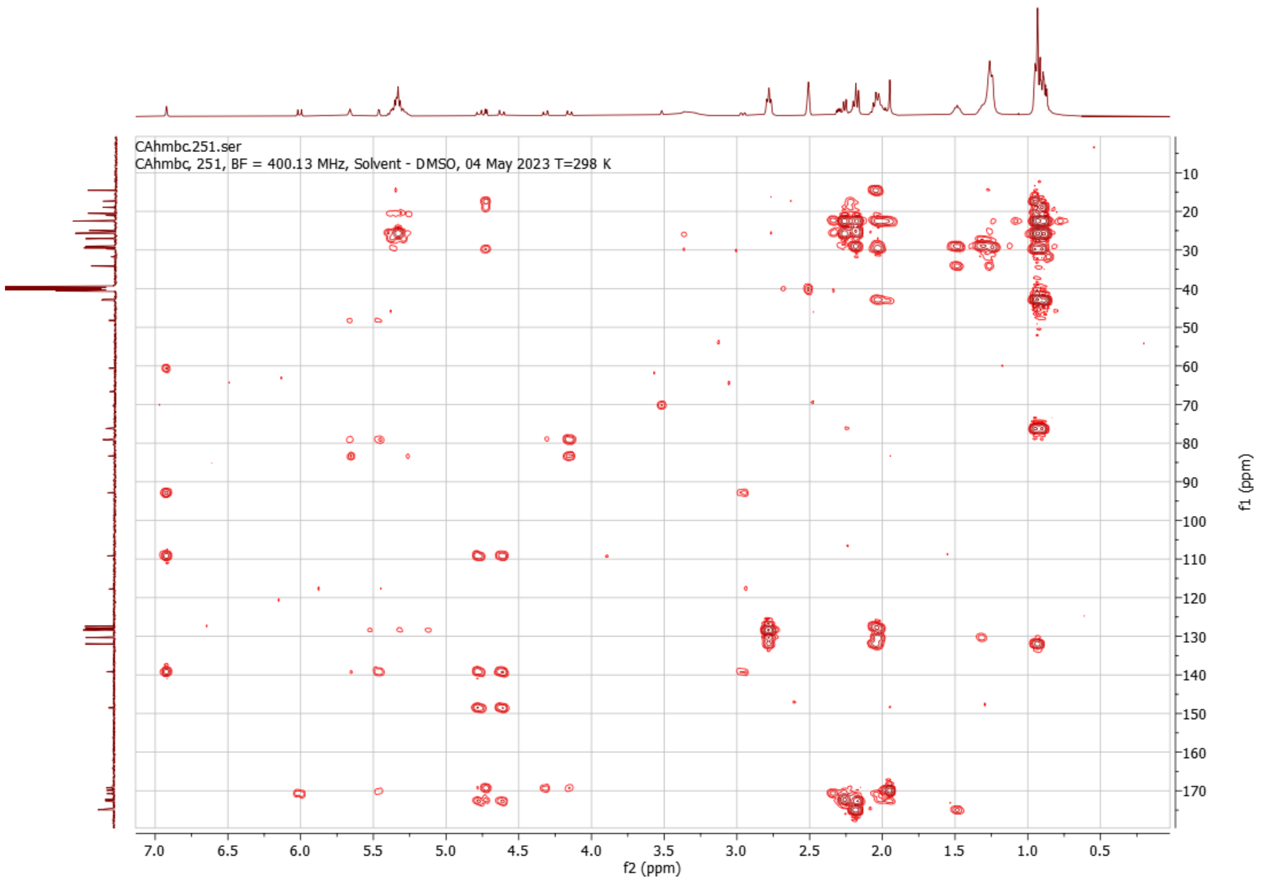

Figure S14. HMBC spectrum of compound **2**.

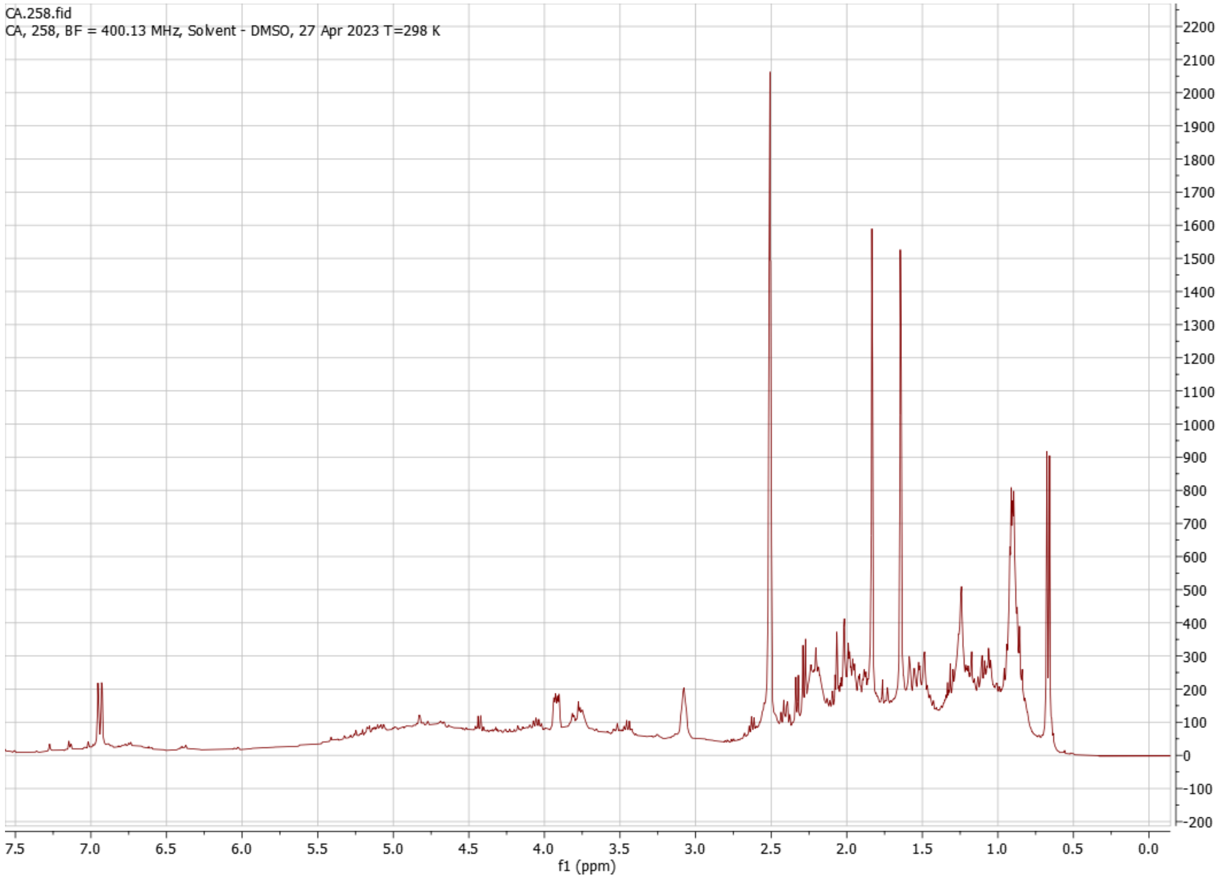

Figure S15. ^{1}H NMR spectrum of compound **3**.

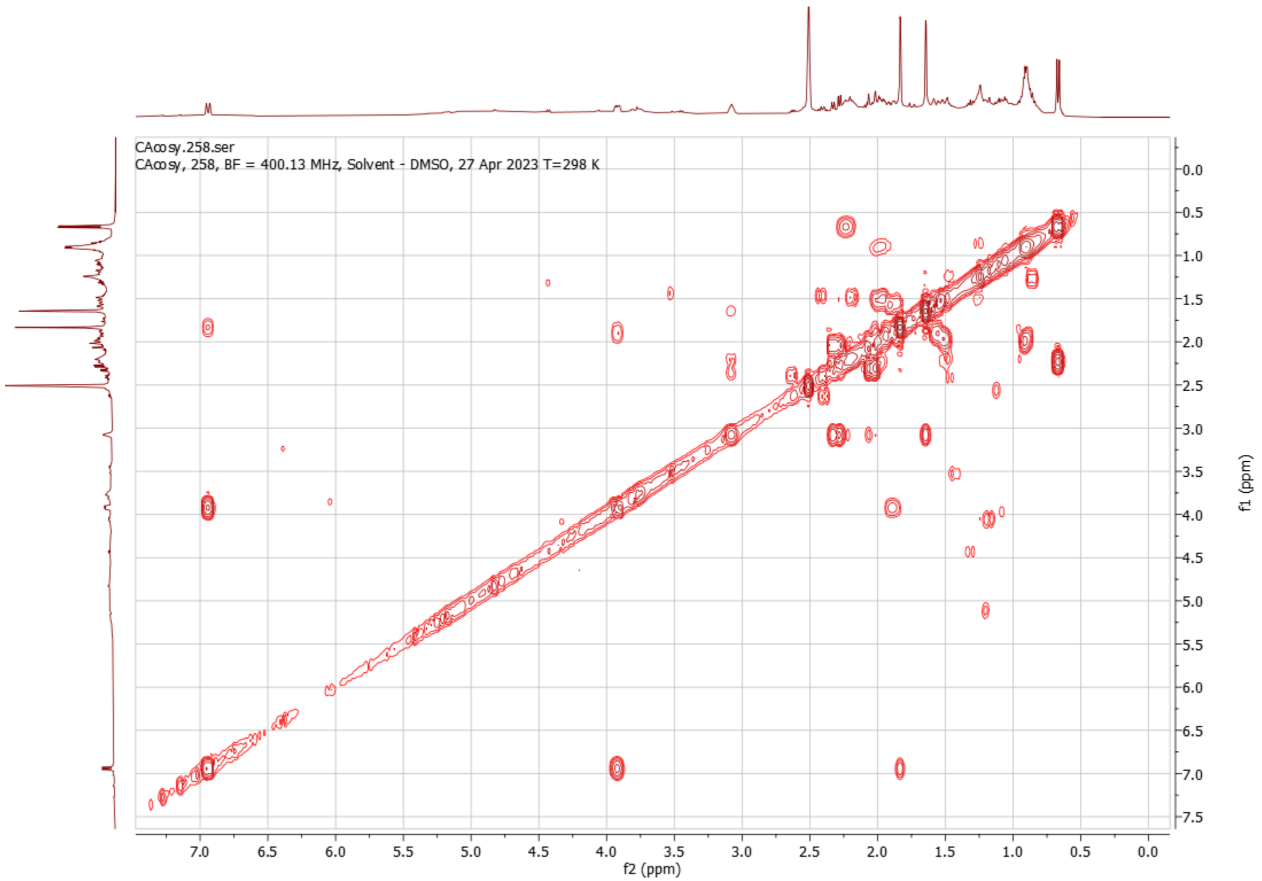

Figure S16. COSY spectrum of compound **3**.

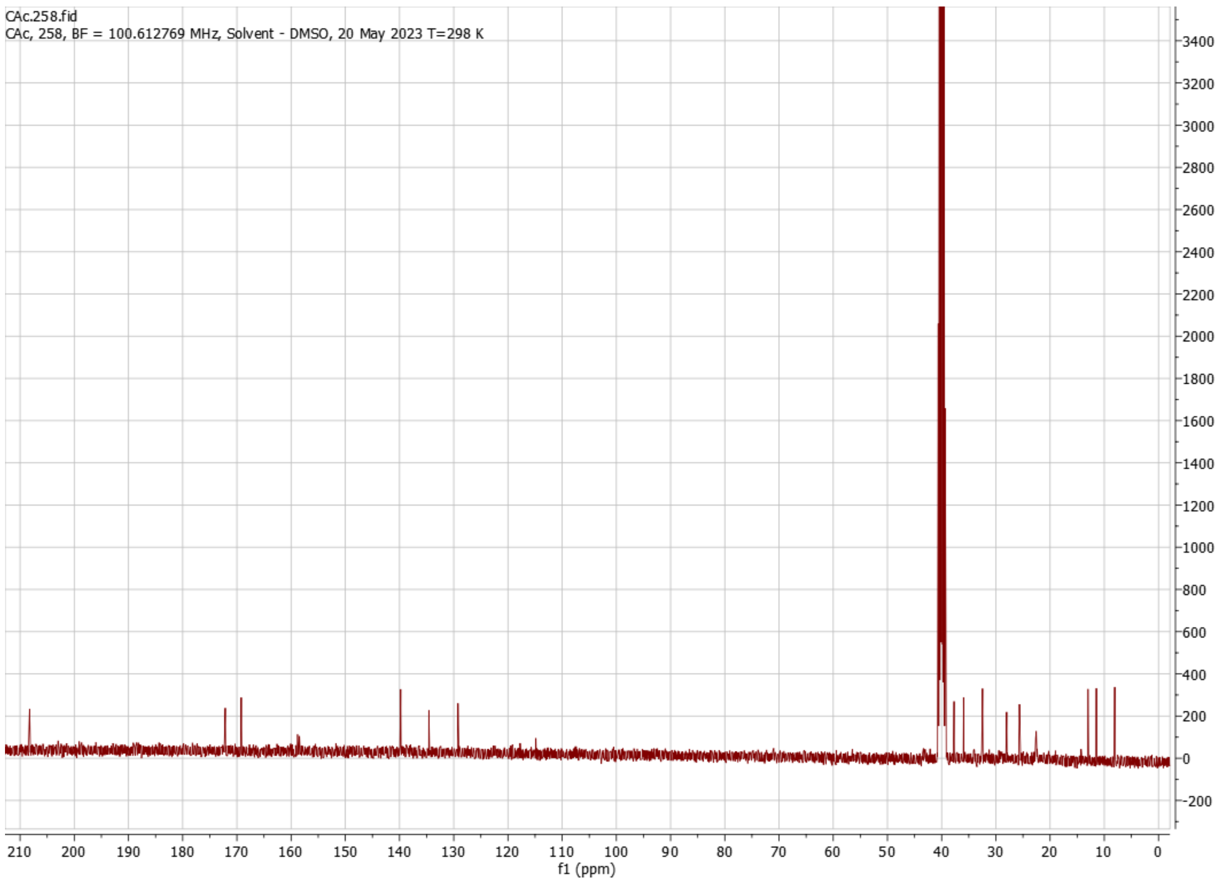

Figure S17. ^{13}C NMR spectrum of compound **3**.

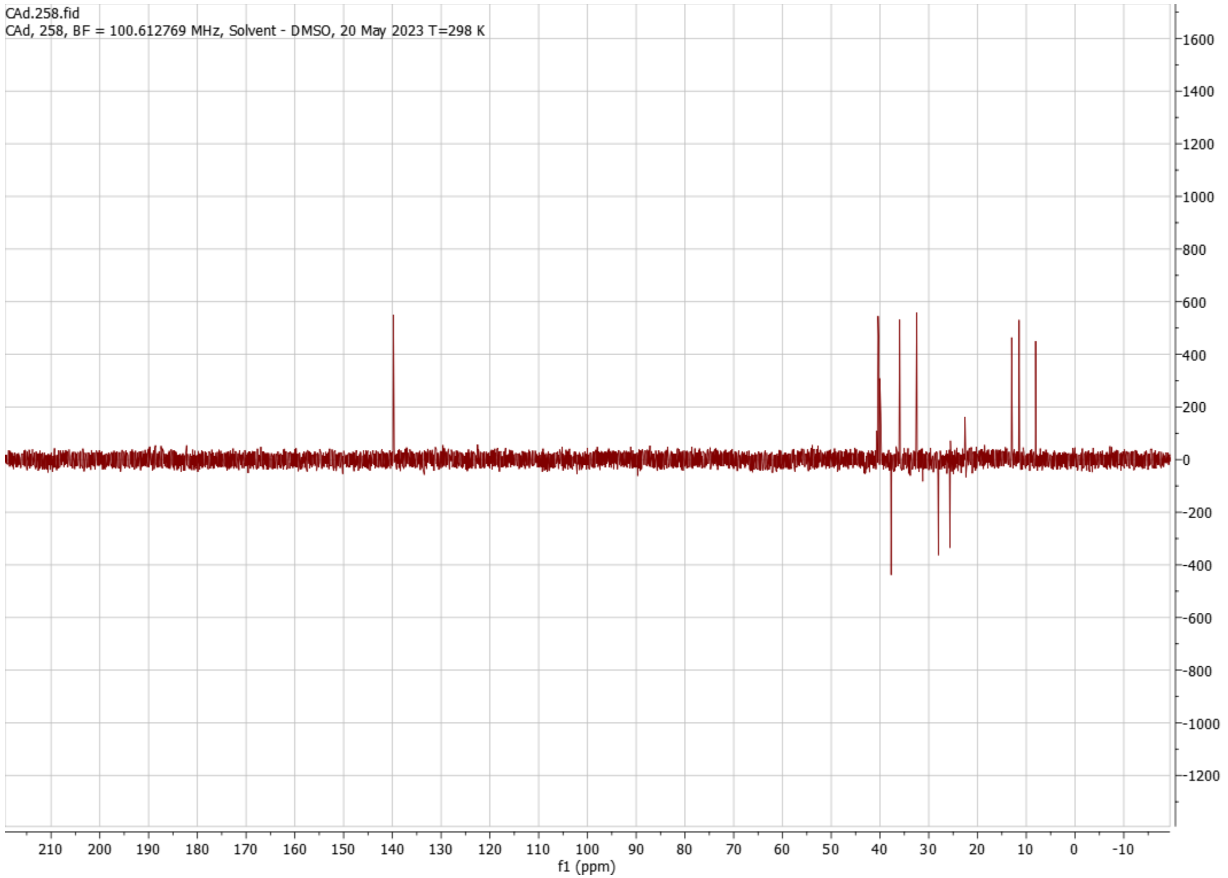

Figure S18. DEPT spectrum of compound **3**.

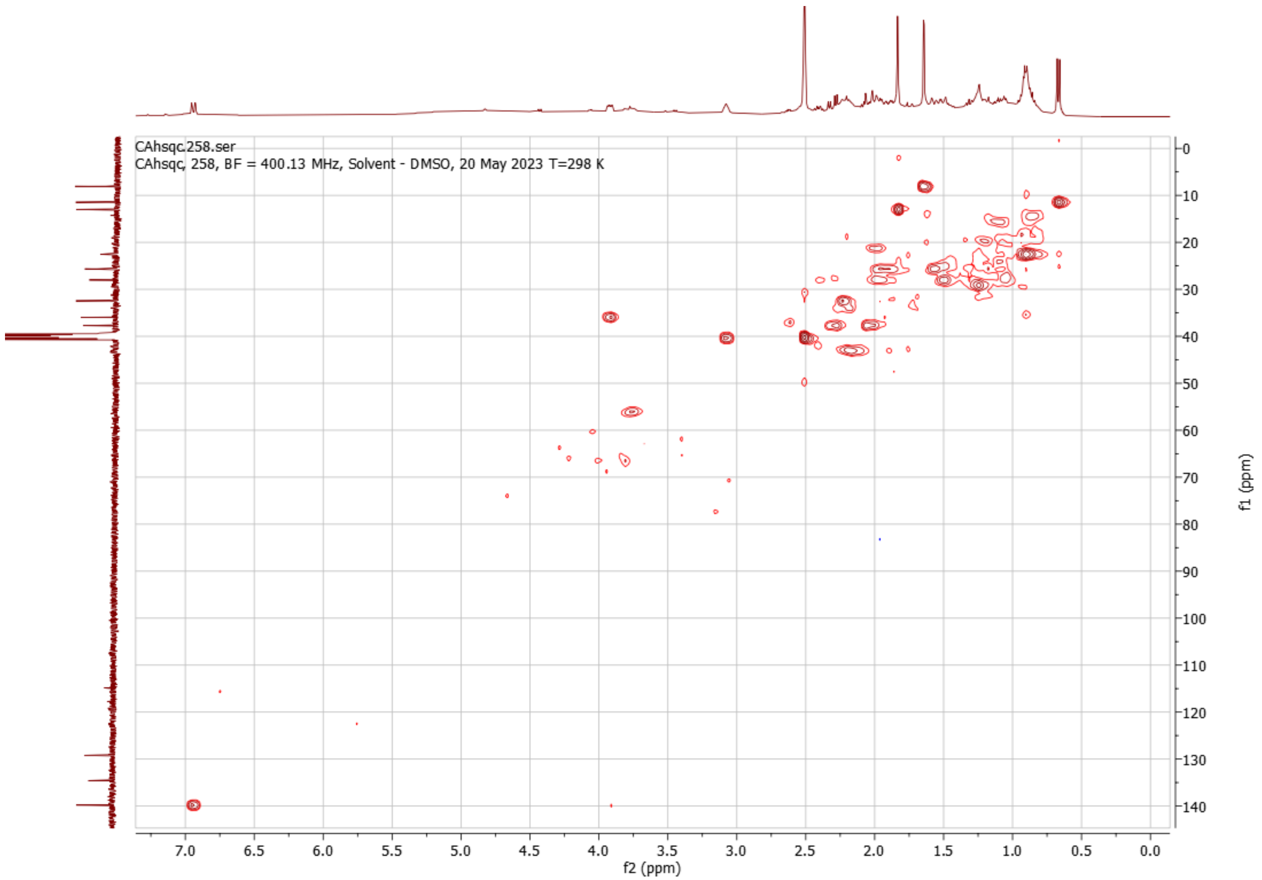

Figure S19. HSQC **s**pectrum of compound **3**.

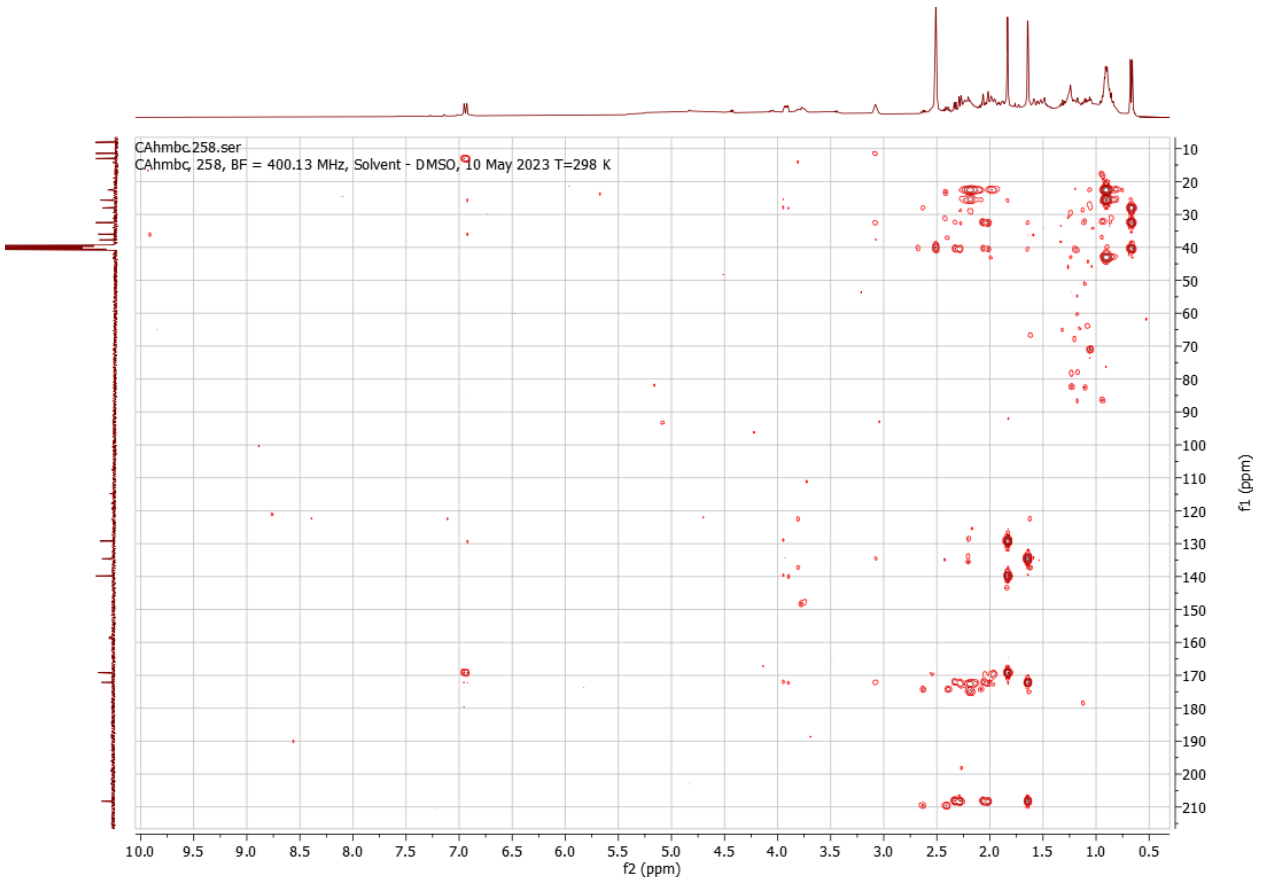

Figure S20. HMBC **s**pectrum of compound **3**.

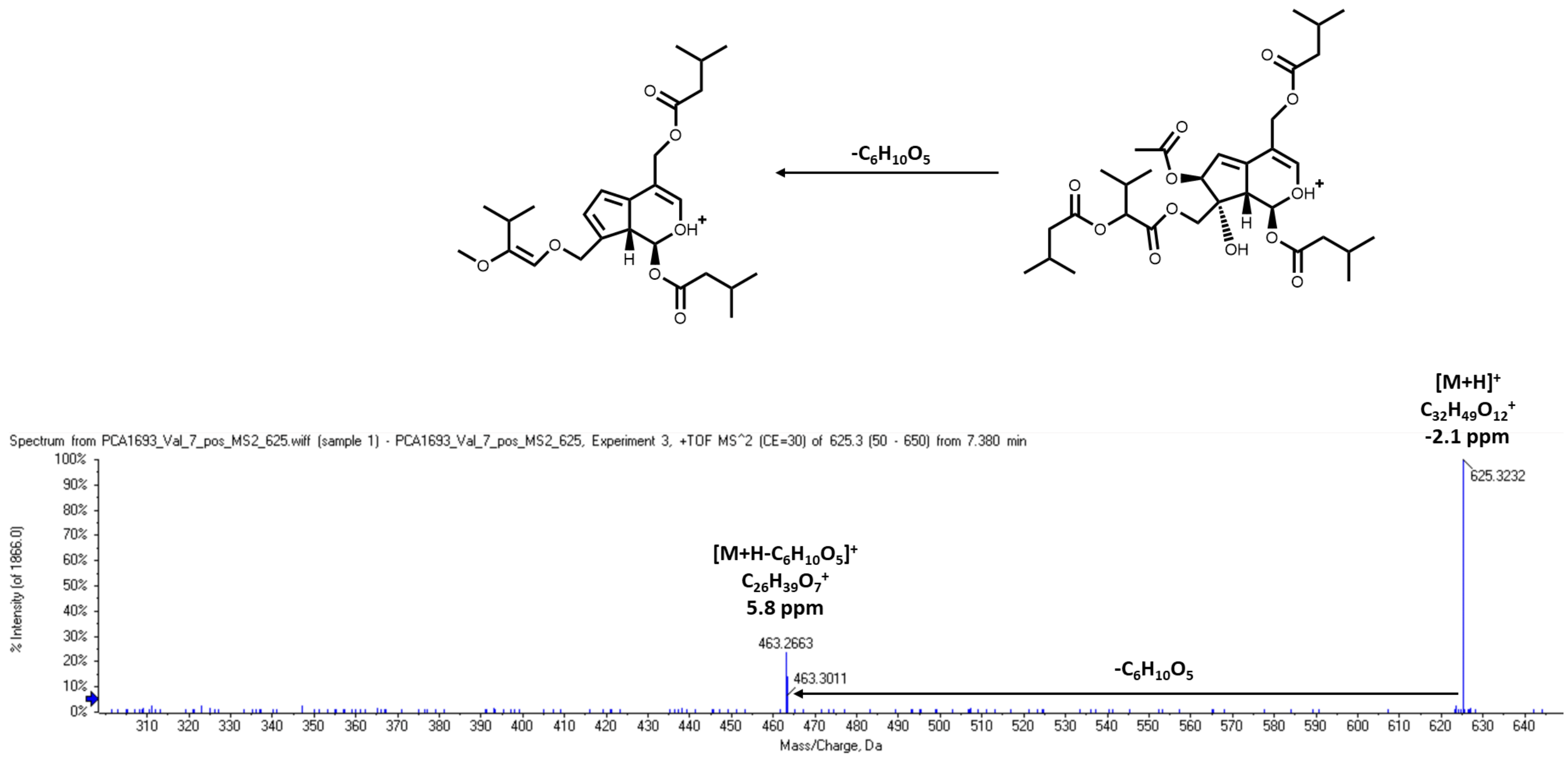

Figure S21. Tandem mass spectrum of *m/z* 625.3 at t_R 7.4 min corresponding to isovaltrate α-(isovaleroxy)isovaleroxyhydrin (**2**) and proposed fragmentation patterns. The spectrum was acquired with a hybrid QqTOF mass spectrometer operated in the positive product ion mode with unit Q1 resolution (collision energy 30 eV).

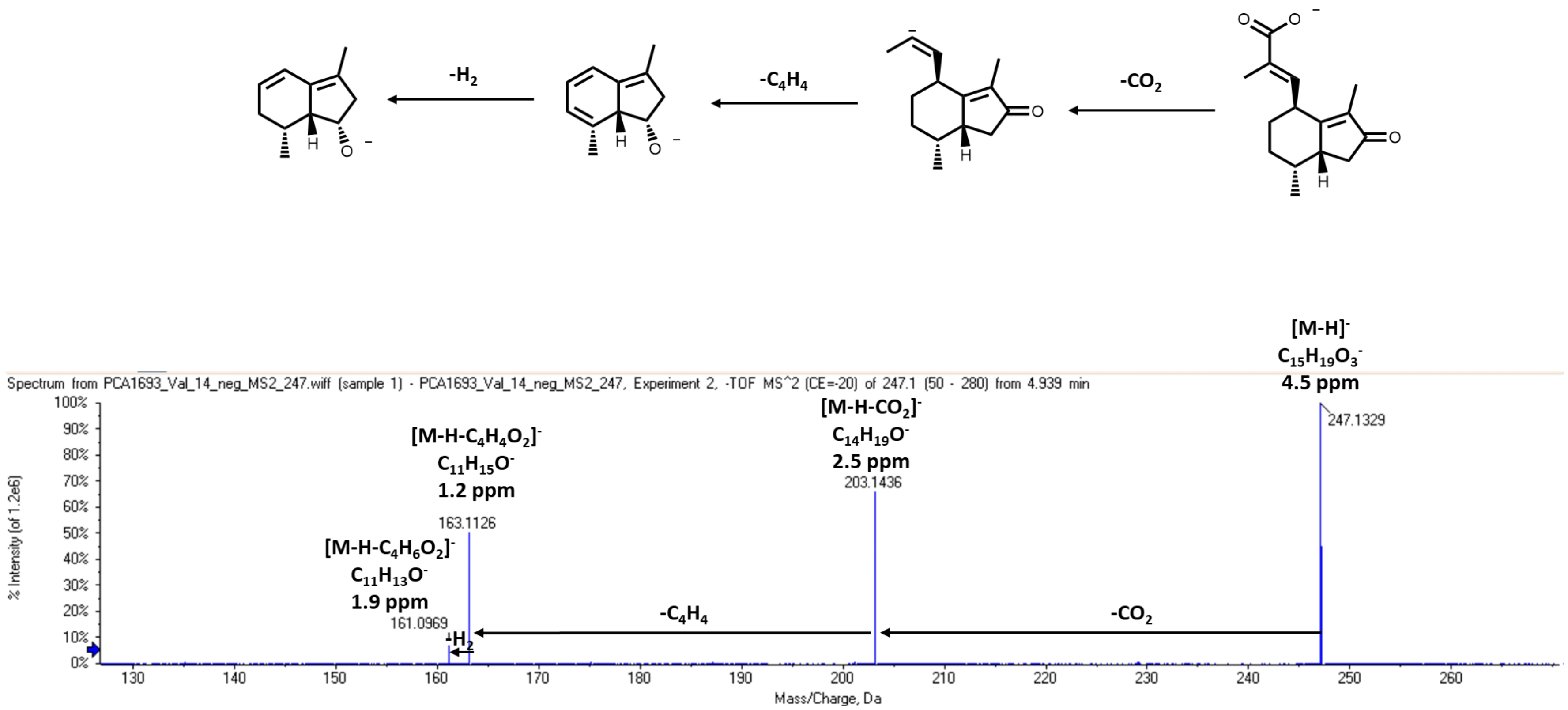

Figure S22. Tandem mass spectrum of *m/z* 247.1 at t_R 4.9 min corresponding to 2-oxovalerenic acid (**3**) and proposed fragmentation patterns. The spectrum was acquired with a hybrid QqTOF mass spectrometer operated in the negative product ion mode with unit Q1 resolution (collision energy 20 eV).

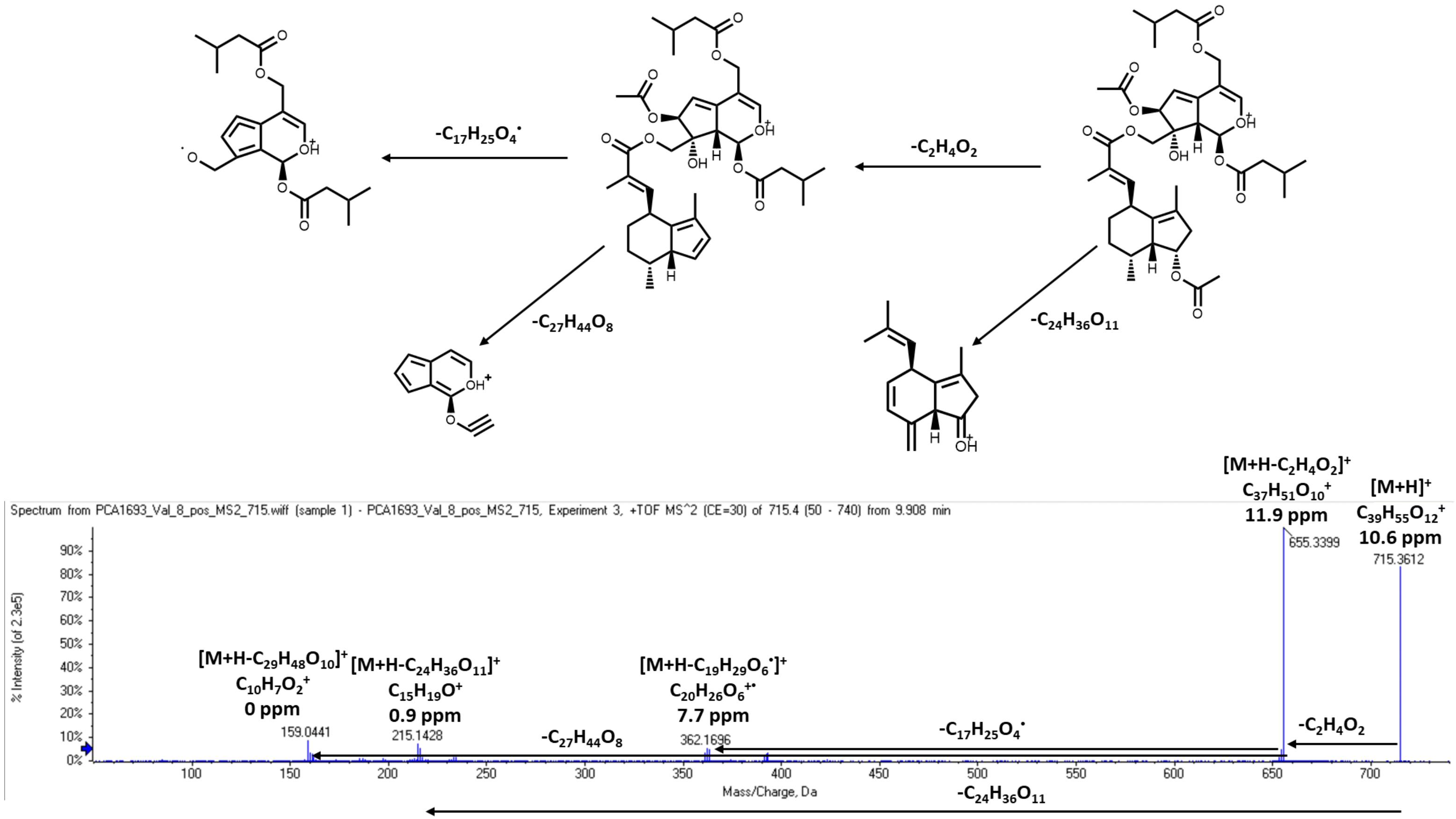

Figure S23. Tandem mass spectrum of *m/z* 715.5 at t_R 9.9 min corresponding to iridoidvol A (**4**) and proposed fragmentation patterns. The spectrum was acquired with a hybrid QqTOF mass spectrometer operated in the positive product ion mode with unit Q1 resolution (collision energy 30 eV).

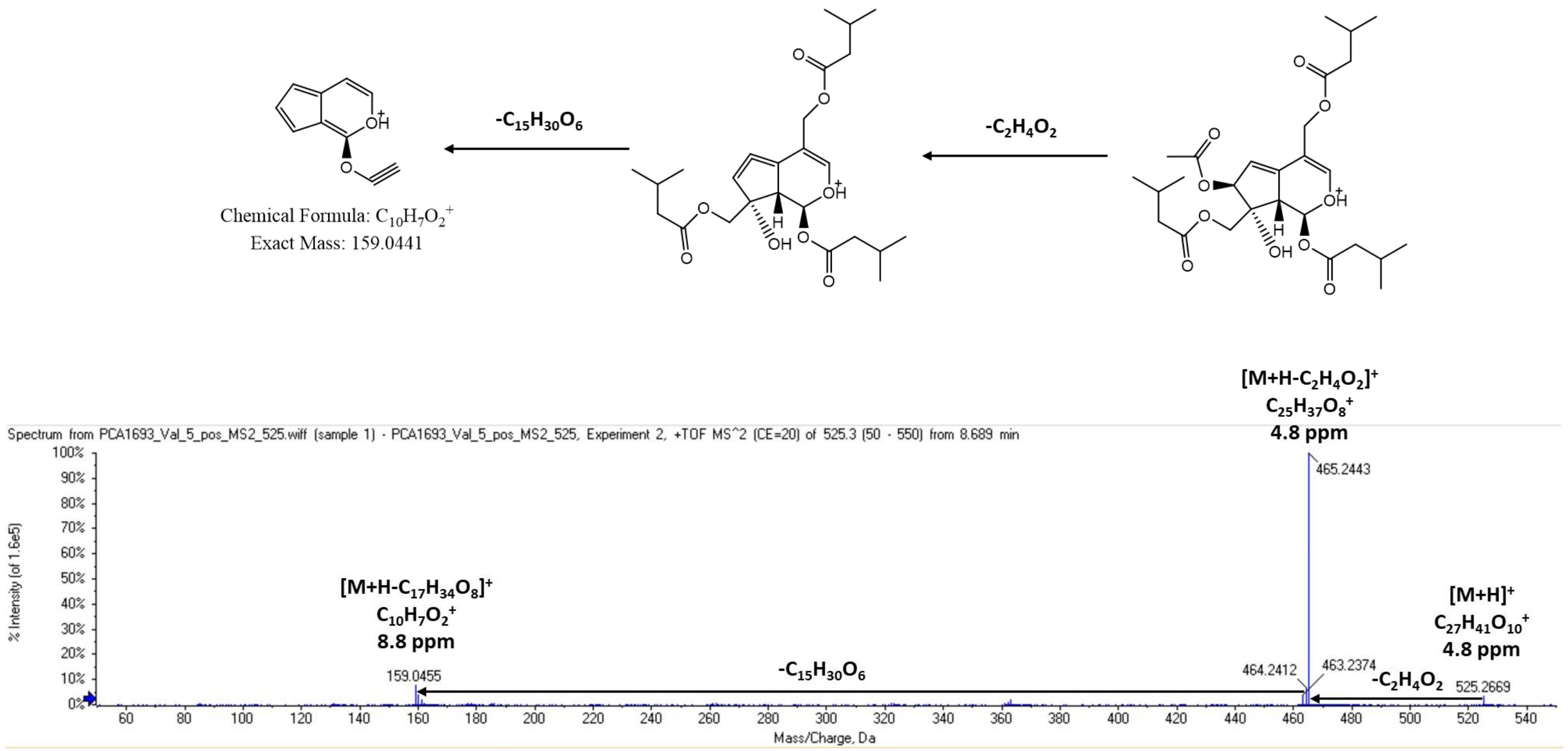

Figure S24. Tandem mass spectrum of *m/z* 525.3 at t_R 8.7 min corresponding to isovaltrate isovaleroyloxyhydrin (**5**) and proposed fragmentation patterns. The spectrum was acquired with a hybrid QqTOF mass spectrometer operated in the positive product ion mode with unit Q1 resolution (collision energy 20 eV).

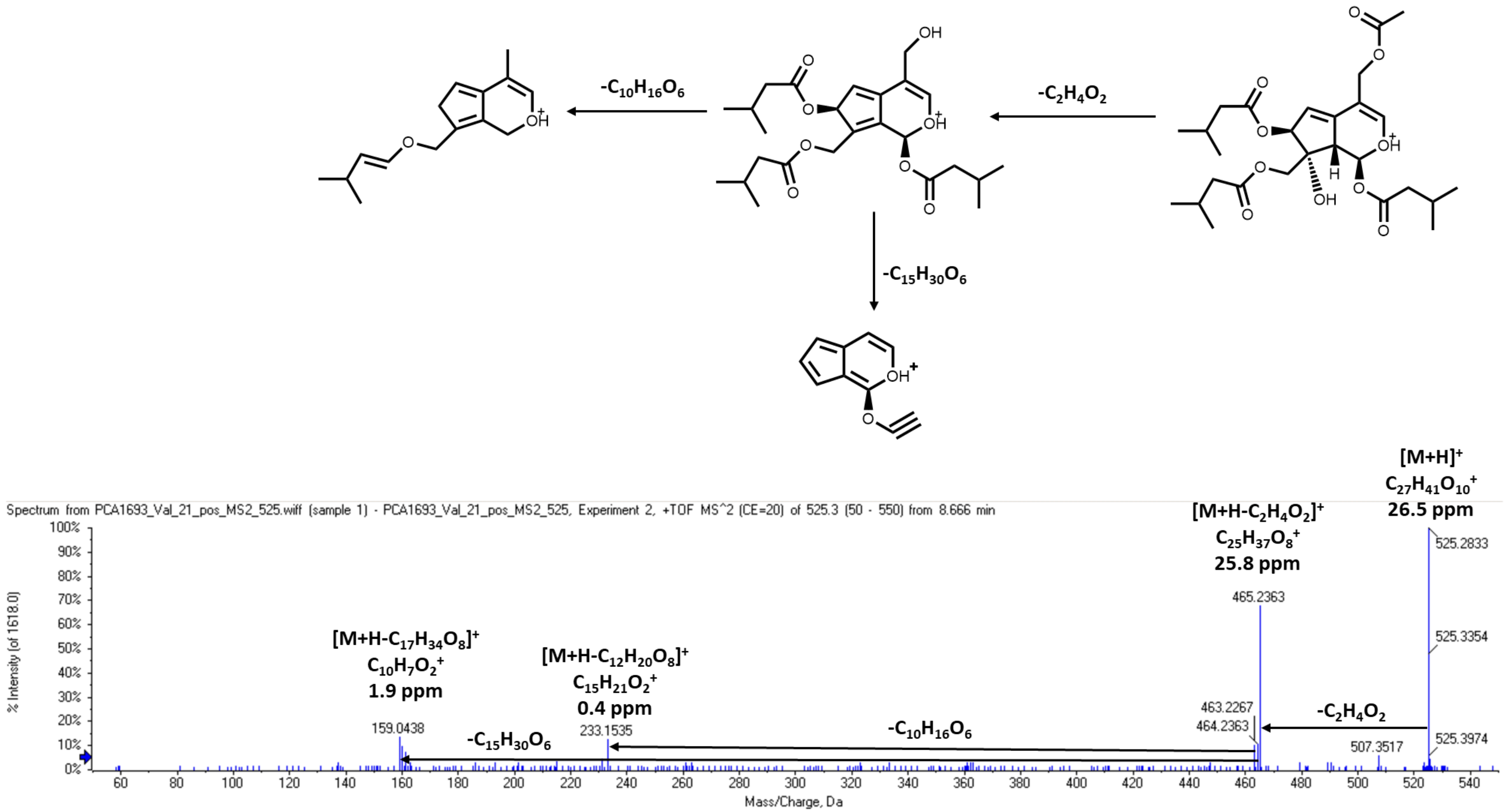

Figure S25. Tandem mass spectrum of *m/z* 525.3 at t_R 8.6 min corresponding to valtrate hydrine B1 (**6**) and proposed fragmentation patterns. The spectrum was acquired with a hybrid QqTOF mass spectrometer operated in the positive product ion mode with unit Q1 resolution (collision energy 20 eV).

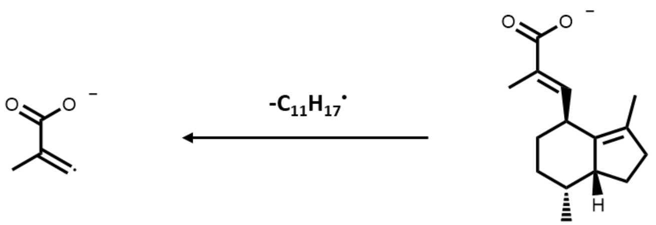

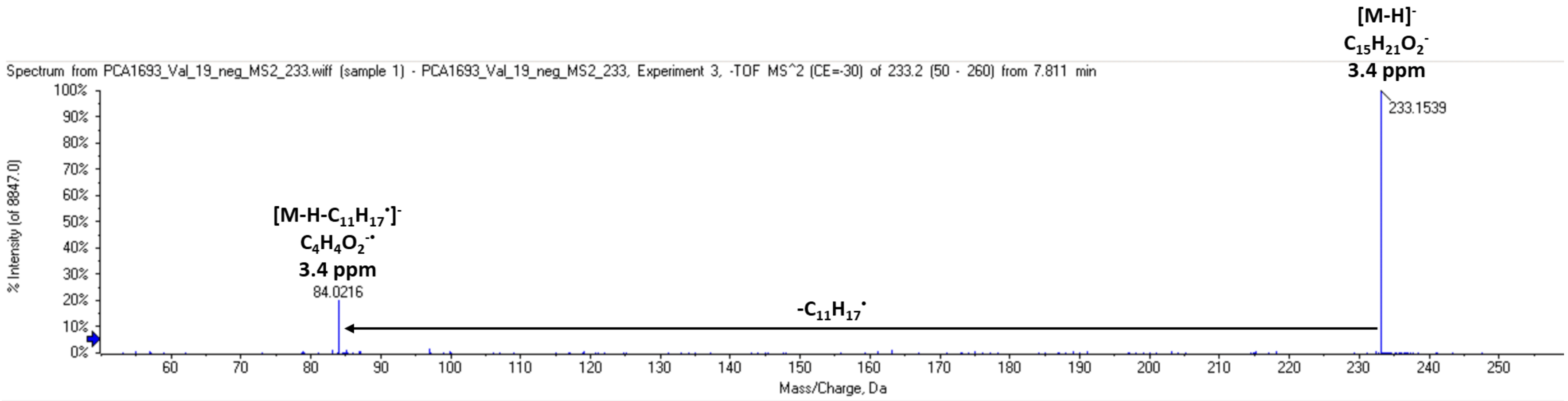

Figure S26. Tandem mass spectrum of *m/z* 233.2 at t_R 7.8 min corresponding to valerenic acid (**7**) and proposed fragmentation patterns. The spectrum was acquired with a hybrid QqTOF mass spectrometer operated in the negative product ion mode with unit Q1 resolution (collision energy 30 eV).

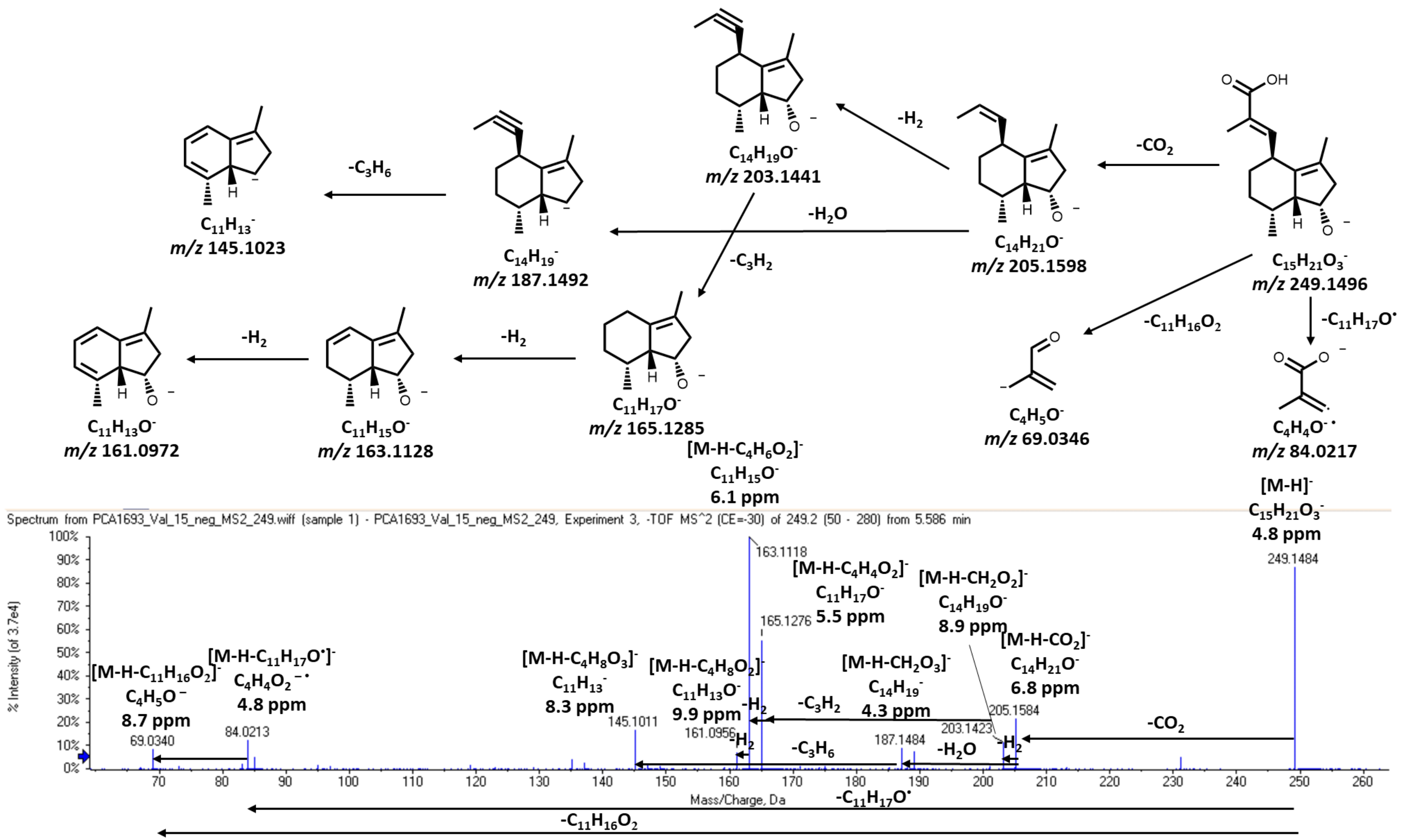

Figure S27. Tandem mass spectrum of *m/z* 249.2 at t_R 5.6 min corresponding to hydroxyvalerenic acid (**8**) and proposed fragmentation patterns. The spectrum was acquired with a hybrid QqTOF mass spectrometer operated in the negative product ion mode with unit Q1 resolution (collision energy 30 eV).

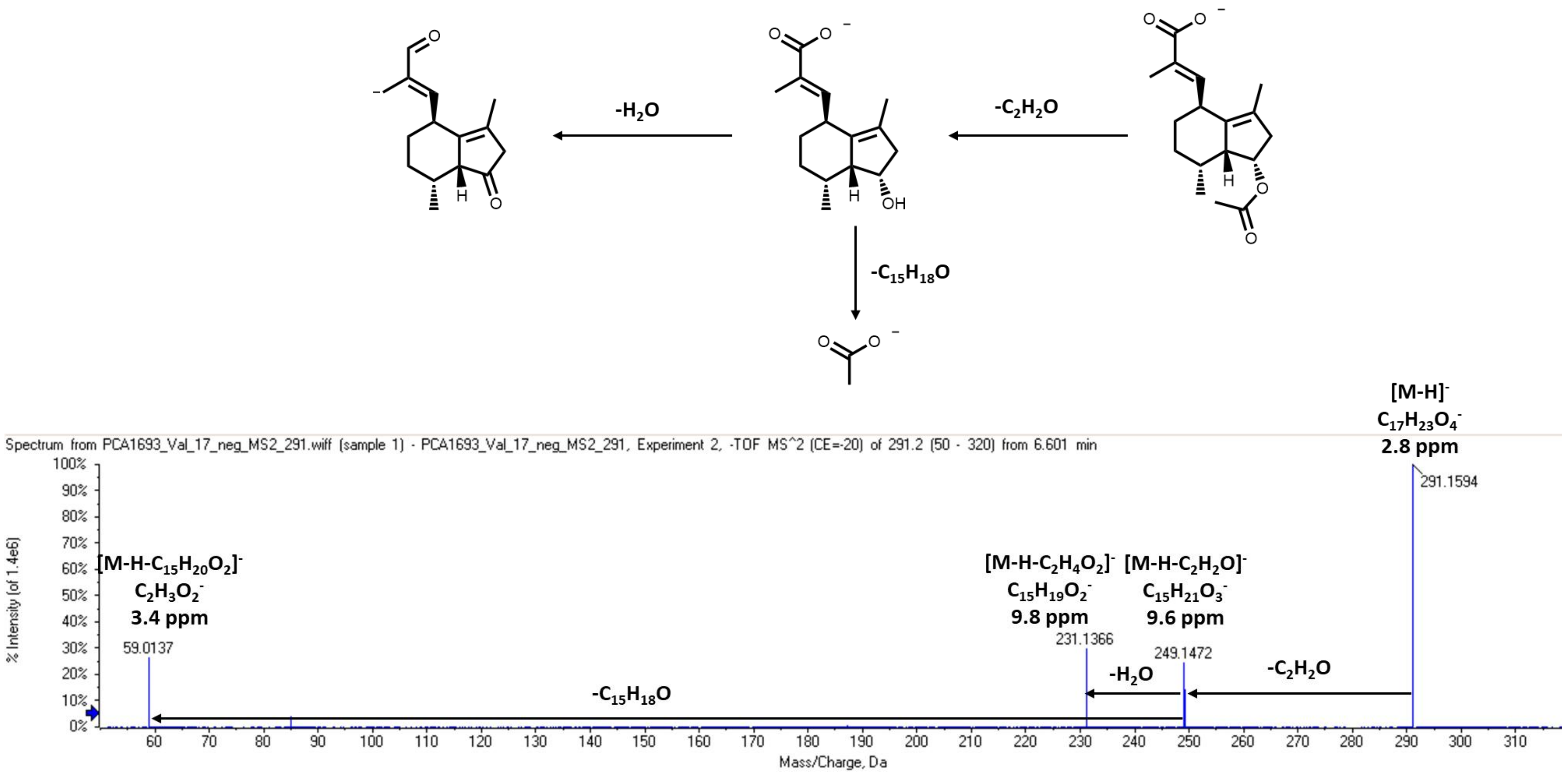

Figure S28. Tandem mass spectrum of *m/z* 291.2 at t_R 6.6 min corresponding to acetoxyvalerenic acid (**9**) and proposed fragmentation patterns. The spectrum was acquired with a hybrid QqTOF mass spectrometer operated in the negative product ion mode with unit Q1 resolution (collision energy 20 eV).

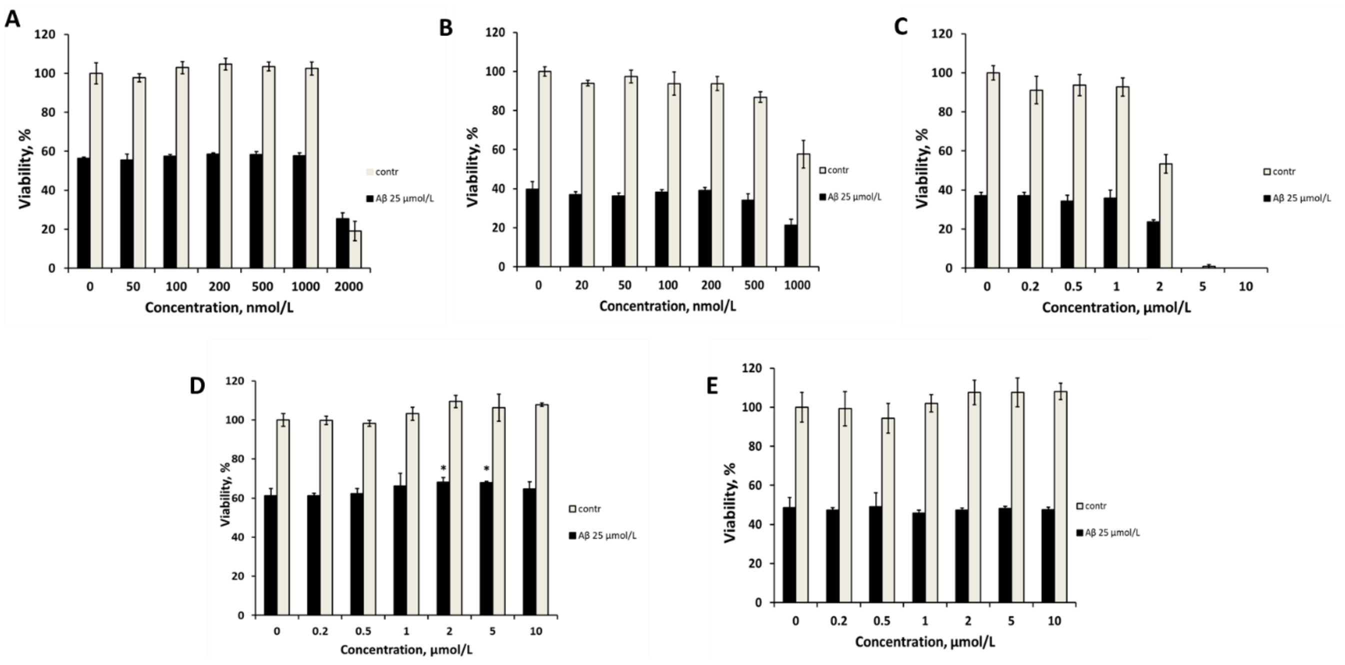

Figure S29. Neuroprotective effects of individual compounds isolated from the roots of *Valeriana officinalis* L. in the model of A*β*25-35 induced cytotoxicity: A. Compound **2**; B. Compound **4**; C. Compound **6**; D. Compound **8**; E. Compound **9**. White bars depict the culture viability under control conditions, i.e. without supplementation of Aβ25-35, black bars depict culture viability after supplementation of 25 *μ*mol/L A*β*25-35. Data are presented as mean ± SD (n=3). * indicates statistical significance of the differences to the controls (cells treated with Aβ25-35 only) at the confidence level of $p \leq 0.05$ for ANOVA and Dunnett post-hoc test, n = 3.

Synthesis Aβ25-35

The resin and all protected amino acids were purchased from Carbolution. Solvents and other reagents were bought from commercial sources (Merck, Carl Roth, CEM, abcr) and used without any further purification.

The fmoc synthesis strategy was used for the solid phase peptide synthesis (SPPS). Loading, capping and coupling was performed with the Liberty Blue Peptide Synthesizer using the standard microwave coupling method (90°C, 2 min, single cycle). *N*-Butyl-2-pyrrolidon (NBP) was used as washing solvent. A mixture of dichloromethane (DCM) and NBP in a ratio of 1:1 was used as transfer and swelling solution and dimethylformamide (DMF) was used as solvent for all the reagents and the reaction solution. Stock solutions were prepared for a 0.1 - 0.5 mmol reaction scale: (i) deprotection Solution: 20% Piperidine in DMF; (ii) amino Acids: 0.2 M of the amino acid in DMF; (iii) activator: 1.0 M Diisopropylcarbodiimid (DIC) in DMF; (iv) activator Base: 1.0 M Oxyma in DMF; (v) capping: 1 M Acetic Anhydride in DMF; (vi) loading: 0.2 M amino acid, 0.2 M HOBt, 0.1 M DIPEA.

Semi preparative RP-HPLC was done on an Äkta pure system equipped with UV-Vis detector (U9-M), a conductivity monitor (C9-M) and a YMC-Pack ODS-A 150 x 20 mm I.D, S-5 μm column. HPLC gradient solvents and water, purified with a GenPure Pro UV-TOC (Thermo Scientific), were used and degassed by ultrasonication for 15 min prior to use.

The resin was prepared on a 0.1 mmol scale using 102 mg dry, unmodified Wang resin (1 eq, 0.98 mmol/g, 100-200 mesh).

The resin was swollen for 10 min in the transfer solvent before it was automatically transferred to the reaction vessel. 2 mL (4 eq) loading solution and second 1 mL (10 eq) activator solution have been added to the Resin, subsequently. The mixture was heated to 90°C using microwave radiation and agitated throughout the reaction by bubbling N_2. The solvent was removed, and the resin washed three times, after which 1 mL (10 eq) capping solution was added. Capping was carried out under analogue conditions.

The loading of the resin was determined as follows:

(i) Weigh duplicate samples of 5 to 10 mg loaded resin in an Eppendorf tube and add 1.0 mL 20% piperidine/DMF, stir for 20 min, and centrifuge the resin.

(ii) Transfer 100 μL of the above solution to a tube containing 10 mL DMF and mix.

(iii) Set the spectrophotometer at 301 nm. Transfer 2 mL DMF into each of the two cells of the spectrophotometer (reference and sample cells), set the spectrophotometer to zero. Empty the sample cell, transfer 2 mL of the solution to measure, and check absorbance.

(iv) Substitution of the resin = [101× (Absorbance)]/[7.8 × (weight in mg)] Check absorbance three times at 301 nm; calculate average substitution.

The microwave SPPS was performed on a 0.25 mmol scale using 540 mg of dry Fmoc-Met-Wang Resin (0.444 mmol/g). The resin was swollen before the first deprotection. Fmoc-deprotection was done in 5 mL solution. For the coupling steps 2 mL activator solution, 1 mL activator base solution and 5 mL amino acid solution was used. The final fmoc-deprotection was performed on resin. The resin was washed three times in between each deprotection and coupling step.

For the cleavage a 10 mL cocktail containing 82.2% (*v/v*) trifluoroacetic acid, 5% (*v/v*) phenol, 5% (*v/v*) water, 5% (*v/v*) thioanisol and 2.5% (*v/v*) ethanedithiol was used. The resin and cleavage cocktail mixture were agitated for 2-3 h at 22°C after which the peptide was precipitated in cold ether and centrifuged. Redispersion and centrifugation in cold ether was repeated twice. Afterwards the crude peptide was dissolved in 30 mL water/acetonitrile (1:1) and lyophilized. The peptide was further purified by RP-HPLC with a gradient: 95:5 (water + 0.1% FA / ACN + 0.1% FA) to 40:60 (water + 0.1% FA / ACN + 0.1% FA) over 15 min. Flow Rate: 15 mL/min.
